# Multilocus phylogeny and ecological differentiation of the “*Eupelmus urozonus* species group” (Hymenoptera, Eupelmidae) in the West-Palaearctic

**DOI:** 10.1186/s12862-015-0571-2

**Published:** 2016-01-19

**Authors:** F. Al khatib, A. Cruaud, L. Fusu, G. Genson, J.-Y. Rasplus, N. Ris, G. Delvare

**Affiliations:** INRA, University of Nice Sophia Antipolis, CNRS, UMR 1355-7254 Institut Sophia Agrobiotech, Sophia Antipolis, 06900 France; INRA, UMR 1062 CBGP, 755 avenue du Campus Agropolis, CS 30016 F-34988, Montferrier-sur-Lez, Cedex France; CIRAD, UMR 55 CBGP, 755 Avenue du Campus Agropolis, CS 30016 F-34988, Montferrier-sur-Lez, Cedex France; Faculty of Biology, Alexandru Ioan Cuza University, Bd. Carol I nr. 11, 700506 Iasi, Romania

**Keywords:** Ecological specialization, Ectoparasitoid, Host range evolution, Molecular phylogeny, Morphological adaptation, Ovipositor, Phylogenetic constraint

## Abstract

**Background:**

The ecological differentiation of insects with parasitic life-style is a complex process that may involve phylogenetic constraints as well as morphological and/or behavioural adaptations. In most cases, the relative importance of these driving forces remains unexplored. We investigate here this question for the “*Eupelmus urozonus* species group” which encompasses parasitoid wasps of potential interest in biological control. This was achieved using seven molecular markers, reliable records on 91 host species and a proxy of the ovipositor length.

**Results:**

After using an adequate partitioning scheme, Maximum likelihood and Bayesian approaches provide a well-resolved phylogeny supporting the monophyly of this species group and highlighting its subdivision into three sub-groups. Great variations of both the ovipositor length and the host range (specialist versus generalist) were observed at this scale, with these two features being not significantly constrained by the phylogeny. Ovipositor length was not shown as a significant predictor of the parasitoid host range.

**Conclusions:**

This study provides firstly the first evidence for the strong lability of both the ovipositor’s length and the realised host range in a set of phylogenetically related and sympatric species. In both cases, strong contrasts were observed between sister species. Moreover, no significant correlation was found between these two features. Alternative drivers of the ecological differentiation such as interspecific interactions are proposed and the consequences on the recruitment of these parasitoids on native and exotic pests are discussed.

**Electronic supplementary material:**

The online version of this article (doi:10.1186/s12862-015-0571-2) contains supplementary material, which is available to authorized users.

## Background

Ecological speciation is a process in which polymorphism within populations (e.g. in resource use or habitat preference) ultimately induces the appearance of two sister species, each adapted to a different niche [1–4]. According to Rundle and Nosil [2], three principal components must be involved: i) a source of divergent selection, ii) a form of reproductive isolation, and iii) a genetic mechanism linking divergent selection to reproductive isolation. Among plant-feeding insects, several empirical studies support this scenario [1, 5, 6], which can also occur for insects with a parasitic lifestyle, in particular within the upper trophic levels. For such organisms, ecological differentiation between sister species can also be driven by the ecological differentiation of their hosts *via* a process called sequential or cascading speciation [7–9]. If pervasive enough, such processes should lead to the clustering of phylogenetically related specialists.

Additionally, transitions between generalists to specialists (and vice-versa) are also occurring and, so far, empirical data provide a mixed picture about the relative frequencies of evolution toward specialisation and generalization [10–13]. However, transitions from generalist ancestors to specialized species are probably recurrent as (i) generalist species are unlikely to produce “jack-of-all trades-master of none” genotypes because of genetic or physiological trade-off [14–16]; (ii) the subsequent acquisition of specialized genotypes may be a primary step towards speciation [17–19]; and (iii) specialist species may be more prone to extinction [13, 20]. At a phylogenetic level, both kinds of transitions should lead to the mixing of both specialists and generalists within the same cluster.

Questions of (i) the host range (specialist versus generalist) of ancestral species of current specialists and (ii) the distribution of host ranges within a phylogeny were recently addressed by Hardy and Otto [21]. They illustrated them using two notions, respectively “the musical chairs hypothesis” (specialists originate from specialists through host switch) and the “oscillation hypothesis” (specialists originate from generalists, with some specialists widening their host range before the next speciation event). The extent to which one of these scenarios is more frequent has nevertheless still to be evaluated rigorously for the organisms with a parasitic lifestyle.

Parasitoids are organisms (mainly Hymenoptera and Diptera) whose pre-imaginal life depends on the successful exploitation of a single host [22, 23]. Behind this simple definition, a great diversity of life history strategies and physiological adaptations are observed. In particular, the ovipositor allows egg-laying by the female and is thus a key organ especially for species that are exploiting concealed or protected hosts [24, 25]. The features (in particular the length) of this organ and its ability to evolve could contribute to drive specialization and/or speciation. Focusing on the “*Eupelmus urozonus* species group” (Hymenoptera: Eupelmidae), we examine here whether the host range is subject to phylogenetic constraints and/or whether the ovipositor length is a significant driver of host use.

Within the subfamily Eupelminae (33 genera), the genus *Eupelmus* Dalman is the most diverse, with 91 available valid species names in the Palaearctic region [26]. Species of *Eupelmus* are primary or facultative secondary ectoparasitoids whose larvae develop as idiobionts on the immature stages (larvae, pupae and more rarely eggs) of many insects (beetles, flies, moths, wasps or cicadas) that are concealed or protected in plant tissues (stems, galls, fruits or seeds) [27]. Most *Eupelmus* are considered as generalist parasitoids [27, 28]. However, because of both the extreme sexual dimorphism characterizing the subfamily and the existence of species groups possibly hiding cryptic species, the systematics and the evolutionary ecology of these species remain poorly understood. This situation is well illustrated with the “*E. urozonus* species complex/group” which was repeatedly investigated [27, 29–31] until its recent revision within the Palaearctic region by Al khatib et al. [32, 33], which identified 11 new species in this region. Semantically, the term “complex” used in Al khatib et al. [32, 33] is substituted here by the term “species group” (Al khatib et al. in preparation). As a consequence of this unsuspected biodiversity, most of the published host records for these species are unreliable because all of the common species with a comparatively short ovipositor (*E. gemellus* Al khatib, 2015, *E. confusus* Al khatib, 2015, and especially *E. kiefferi* De Stefani, 1898) were misidentified as *E. urozonus* Dalman, 1820, while the two common species with a comparatively long ovipositor (*E. azureus* Ratzeburg, 1844 and *E. annulatus* Nees, 1834) were both frequently mistreated under *E. annulatus* [29, 34].

In the present study, we first provide a reliable molecular phylogeny of the “*E. urozonus* species group” using a multi-locus approach. Then, for most of the species, we compile host records and data on ovipositor length. We finally carry out a comparative analysis to evaluate the role of phylogenetic constraints in the evolution of ovipositor length and host range as well as the role of the ovipositor’s length in determining the host range.

## Methods

### Sampling

A total of 31 species, with 91 individuals, sampled in the Palaearctic region were included in this study.

- Eighteen of the 21 species within the “*urozonus* species group” that were recently revised using both morphological and molecular characters [32, 33]: *E. acinellus* Askew, 2009, *E. annulatus*, *E. azureus*, *E. cerris* Förster, 1860, *E. confusus*, *E. fulvipes* Förster, 1860, *E. gemellus*, *E. janstai* Delvare and Gibson, 2015, *E. kiefferi*, *E. longicalvus* Al khatib & Fusu, 2015, *E. minozonus* Delvare, 2015, *E. opacus* Delvare, 2015, *E. pistaciae* Al khatib, 2015, *E. priotoni* Delvare, 2015, *E. purpuricollis* Fusu & Al khatib, 2015, *E. simizonus* Al khatib, 2015, *E. tibicinis* Bouček, 1963 and *E. urozonus*.
- Thirteen species were used as outgroup including (i) species belonging to the three subgenera of *Eupelmus* sensu Gibson (1995): *Eupelmus* [*E. atropurpureus* Dalman, 1820, *E. matranus* Erdős, 1947, *E. microzonus* Förster, 1860, *E. pini* Taylor, 1927 and *E. vindex* Erdős, 1955]; *Macroneura* Walker [*E. falcatus* (Nikol’skaya, 1952) and *E. seculatus* Kalina, 1981], and *Episolindelia* Girault [*E. linearis* Förster, 1860, *E. testaceiventris* (Motschulsky, 1863) and *E. juniperinus thuriferae* Askew, 2000]; and (ii) species belonging to other genera within Eupelminae, *Reikosiella* (*Hirticauda*) [*R. aff. rostrata* (Ruschka, 1921)] and *Anastatus* Motschulsky [*Anastatus sidereus* (Erdős, 1957) and *Anastatus aff. temporalis* Askew, 2005]. The species were identified by the authors using the available identification keys [29, 31, 35–37].

Specimens were killed with ethyl acetate and preserved in 95 % ethanol at −20 °C until DNA extraction. After the DNA extraction, the voucher specimens were prepared as explained in Al khatib et al. (2014) for the morphological examination. The vouchers are deposited in the following institutions and private collections: AICF, Lucian Fusu collection, Al. I. Cuza University, Iasi, Romania; BMNH, Natural History Museum, London, UK; CBGP, Centre for Biology and Management of Populations, Montpellier, France; CNC, Canadian National Collection of Insects, Arachnids and Nematodes, Agriculture & Agri-food Canada, Ottawa, ON, Canada; FALPC, Fadel Al khatib personal collection, Faculty of Agricultural Engineering, University of Aleppo, Syria; GDPC, Gérard Delvare personal collection, Montpellier, France; MNHG, Museum of Natural History of Geneva, Switzerland; MNHN, National Museum of Natural History, Paris, France; NHRS, Naturhistoriska riksmuseet, Stockholm, Sweden. The depository’s acronyms of voucher specimens are included in (Additional file 1: Table S2; Additional file Dryad: doi:10.5061/dryad.115m1). Sampling information (host-plants, collection dates, and localities) is listed in Table 1.

**Table 1:** Sample information for the specimens included in the phylogenetic analysis

Species	Collection code	Molecular code	Country	Department	City	N°	E°	Host insect	Associated plant	Collection date
*Eupelmus* * acinellus*	FAL1363	10235	France	Aude	Durban-Corbières	42.99825°	2.80690°	*Mesophleps oxycedrella*	*Juniperus oxycedrus*	March 2012
*Eupelmus * *acinellus*	FAL1366	10237	France	Var	Fayence	43.65513°	6.68813°	*Mesophleps oxycedrella*	*Juniperus oxycedrus*	March 2012
*Eupelmus * *annulatus*	FAL1176	10198	France	Alpes-Maritimes	Gréolières-les-Neiges	43.81584°	6.88711°	*Diplolepis rosae*	*Rosa canina*	March 2012
*Eupelmus * *annulatus*	NB783	10354	France	Gard	Le Castanet	43.98925°	3.70094°	*Dryocosmus kuriphilus*	*Castanea sativa*	February 2012
*Eupelmus * *annulatus*	GDEL4053	10041	Hungary	Veszprém	Hegyesd	46.933333°	17.522778°	Unknown	On *Quercus cerris*	June 2010
*Eupelmus * *annulatus*	LF.an.SW 01	10471	Sweden	Öland	Mörbylånga	56.61670°	16.507617°	Unknown	Unknown	August 2006
*Eupelmus * *azureus*	FAL1323	10222	France	Ardèche	Saint-Georges-les-Bains	44.85028°	4.82433°	*Biorhiza pallida*	*Quercus pubescens*	June 2012
*Eupelmus * *azureus*	NB773a	10361	France	Var	La Garde-Freinet	43.30487°	6.43701°	*Dryocosmus kuriphilus*	*Castanea sativa*	February 2012
*Eupelmus * *azureus*	GDEL4048	10034	Italy	Piemonte/Cuneo	Palanfré	44.165833°	7.50361°1	Unknown	Unknown	August 2010
*Eupelmus * *azureus*	L.Loru713	10245	Italy	Sardinia	Aritzo	39.94743°	9.19968°	*Dryocosmus kuriphilus*	*Castanea sativa*	August 2011
*Eupelmus * *azureus*	PJ10077-21-4	10575	Hungary	Vezprém	Várpalota	47.19809°	18.21204°	*Andricus solitarius*	*Quercus pubescens*/*Q. cerris*	June 2010
*Eupelmus * *azureus*	PJ11054-2-2	10578	Turkey	Bursa	Güneybudaklar	40.00560°	29.14982°	*Andricus fecundator*	*Quercus* sp.	-
*Eupelmus * *azureus*	MC-C4	10486	Switzerland	Stabio	Via Roccoletta	45.84722°	8.92638°	*Dryocosmus kuriphilus*	*Castanea sativa*	August 2012
*Eupelmus * *cerris*	GDEL4109	10118	Hungary	Vezprém	Hegyesd	46.93333°	17.52278°	Unknown	On *Quercus cerris*	June 2010
*Eupelmus * *confusus*	FAL1278	10443	France	Ardèche	Saint-Georges-Montpellier	43.6104°	3.77227°	*Bactrocera oleae*	*Olea europaea*	October 2011
*Eupelmus * *confusus*	FAL1519	10412	France	Haute-Corse	Lumio	42.55879°	8.81299°	*Bactrocera oleae*	*Olea europaea*	September 2012
*Eupelmus * *confusus*	FAL1051	10145	Italy	Liguria	Bussana-Vecchia	43.84026°	7.82905°	*Myopites stylata*	*Dittrichia viscosa*	January 2011
*Eupelmus * *confusus*	FAL1108	10250	Spain	Logroño	La Rioja	-	-	*Myopites stylata*	*Dittrichia viscosa*	March 2012
*Eupelmus * *confusus*	LF.ma.GR 01	10425	Greece	Seres	Kerkini Lake Nat.Park	41.27833°	23.21955°	Unknown	Unknown	June 2008
*Eupelmus * *confusus*	LF.ma.GR 02	10426	Greece	Seres	Kerkini lake	41.20180°	23.07747°	Unknown	Unknown	September 2007
*Eupelmus * *confusus*	GDEL4173	10596	France	Hérault	Laroque	45.91722°	3.74361°	Unknown	On *Quercus pubescens*	July 2013
*Eupelmus * *confusus*	LF.ma.IR 05	10424	Iran	Kerman	Bidkhan	29.59725°	56.48600°	Unknown	On *Salix alba*	May 2012
*Eupelmus * *confusus*	LF.ma.CY 01	10427	Cyprus	Lemesos	Lemesos	34.73189°	33.05175°	*Apomyelois ceratoniae & Asphondylia gennadii*	*Ceratonia siliqua*	May 2009
*Eupelmus * *fulvipes*	FAL1221	10200	France	Alpes-Maritimes	Gréolières-les-Neiges	43.81584°	6.88711°	*Diplolepis rosae*	*Rosa canina*	March 2012
*Eupelmus * *fulvipes*	LF.ro.RO 02	10656	Romania	Constanţa	Hagieni & Negru Voda	-	-	*Diplolepis spinosissimae*	*Rosa* sp.	-
*Eupelmus * *fulvipes*	LF.ro.GE 01	10657	Germany	Rottenburg-Wurmlingen		-	-	*Diplolepis rosae*	*Rosa* sp.	October 2011
*Eupelmus * *gemellus*	FAL1260	10438	France	Var	Porquerolles	42.99534°	6.2044°	*Bactrocera oleae*	*Olea europaea*	-
*Eupelmus * *gemellus*	FAL1359	10230	France	Alpes-Maritimes	Biot	43.63455°	7.082490°	*Mesophleps oxycedrella*	*Juniperus oxycedrus*	March 2012
*Eupelmus * *gemellus*	NB441	10415	France	Haute-Corse	Bisinchi	42.48983°	9.32797°	*Dryocosmus kuriphilus*	*Castanea sativa*	June 2012
*Eupelmus * *gemellus*	FAL1004	10130	Italy	Liguria	Bussana-Vecchia	43.84026°	7.82905°	*Myopites stylata*	*Dittrichia viscosa*	January 2011
*Eupelmus * *gemellus*	FAL1508	10405	Italy	Sardinia	Province d’Oristano	39.70041°	8.739690°	Unknown	On *Pistacia lentiscus*	October 2012
*Eupelmus * *janstai*	GDEL4046	10032	Czech Republic	Břeclav	Pavlov	48.867500°	16.654166°	Unknown	On *T. platyphyllos*	July 2010
*Eupelmus * *kiefferi*	NB674b	10341	France	Alpes-Maritimes	Granile	44.03942°	7.57575°	*Dryocosmus kuriphilus*	*Castanea sativa*	March 2012
*Eupelmus * *kiefferi*	NB666	10325	France	Haute-Corse	Muratu	42.55139°	9.30929°	*Dryocosmus kuriphilus*	*Castanea sativa*	December 2012
*Eupelmus * *kiefferi*	FAL1070	10151	Italy	Liguria	Bussana-Vecchia	43.84026°	7.82905°	*Myopites stylata*	*Dittrichia viscosa*	January 2012
*Eupelmus * *kiefferi*	FAL1109	10167	Spain	Logroño	La Rioja	-	-	*Myopites stylata*	*Dittrichia viscosa*	March 2012
*Eupelmus * *kiefferi*	FAL1511	10406	Lebanon	Bakhoun	Fanar	-	-	*Myopites stylata*	*Dittrichia viscosa*	March 2012
*Eupelmus * *kiefferi*	GDEL4045	10030	Hungary	Szombathely	Köszeg	47.363888°	16.52500°	Unknown	On *Salix cinerea*	June 2010
*Eupelmus * *kiefferi*	MC-C124	10492	Switzerland	Riviera	Monte Ceneri	46.136944°	08.902500°	*Dryocosmus kuriphilus*	*Castanea sativa*	July 2012
*Eupelmus * *kiefferi*	LF.ma.RO 01	10423	Romania	Botoşani	Leorda	-	-	Unknown	Unknown	July 2007
*Eupelmus * *kiefferi*	ZL.fu.RO 05	10585	Romania	Mures	Sovata	46.54482°	24.96769°	*Diplolepis mayri*	*Rosa canina*	March 2012
*Eupelmus * *kiefferi*	LF.fu.GE 02	10658	Germany	Rottenburg-Wurmlingen		-	-	*Diplolepis rosae*	*Rosa* sp.	October 2013
*Eupelmus * *kiefferi*	LF.fu.SL 01	10467	Slovakia	Muranska Planina	Predna Hora	-	-	Unknown	Unknown	July 2009
*Eupelmus * *kiefferi*	GDEL4043	10028	Czech Republic	Trutnov	Vilantice	50.365833°	15.737222°	Unknown	Unknown	July 2010
*Eupelmus * *kiefferi*	LF.fu.ES 01	10463	Estonia	Tartu	Rannu Parish	-	-	Unknown	Unknown	June 2010
*Eupelmus * *kiefferi*	FAL1524	10593	Algeria	Tigzirt	Tigzirt	-	-	*Myopites stylata*	*Dittrichia viscosa*	Februry 2013
*Eupelmus * *longicalvus*	GDEL4038	10019	Italy	Friuli Venezia Giulia	Chiusaforte	46.405277°	13.445000°	Unknown	Unknown	July 2008
*Eupelmus * *longicalvus*	LF.ma.SW 02	10429	Sweden	Gotland	Gotlands commun	57°32.207’	18°20.273’	Unknown	Unknown	July 2004
*Eupelmus * *longicalvus*	GDEL4191	10603	Italy	Friuli-Venezia Giulia	Chiusaforte	46.39944°	13.45944°	Unknown	Unknown	July 2008
*Eupelmus * *minozonus*	GDEL4030	10009	Hungary	Veszprém	Hegyesd	46.93333°	17.52278°	Unknown	On *Quercus cerris*	June 2010
*Eupelmus * *minozonus*	GDEL4030	10010	Hungary	Veszprém	Hegyesd	46.93333°	17.52278°	Unknown	On *Quercus cerris*	June 2010
*Eupelmus * *minozonus*	GDEL4030	10011	Hungary	Veszprém	Hegyesd	46.93333°	17.52278°	Unknown	On *Quercus cerris*	June 2010
*Eupelmus * *opacus*	LF.ur.GR 01	10459	Greece	Seres	Krousia Mts site	41°11’32,4”	23°03’59,5”	Unknown	Unknown	June 2007
*Eupelmus * *opacus*	LF.ur.SW 02	10460	Sweden	Östergötland	Ödeshögs kommun	58°18.452’	14°37.859’	Unknown	Unknown	August 2005
*Eupelmus * *pistaciae*	GDEL4027	10004	France	Hérault	Cazevieille	43.752222°	3.770000°	*Megastigmus pistaciae*	*Pistacia terebinthus*	October 2010
*Eupelmus * *pistaciae*	GDEL4027	10005	France	Hérault	Cazevieille	43.752222°	3.770000°	*Megastigmus pistaciae*	*Pistacia terebinthus*	October 2010
*Eupelmus * *pistaciae*	GDEL4027	10507	France	Hérault	Cazevieille	43.752222°	3.770000°	*Megastigmus pistaciae*	*Pistacia terebinthus*	October 2010
*Eupelmus * *priotoni*	GDEL4051	10038	France	Aveyron	Sauclières	43.96389°	3.355833°	Unknown	Unknown	June 2011
*Eupelmus * *purpuricollis*	LF.ur.GR 02	10650	Greece	Seres	nr Neo Petritsi	41°18’49,8”	23°16’35,6”	Unknown	Unknown	July 2008
*Eupelmus * *purpuricollis*	LF.ur.GR 03	10651	Greece	Seres	Kerkini	41°11’32,4”	23°03’59,5”	Unknown	Unknown	July 2007
*Eupelmus * *simizonus*	GDEL4142	10297	France	Ardèche	Les Vans	44.387222°	4.154444°	Unknown	On *Quercus pubescens*	July 2012
*Eupelmus * *tibicinis*	GDEL4148	10299	France	Ardèche	Chassagnes	44.403888°	4.178333°	Unknown	On *Quercus pubescens*	July 2012
*Eupelmus * *tibicinis*	GDEL4149	10300	France	Ardèche	Berrias-et-Casteljau	44.39389°	4.194722°	Unknown	Unknown	July 2012
*Eupelmus * *tibicinis*	GDEL4175	10598	France	Hérault	Laroque	45.91722°	3.74361°	Unknown	On *Quercus pubescens*	July 2013
*Eupelmus * *urozonus*	NB677	10333	France	Lot	Aynac	44.78155°	1.85896°	*Dryocosmus kuriphilus*	*Castanea sativa*	January 2012
*Eupelmus * *urozonus*	FAL1518	10410	France	Haute-Corse	Lumio	42.55879°	8.81299°	*Bactrocera oleae*	*Olea europaea*	September 2012
*Eupelmus * *urozonus*	FAL1060	10148	Italy	Liguria	Bussana-Vecchia	43.84026°	7.82905°	*Myopites stylata*	*Dittrichia viscosa*	January 2011
*Eupelmus * *urozonus*	L.Loru235	10241	Italy	Sardinia	Desulo	39.99198°	9.23053°	*Dryocosmus kuriphilus*	*Castanea sativa*	July 2011
*Eupelmus * *urozonus*	FAL1106	10165	Spain	Logroño	La Rioja	-	-	*Myopites stylata*	*Dittrichia viscosa*	March 2012
*Eupelmus * *urozonus*	NB1117	10251	Greece	Crete	Gournes	35.32822°	25.28388°	*Myopites stylata*	*Dittrichia viscosa*	March 2012
*Eupelmus * *urozonus*	MC-C100	10488	Switzerland	Riviera	Monte Ceneri	46.136944°	8.902500°	*Dryocosmus kuriphilus*	*Castanea sativa*	July 2012
*Eupelmus * *urozonus*	PJ10077-2-6	10573	Hungary	Vezprém	Várpalota	47.198091°	18.21204°	*Andricus lucidus*	*Quercus pubescens*/*Q. cerris*	November 2010
*Eupelmus * *urozonus*	LF.fu.RO 01	10464	Romania	Neamţ	Podoleni			Unknown	Unknown	September 2012
*Eupelmus * *urozonus*	LF.ur.IR 02	10457	Iran	Kerman	Bidkhan	-	-	Unknown	*Ephedra sp*.	March 2010
*Eupelmus * *vindex*	GDEL4054	10042	Hungary	Veszprém	Hegyesd	-	-	Unknown	Unknown	June 2010
*Eupelmus * *vindex*	LF.vi.RO 02	10468	Romania	Iaşi	Iaşi	-	-	Unknown	Unknown	June 2007
*Eupelmus * *vindex*	LF.vi.RO 01	10469	Romania	Tulcea	Letea	-	-	Unknown	Unknown	May 2007
*Eupelmus * *microzonus*	GDEL4116	10192	France	Haute-Corse	Aléria	42.128611°	9.465556°	*Bruchophagus* sp.	*Asphodelus ramosus*	September 2011
*Eupelmus * *atropurpureus*	PJ11159_23_1	10580	Spain	Aragón	Huesca			Unknown	Poaceae	November 2011
*Eupelmus * *pini*	GDEL4058	10048	France	Alpes-Maritimes	Guillaumes	44.070833°	6.853056°	Unknown	Dead trunk of *Pinus sylvestris*	August 2009
*Eupelmus * *matranus*	FAL1491	10318	France	Alpes-Maritimes	Sophia-Antipolis	43.61671°	7.07550°	Unknown	On *Quercus ilex*	October 2012
*Eupelmus * *falcatus*	GDEL4088	10090	Hungary	Veszprém	Nagavászony	47.021667°	17.724167°	Unknown	Unknown	June 2010
*Eupelmus * *seculatus*	GDEL4089	10091	France	Gard	Beauvoisin	43.712500°	4.307222°	Unknown	Unknown	August 2011
*Eupelmus * *linearis*	GDEL4069	10062	France	Lozère	Cocurès	45.30555°	4.59194°	Unknown	Unknown	July 2011
*Eupelmus * *linearis*	GDEL4073	10066	Hungary	Veszprém	Nagavászony	47.021667°	17.724167°	Unknown	Unknown	June 2010
*Eupelmus * *testaceiventris*	GDEL4078	10075	Cameroon	Adamaoua	Osséré Gadou	7.173056°	13.623056°	Unknown	Unknown	November 2008
*Eupelmus * *juniperinus thuriferae*	GDEL4064	10057	France	Hautes-Alpes	Saint-Crépin	44.710556°	6.606389°	Unknown	On *Juniperus thurifera*	August 2008
*Reikosiella* *aff. rostrata*	NB670	10336	France	Drôme	Génissieux	45.09059°	5.07161°	*Dryocosmus kuriphilus*	*Castanea sativa*	February 2012
*Reikosiella* *aff. rostrata*	NB810	10350	France	Alpes-Maritimes	Tende	44.056689°	7.579353°	*Dryocosmus kuriphilus*	*Castanea sativa*	March 2012
*Anastatus sidereu*s	GDEL4098	10105	France	Alpes-Maritimes	Fontan	44.026389°	7.577778°	Unknown	Unknown	July 2010
*Anastatus aff. temporalis*	GDEL4100	10107	France	Gard	Générac	43.719444°	4.353611°	Unknown	Unknown	August 2011

### Marker choice

Seven markers displaying various rates of molecular evolution were used: two coding portions of mitochondrial genes (Cytochrome oxidase I, *COI* and Cytochrome b, *Cytb*), two coding regions of nuclear genes (the F2 copy of elongation factor 1-alpha, *EF*-*1α* and *Wingless*, *Wg*) and three (at least partially) non-coding regions of other nuclear genes (the mitotic checkpoint control protein, *Bub3*; the ribosomal protein L27a, *RpL27a*, and the ribosomal protein S4, *RpS4*). All these markers were previously used for phylogenetic analyses in arthropods. *COI* and *Cytb* have been used to resolve insect molecular phylogenies at shallower taxonomic levels [38–41]. The *Wg* gene has provided a useful tool for the reconstruction of phylogenetic relationships at lower to intermediate taxonomic levels in different insect groups [32, 38, 41–45]. *EF*-*1α* has proven to evolve at slow rates and provide phylogenetic information at deeper levels (i.e. family relationships) [39, 46–51]. The *Bub3* gene is more rarely used [52, 53] for inferring phylogenetic relationships at a similar taxonomic level as *Wg*. Finally, ribosomal proteins *RpL27a* and *RpS4* have been used with success to infer the phylogeny of Hymenoptera associated with oak galls or figs [39, 54–56].

### DNA extraction, PCR amplification and sequencing

Genomic DNA was extracted from a single individual using the Qiagen DNeasy kit (Hilden, Germany) with some minor modifications with regard to the manufacturer’s protocol. Entire specimens were incubated at 56 °C for 15–17 h and DNA extraction was performed without destruction of the specimens, to allow subsequent examination of morphology (see § Sampling). Primer sequences are given in Additional file 1: Table S1.

For the two mitochondrial genes (*COI* and *Cytb*), the PCR mix was prepared in 20 μl as follows: 1 μl of DNA (1–55 ng/μl), 14.64 μl of Milli-Q water, 2 μl of 10x PCR buffer containing MgCl2 (1x), 1 μl of 10 μM primer cocktail (0.5 μM), 0.16 μl of dNTPs 25 mM each (0.2 mM) and 0.2 μl of 5 U/μl Taq DNA Polymerase (Qiagen, Hilden, Germany).

For the nuclear genes (*Bub3*, *EF1*-*α*, *RpL27a*, *RpS4* and *Wg*), the PCR mix was realised in 25 μl as follows: 2 μl of DNA (1–55 ng/μl), 19.825 μl of Milli-Q water, 2.5 μl of 10x PCR buffer containing MgCl2 (1x), 0.175 μl of 100 μM primer cocktail (0.7 μM), 0.2 μl of dNTPs 25 mM each (0.2 mM) and 0.125 μl of 5 U/μl Taq DNA Polymerase (Qiagen, Hilden, Germany).

PCR conditions for *Wg* and *COI* were as described in [32]. Those for other genes were as follows: *Cytb*: 94 °C for 5 min, followed by 40 cycles of (i) 94 °C for 1 min, (ii) 50 °C for 1 min, and (iii) 72 °C for 90 s with a final extension at 72 °C for 10 min; nuclear markers: 94 °C for 4 min, followed by 40 cycles of (i) 94 °C for 30 s, (ii) 58 °C for *EF*-*1α*, 48 °C for *Bub3*, 57 °C for *RpS4* and 55 °C for *RpL27a*, (iii) 72 °C for 5 min with final extension at 72 °C for 5 min.

In the absence of amplification or if the signal was too weak, we improved yields of PCRs by using 2x QIAGEN Multiplex PCR Master Mix (Qiagen, Hilden, Germany). In this case, PCRs were performed in a 25 μl reaction volume: 2 μl of DNA, 16.5 μl of Milli-Q water, 0.125 μl of 100 μM primer cocktail (0.5 μM) and 6.25 μl of 2x QIAGEN Multiplex PCR Master Mix (1x) and PCR conditions were as specified in the QIAGEN® Multiplex PCR kit: 95 °C for 15 min, followed by 40 cycles of (i) 95 °C for 30 s, (ii) 48 °C-58 °C for 90 s, (iii) 72 °C for 1 min, with final extension at 72 °C for 10 min.

All PCRs were performed on a GeneAmp 9700 thermocycler. PCR products were visualized using the QIAxcel Advanced System and QIAxcel DNA Fast Analysis Kit (Qiagen). PCR products were sent to GENOSCREEN (Lille, France) or to BECKMAN COULTER GENOMICS (Stansted, United Kingdom) for sequencing in both directions. All sequences were deposited in GenBank (Additional file 1: Table S2).

### Sequence alignment and phylogenetic analysis

#### Alignment

Sequences were aligned using Muscle [57] with the default settings as implemented in SeaView v4.4.1 [58] and subsequently visually checked. To assess the impact of indels on the phylogenetic resolution, highly divergent blocks present in *Bub3*, *RpS4* and *RpL27a* alignments were either included in or excluded from the analyses. These blocks were removed using Gblocks [59] with the default settings as implemented in SeaView. Alignments of *COI*, *Cytb*, *EF*-*1α* and *Wg* were translated to amino acids using Mega v5.1 [60] to detect potential frame-shift mutations and premature stop codons, which may indicate the presence of pseudogenes.

#### Gene by gene analysis

To detect (i) possible inconsistencies linked to contamination during laboratory procedures, (ii) poor-quality sequences, (iii) possible pseudogenes or other artefacts, and (iv) to evaluate the impact of the Gblock procedure on the individual phylogenetic resolution, genes were first analysed separately using a maximum likelihood approach (ML).

#### Concatenated datasets analysis

Phylogenetic analyses were performed on concatenated nucleotide sequences using both ML and Bayesian methods. Four partitioning schemes were compared: (i) two partitions: one for the two mitochondrial genes (*COI* and *Cytb*) and another for all nuclear markers (*Wg*, *EF*-*1α*, *Bub3*, *RpS4 & RpL27a*); (ii) six partitions: one for the two mitochondrial markers (*COI* and *Cytb*) and one for each nuclear marker (*Wg*, *EF*-*1α*, *Bub3*, *RpS4* and *RpL27a*); (iii) seven partitions: one for the 1st and 2nd codon positions of the mtDNA, one for the 3rd codon positions of mtDNA, and one for each nuclear gene (*Wg*, *EF*-*1α*, *Bub*, *RpS4* and *RpL27a*); (iv) nine partitions: same as above with *Wg* and *EF*-*1α* further partitioned by codon position (1st and 2nd codon positions *versus* 3rd positions).

Bayes factors (BF) [61, 62] were used to compare the four partitioning schemes. Harmonic means of the likelihood scores were used as estimators of the marginal likelihoods. Following [61] and [63], Bayes factors were calculated using the following formula: BF = 2 × (lnM1-lnM0) + (P1-P0) × ln (0.01) where lnMi and Pi are the harmonic-mean of the ln likelihoods and the number of free parameters of the model i, respectively. BF values were interpreted following [61] and [62], with BF values between 2 and 6, between 6 and 10 and higher than 10 indicating positive evidence, strong evidence, and very strong evidence favouring one model over the others respectively.

#### Evolution models and phylogenetic reconstruction

For the separated and concatenated datasets, the best-fitting model was identified using the Akaike information criterion (AIC) as implemented in jModelTest v0.1.1 [64].

For both gene-by-gene and concatenated analyses, maximum likelihood analyses and associated bootstrapping were performed using RAxML v8.0.9 [65]. The GTRCAT approximation of models was used for ML bootstrapping (1000 replicates). Bootstrap percentages (BP) ≥85 % were considered as strong support and BP < 65 % as weak.

Bayesian analyses were performed only on the concatenated dataset using a parallel version of MrBayes v3.2.2 [66]. Model parameters for each data partition were independently estimated by unlinking parameters across partitions. Parameter values for the model were initiated with default uniform priors, and branch lengths were approximated using default exponential priors. Bayesian inferences were estimated using two simultaneous, independent runs of Markov Chain Monte Carlo (MCMC), including three heated and one cold chains. The Metropolis-coupled MCMC algorithm [67] was used to improve the mixing of Markov chains. Analyses were run for 20 × 10^6^ generations with parameter values sampled every 2000 generations. To ensure convergence, 40 × 10^6^ generations were used for the most complex partitioning scheme (9 partitions) with parameter values sampled every 4000 generations. To increase and improve the swap frequencies of states between cold and heated chains, the heating temperature (T) was set to 0.01 for the most complex partitioning scheme cleaned with Gblocks and to 0.02 for all other datasets. Convergence was assessed using the standard deviation of split frequencies given by MrBayes and the Effective Sample Size (ESS), as estimated using Tracer v1.6.0 [68]. The first 25 % of the tree samples from the cold chain were discarded and considered as *burn*-*in*. Posterior probabilities (PP) ≥ 0.95 were considered as strong support and PP < 0.90 as weak.

Analyses were conducted using the CIPRES Science Gateway (www.phylo.org) [69].

#### Evolutionary properties of marker sequences

For each partition of the concatenated datasets (without Gblocks cleaning), base composition, substitution rates, and among sites rate variation (α) were estimated and compared. We also compared rate variation among partitions, considering the parameter m (rate multiplier).

### Comparative analysis

#### Evolution of ovipositor length

The ovipositor of Hymenoptera is a complex organ that exhibits great interspecific variation (see for instance [23]). In species of *Eupelmus*, part of the ovipositor is easily visible at the extremity of the abdomen (the ovipositor sheaths), while the rest is concealed in the abdomen. The use of this visible part as a “proxy” of the total ovipositor length is *a priori* tempting in order to avoid damaging of specimens of newly described species known from very few individuals [32, 33]. In order to validate the use of this proxy, a total of 34 individuals of comparatively common species (e.g. *E. azureus*, *E. confusus*, *E. gemellus*, *E. kiefferi*, *E. pistaciae*, and *E. urozonus*) were dissected and, for each individual, we measured the length of the ovipositor stylet, the visible part of the ovipositor sheath and the metatibia (see dataset on Dryad: doi:10.5061/dryad.115m1). Measurements of the length of the ovipositor sheaths and hind tibia followed Al khatib et al. [32] (Additional file 2: Figure S18 A and C). The length of the ovipositor stylet (first and second valvulae) was measured from the articulation of the second valvula with the articulating bulb to the apex of the second valvula (Additional file 2: Figure S18 B). Using this dataset, we found evidence of linear relationships between the ovipositor sheath (response variable) and either the ovipositor stylet or the metatibia as predictors (data not shown). Moreover, no interaction was found between these two predictors and the host species (respectively F_5df,20df_ = 1.23 with *p* = 0.34 and F_5df,22df_ = 1.20 with *p* = 0.34). This suggests that the visible part of the ovipositor sheath can indeed be used as a reliable proxy of the entire ovipositor.

As a consequence, a first analysis was performed on the 19 species of the “*E. urozonus* species group” for which information about the ovipositor sheaths and the metatibia were available. This analysis includes a total of 121 individuals, with at least 2 individuals/species except for *E. priotoni* and *E. simizonus* (only one individual in each case). In most of the cases, we tried to select individuals from at least two geographical locations and/or, for generalist species, two host species (see dataset on Dryad: doi:10.5061/dryad.115m1). Both the absolute length of the ovipositor sheath (“AOS”) and the ratio (“ROS”) between the ovipositor sheaths and the metatibia were taken into account, the second one being potentially less sensitive to environmental-induced phenotypic plasticity (host and/or abiotic conditions). AOS/ROS medians were then calculated for each *Eupelmus* species and these medians were used for the subsequent analysis (see below).

Two tests were then performed: (i) a Mantel test of the correlation between pairwise genetic distances (“phylogenetic matrix”) and pairwise differences in AOS/ROS (“morphological matrix”). (Dis) similarities were estimated as |d_i_-d_j_|/[(d_i_ + d_j_)/2] (d_i_ and d_j_ being the AOS/ROS medians obtained for species i and j respectively); (ii) the detection of a phylogenetic signal based on categories of AOS/ROS. For this purpose, “long ovipositors” (AOS/ROS exceeding the third quartile) were distinguished from “short ovipositors” (AOS/ROS below this threshold). Briefly, the sum of state changes was calculated, leading to a D statistic that could be tested against two theoretical distributions: a phylogenetic randomness and a Brownian distribution, this latter being underlain by a continuous trait evolving along the phylogeny at a constant rate [70].

#### Influence of phylogeny and ovipositor length on host range

A second analysis was restricted to a subset of 13 species for which host range was also available. Most of the information about host range was obtained from Al khatib et al. [32] and from Gibson and Fusu (in prep). Jean Lecomte (comm. pers.) communicated the rearing of *E. confusus* from curculionid larvae. Taken as a whole, our host survey is probably not exhaustive but nevertheless encompassed a total of several thousands of individuals of the “*E. urozonus* species group” and, with regard to the host’s diversity, 95 insect species representing 22 families and 6 orders (see dataset on Dryad: doi:10.5061/dryad.115m1). Taken as a whole, these host insects were distributed on 18 plant families. Dissimilarities in host range were calculated—at three taxonomic levels (species, family and order) for the host insect and at one level (family) for the host plant—using the Bray-Curtis distance, each host taxon being treated qualitatively (at least one record *versus* none). This information was summarized and presented as “ecological matrices”. Correlations between “phylogenetic”, “morphological” and “ecological” matrices were tested using simple (2 matrices) or partial (3 matrices) Mantel tests, the relevance of these last tests having been repeatedly discussed (see for instance [71] and [72]).

**Fig. 1:**
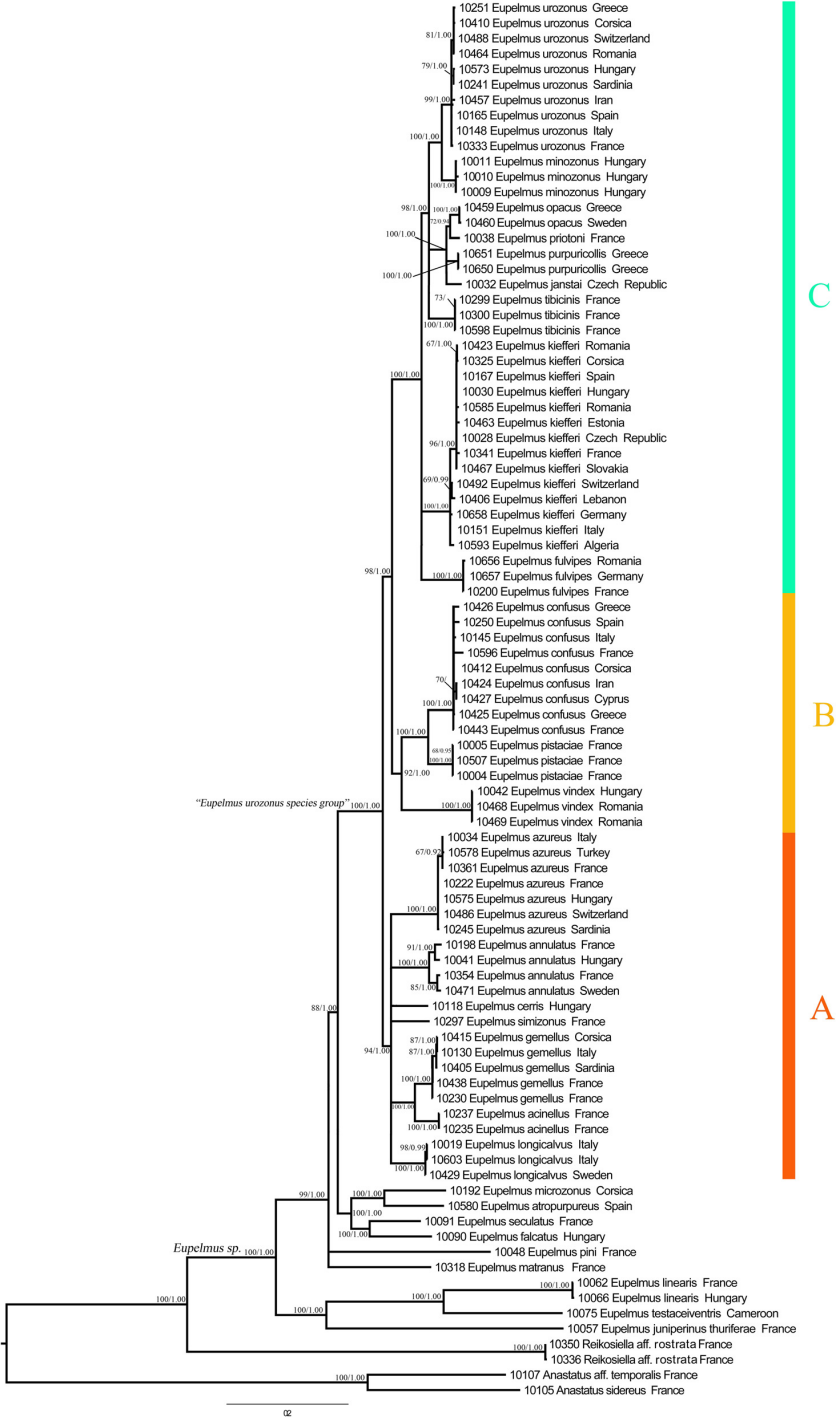
Phylogram of relationships among species of the “*Eupelmus urozonus* species group” obtained from the concatenated dataset alignment (5000 bp and 9 partitions) without the Gblocks cleaning of divergent blocks. Uppercase letters refer to clades discussed in the text. Nodes with likelihood bootstrap (BP) values <65 have been collapsed. BP (≥65) and Bayesian posterior probabilities (≥0.90) are indicated at nodes. Each line represents a sequenced individual with information in the following order: molecular code, species and country

Moreover, three kinds of traits were investigated using D-statistics (see previous paragraph):

- *Host specificity* (“specialists” which were reared from a single host species *versus* “generalists” that were reared from more than one host species). This specificity was evaluated at the order-family taxonomic level and at the species level. Because one may argue that our sampling underestimates specialists, we also performed this analysis under the assumption that all the rare species (*E. janstai*, *E. longicalvus*, *E. minozonus*, *E. priotoni*, *E. purpuricollis*, *E. vindex*) could be specialists.
- *Ability* (“Yes” or “No”) *to successfully parasitize some well-represented insect taxa at the ordinal level* (Coleoptera, Diptera, Hymenoptera and Lepidoptera) or at the family level (Cynipidae within Hymenoptera and Cecidomyiidae within Diptera).
- *The ability* (“Yes” or “No”) *to exploit some main host plants* (whatever the host insect), host plant being treated at the family level (Asteraceae, Fagaceae, Rosaceae, Salicaceae, etc.).

#### Software and packages

Manipulations of files and statistical tests were conducted using the software R (http://www.R-project.org - version 3.0.3 – 2014-03-06) with the following packages “ade4” (Euclidian transformation of matrices) [73], “ape” (phylogeny) [74], “caper” (comparative analysis), “ecodist” (Mantel tests) [75] and “vegan” (similarities between host ranges) [76].

## Results

### Alignments and single-marker analyses

Successful amplification and sequencing was completed for all gene regions used in this study. However, sequencing failures occurred for some markers for a few individuals. Genbank accessions of the sequences obtained for all analysed genes are given in Additional file 1: Table S2. The final matrix contained 91 specimens. No stop codons, frame shifts, insertions or deletions were observed in coding gene regions.

The numbers of aligned base pairs, variable sites and parsimony-informative sites for each gene are summarized in Table 2. As expected, mitochondrial genes showed more parsimony-informative sites compared to nuclear markers (472 out of 1085 bp). Among the nuclear markers, *EF*-*1α* exhibited the lowest number of variable and parsimony-informative sites (respectively 116 and 106 out of 517 bp). For *RpL27a*, removing the highly divergent alignment blocks significantly reduced the number of variable and parsimony-informative sites (from 54 to 38 % for variable sites and from 34 to 30 % for parsimony-informative sites). This loss consequently affected the resolution of the corresponding inferred topology (Additional file 2: Figure S16 and Figure S17). In contrast, the Gblocks procedure did not affect the number of variable and parsimony-informative sites for *Bub3* and *RpS4* and the resolution of the corresponding topologies (Additional file 2: Figures S12 – S15).

**Table 2:** Numbers and percentage of aligned base pairs, variable sites and parsimony-informative sites for the genes used in this study

Gene region	Total sites	Variable sites	Parsimony-informative sites
mtDNA	1085	530 (48.8 %)	472 (43.5 %)
*Wg*	433	157 (36.2 %)	147 (33.9 %)
*EF*-*1α*	517	116 (22.4 %)	106 (20.5 %)
*Bub3* alignment without Gblocks	481	161 (33.4 %)	140 (29.1 %)
*Bub3* alignment with Gblocks default	391	132 (33.7 %)	116 (29.7 %)
*RpS4* alignment without Gblocks	1259	451 (35.8 %)	323 (25.6 %)
*RpS4* alignment with Gblocks default	525	189 (36.0 %)	148 (28.1 %)
*RpL27a* alignment without Gblocks	1225	661 (53.9 %)	417 (34.0 %)
*RpL27a* alignment with Gblocks default	246	93 (37.8 %)	74 (30.0 %)

### Evolution models and partitions in the concatenated dataset

Alignment lengths of the concatenated datasets with or without the exclusion of highly divergent blocks were 3197 bp and 5000 bp respectively. For all partitions, the best-fitting substitution model was the general time reversible model (GTR) with among-sites rate variation (ASRV) modelled by a discrete gamma distribution (Γ) [77] for which we used four categories. For all Bayesian analyses, after discarding 25 % of the samples as *burn*-*in*, the ESS value of each parameter largely exceeded 200, which indicated that convergence of runs was reached. Sixteen combined trees were obtained (Additional file 2: Figures S1 – S8). For all combined datasets, Bayes factors showed that the most complex partitioning scenario (9 partitions) was preferred over the three less complex ones (Table 3).

**Table 3:** Partitioning strategy selecting using Bayes factors (Harmonic-Mean) in Bayesian analyses

Dataset partitioning models	Harmonic-mean (LnL)	Bayes factor
Alignments without Gblocks		
M1: mtDNA, nucDNA (2 partitions, 19 free parameters)	−38664.20	*M2*, *M1* = 907.0
M2: mtDNA, *Wg*, *EF*-*1α*, *Bub3*, *RpS4*, *RpL27a* (6 partitions, 59 parameters)	−38118.57	*M3*, *M1* = 1909.5
M3: mtDNA 1&2, mtDNA 3, *Wg*, *EF*-*1α*, *Bub3*, *RpS4*, *RpL27a* (7 partitions, 69 parameters)	−37594.33	*M3*, *M2* = 1002.4
M4: mtDNA 1&2, mtDNA 3, *Wg* 1&2, *Wg* 3, *EF*-*1α* 1&2, *EF*-*1α 3*, *Bub3*, *RpS4*, *RpL27a* (9 partitions, 89 parameters)	−37261.28	*M4*, *M1* = 2483.5
		*M4*, *M2* = 1576.43
		*M4*, *M3* = 574
Alignments with Gblocks default	Harmonic Mean (LnL)	Bayes factor
M1: mtDNA, nucDNA (2 partitions, 19 free parameters)	−27676.75	*M2*, *M1* = 150.1
M2: mtDNA, *Wg*, *EF*-*1α*, *Bub3*, *RpS4*, *RpL27a* (6 partitions, 59 parameters)	−27509.59	*M3*, *M1* = 1210.5
M3: mtDNA 1&2, mtDNA 3, *Wg*, *EF*-*1α*, *Bub3*, *RpS4*, *RpL27a* (7 partitions, 69 parameters)	−26956.35	*M3*, *M2* = 1060.4
M4: mtDNA 1&2, mtDNA 3, *Wg* 1&2, *Wg* 3, *EF*-*1α* 1&2, *EF*-*1α* 3, *Bub3*, *RpS4*, *RpL27a* (9 partitions, 89 parameters)	−26691.65	*M4*, *M1* = 1647.8
		*M4*, *M2* = 1497.73
		*M4*, *M3* = 437.3

### Evolutionary properties of the markers

Model parameter estimates for each partition of the Bayesian analysis of the “9 partitions without Gblocks cleaning dataset” are depicted in Table 4.

**Table 4:** Evolutionary properties of the partitions used in the study

Partitions	r (A↔C)	r (A↔G)	r (A↔T)	r (C↔G)	r (C↔T)	r (G↔T)
mtDNA 1&2	0.036 (0.015–0.059)	0.186 (0.134–0.241)	0.115 (0.089–0.141)	0.065 (0.034–0.099)	0.574 (0.503–0.642)	0.021 (0.010–0.034)
mtDNA 3	0.018 (0.006–0.029)	0.378 (0.310–0.445)	0.011 (0.00–0.014)	0.020 (0.00–0.048)	0.537 (0.464–0.608)	0.032 (0.021–0.046)
*Wg* 1&2	0.083 (0.021–0.149)	0.142 (0.057–0.240)	0.031 (0.000–0.079)	0.026 (0.000–0.064)	0.698 (0.565–0.827)	0.018 (0.000–0.056)
*Wg* 3	0.070 (0.042–0.100)	0.364 (0.274–0.459)	0.119 (0.072–0.171)	0.041 (0.024–0.058)	0.392 (0.300–0.484)	0.012 (0.000–0.029)
*EF*-*1α* 1&2	0.075 (0.–0.177)	0.070 (0.000–0.167)	0.040 (0–0.118)	0.182 (0.037–0.351)	0.197 (0.041–0.374)	0.432 (0.216–0.646)
*EF*-*1α* 3	0.052 (0.025–0.082)	0.481 (0.373–0.588)	0.072 (0.031–0.120)	0.018 (0.004–0.035)	0.342 (0.243–0.438)	0.031 (0.008–0.059)
*Bub*	0.084 (0.051–0.121)	0.289 (0.220–0.363)	0.069 (0.048–0.091)	0.036 (0.004–0.072)	0.456 (0.377–0.538)	0.062 (0.036–0.090)
*RpS4*	0.068 (0.047–0.090)	0.341 (0.296–0.388)	0.104 (0.085–0.123)	0.070 (0.042–0.099)	0.332 (0.288–0.378)	0.082 (0.062–0.104)
*RpL27a*	0.094 (0.070–0.119)	0.302 (0.257–0.348)	0.085 (0.070–0.101)	0.094 (0.054–0.138)	0.307 (0.260–0.353)	0.115 (0.089–0.141)
Partitions	pi A	pi C	pi G	pi T	α (Shape parameter)	m (Rtae multiplier)
mtDNA 1&2	0.271 (0.242–0.299)	0.137 (0.120–0.155)	0.147 (0.124–0.171)	0.443 (0.414–0.472)	0.133 (0.118–0.148)	0.580 (0.483–0.681)
mtDNA 3	0.418 (0.392–0.444)	0.049 (0.044–0.055)	0.051 (0.045–0.057)	0.480 (0.453–0.506)	0.635 (0.549–0.729)	8.929 (8.34–9.524)
*Wg* 1&2	0.284 (0.234–0.333)	0.215 (0.171–0.260)	0.288 (0.237–0.339)	0.211 (0.169–0.258)	0.076 (0–0.181)	0.034 (0.021–0.048)
*Wg* 3	0.151 (0.119–0.182)	0.402 (0.349–0.452)	0.278 (0.231–0.327)	0.168 (0.137–0.201)	1.086 (0.776–1.415)	1.254 (0.984–1.535)
*EF*-*1α* 1&2	0.307 (0.260–0.354)	0.212 (0.170–0.254)	0.258 (0.213–0.305)	0.222 (0.180–0.264)	0.093 (0–0.258)	0.029 (0.004–0.014)
*EF*-*1α* 3	0.178 (0.135–0.223)	0.373 (0.315–0.427)	0.176 (0.132–0.222)	0.270 (0.223–0.320)	0.769 (0.508–1.038)	0.336 (0.257–0.415)
*Bub*	0.351 (0.314–0.387)	0.129 (0.105–0.153)	0.169 (0.141–0.197)	0.349 (0.313–0.385)	0.222 (0.166–0.279)	0.190 (0.152–0.229)
*RpS4*	0.332 (0.308–0.354)	0.162 (0.146–0.180)	0.147 (0.131–0.163)	0.357 (0.334–0.380)	0.427 (0.364–0.496)	0.262 (0.224–0.303)
*RpL27a*	0.390 (0.367–0.412)	0.109 (0.096–0.123)	0.111 (0.097–0.124)	0.389 (0.366–0.410)	0.820 (0.693–0.946)	0.536 (0.455–0.619)

Mean and 95 % credibility intervals of the model parameters for each partition included in the Bayesian analyses of concatenated datasets without Gblocks cleaning (9 partitions) are reported

As expected, the mitochondrial partitions showed high base compositional bias (71.4 and 89.8 % of A/T for the first two positions and the third codon position respectively). Among the nuclear gene partitions, *RpL27a*, *Bub3* and *RpS4* were A/T-biased (77.9, 70 and 68.8 %) while the A/T percentage in the 3rd codon positions in *Wg* and *EF*-*1α* was only 32 and 45 % respectively.

With the exception of *EF*-*1α* 1st and 2nd codon positions (18.9 %), there was an overall higher rate of A-G and C-T transitions (from 60.8 % for *RpL27a* up to 91.6 % for mtDNA 3rd codon positions). More precisely, mtDNA (all codon positions), *Bub3* and *Wg* 1st & 2nd codon positions were in excess of C-T transitions.

For protein-coding genes (mtDNA, *EF*-*1α* and *Wg*), the rate multiplier parameter (m) was higher for the 3rd codon positions. Thus, mtDNA 3rd codon positions evolved more than sixteen times faster than the fastest nuclear gene (*RpL27a*).

The shape parameter of the gamma distribution (α) was also higher for the 3rd codon position of the protein coding genes, indicating that these positions show lower rate heterogeneity among sites. Additionally, α was lower for *Bub3* than for *RpS4* and *RpL27a*, indicating that *Bub3* had a greater rate of heterogeneity among sites.

### Phylogenetic trees inferred from concatenated datasets

#### Impacts of alignment strategy and reconstruction methods

ML and Bayesian topologies obtained from the concatenated alignments without Gblocks cleaning were more resolved than those obtained with removal of poorly aligned blocks. Whatever the partitioning scheme and regardless of whether or not divergent blocks were included in the analyses, most internal nodes were nevertheless statistically supported (BP value ≥ 65, PP value ≥ 90). Moreover, the 18 species recently defined by Al khatib et al. [32] and *E. vindex* were recovered as a monophyletic group.

**Fig. 2:**
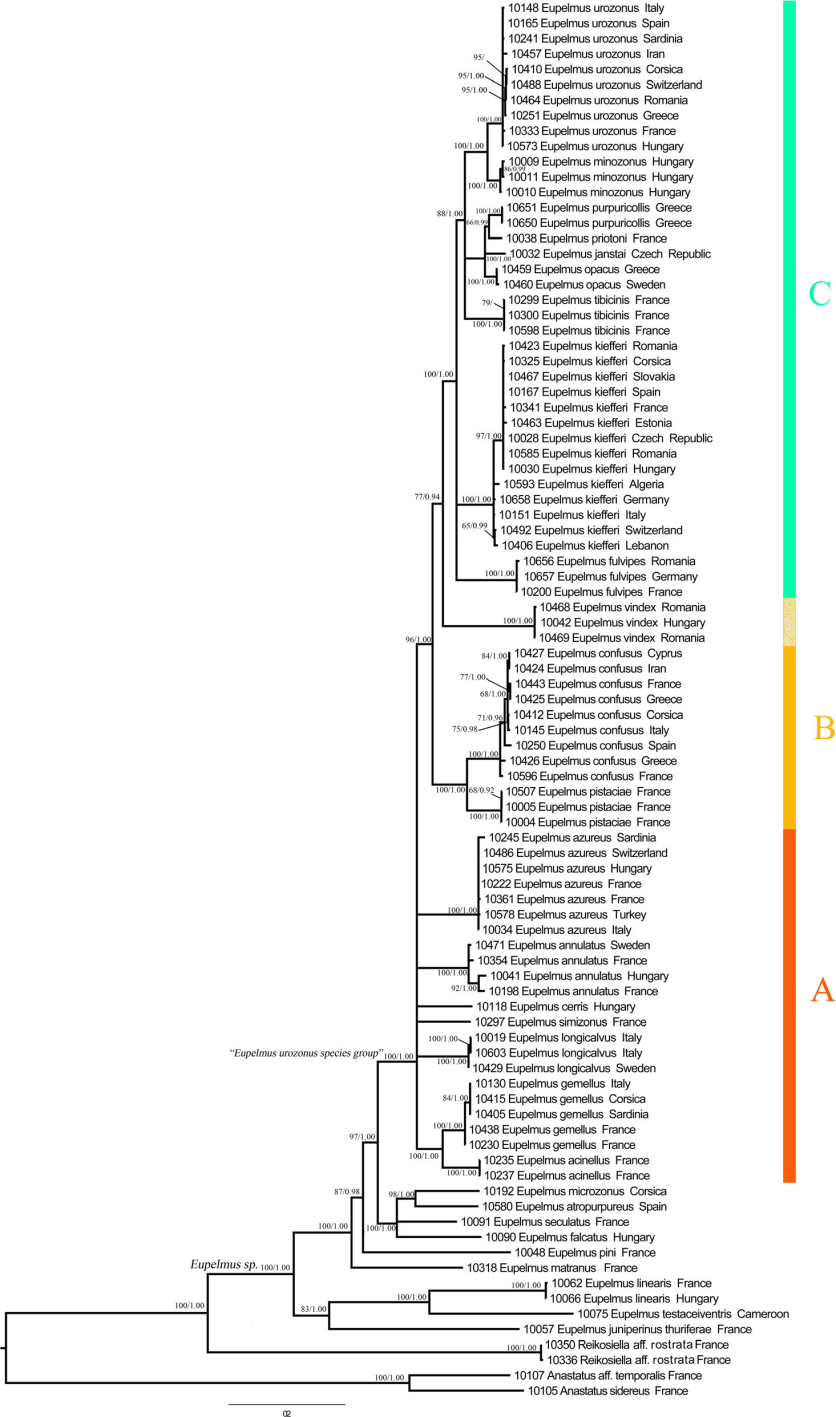
Phylogram of relationships among species of the “*Eupelmus urozonus* species group” obtained from the concatenated dataset alignment (3197 bp and 9 partitions) with Gblocks-default parameters. Uppercase letters refer to clades discussed in the text. Nodes with likelihood bootstrap (BP) values <65 have been collapsed. BP (≥65) and Bayesian posterior probabilities (≥0.90) are indicated at nodes. Each line represents a sequenced individual with information in the following order: molecular code, species, and country

Overall, topologies showed three major clades (A, B, C) that emerge on highly supported basal nodes (Figs. 1 and 2 and Additional file 2: Figures S1–S8). Three topological conflicts were observed depending on whether or not the Gblocks cleaning step was performed: (i) Clade A was not supported in topologies inferred from the datasets cleaned using Gblocks (Fig. 2 and Additional file 2: Figures S5–S8); (ii) *E. vindex* was sister to the rest of clade C in the topologies inferred from data sets cleaned using Gblocks (Fig. 2 and Additional file 2: Figures S5–S8), while it was sister to *E. confusus* and *E. pistaciae* (clade B) without Gblocks cleaning (Fig. 1 and Additional file 2: Figures S1–S4); (iii) the relationships of *E. matranus* and *E. pini* were resolved when Gblocks was used (PP = 1 and 0.98 respectively) (Fig. 2 and Additional file 2: Figures S5–S8), but not resolved without Gblocks cleaning of data sets (Fig. 1 and Additional file 2: Figures S1–S4). Taken as a whole, we decided to favour the alignment without the Gblocks procedure for the comparative analysis in order to favour the resolution for the terminal nodes.

**Fig. 3:**
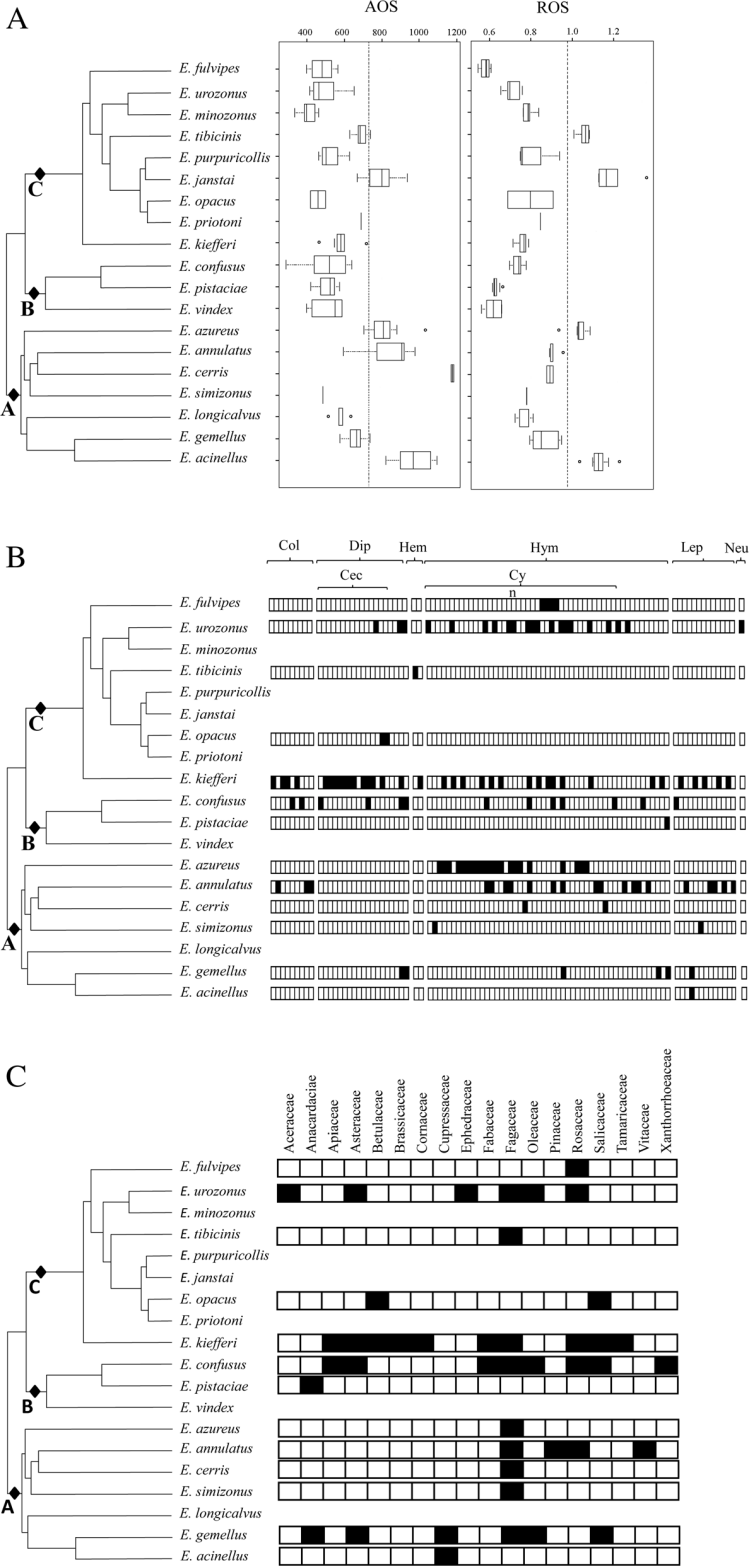
Mapping of ovipositor size and host ranges (host insect and related plants) along the multi-locus phylogeny of the “*Eupelmus urozonus* species group”. The phylogenetic tree used is derived from the Fig. 1. For convenience, sizes of branches were modified but the topology remains unchanged. In Fig. 3a, boxplots are shown for the absolute (AOS in μm) and relative (ROS – no unit) lengths of the ovipositor for each *Eupelmus* species. In each case, the vertical dotted line separates “short” versus “long” ovipositors. In Fig. 3b, the host specificity is indicated at three levels (from up to down): order, family, and species. Each rectangle indicates a possible host and the black ones indicate that at least one *Eupelmus* specimen was obtained from this host. In Fig. 3c, the plant host is indicated at the family level

#### Molecular relationships within the “*Eupelmus urozonus* species group”

ML and Bayesian analyses performed on the most complex partitioning scheme without Gblocks cleaning produced similar topologies with only a few differences for poorly supported nodes (Additional file 2: Figure S1). We therefore mapped all node support values (BP & PP) on the ML topology (Fig. 1).

In all analyses, the “*E. urozonus* species group” was recovered as monophyletic (Fig. 1) with a strong support. The group was subdivided into three clades, “clades” being defined here as a statistically-supported basal divergence including several species:

- Clade A included *E. acinellus*, *E. annulatus*, *E. azureus*, *E. cerris*, *E. gemellus*, *E. longicalvus* and *E. simizonus*, whose relative positions were not resolved to the exception of the sister species relationship between *E. acinellus* and *E. gemellus* (BP = 100, PP = 1).
- Clade B included three species with *E. vindex* being sister to *E. confusus* plus *E. pistaciae* with strong support (BP = 92, PP = 1).
- Clade C included the remaining species and namely *E. fulvipes*, *E. janstai*, *E. kiefferi*, *E. minozonus*, *E. opacus*, *E. priotoni*, *E. purpuricollis*, *E. tibicinis* and *E. urozonus*. Within clade C, two well-supported (in each case, BP = 100, PP = 1) subclades—“sub-clade” being defined as a more terminal divergence including at least 2 species—can be distinguished (i) *E. opacus*, *E. priotoni*, *E. purpuricollis* and *E. janstai*; (ii) *E. minozonus* and *E. urozonus*. These two subclades together with *E. tibicinis*, whose exact phylogenetic position remains unclear, form a well-supported monophyletic group (BP = 98, PP = 1).

### Comparative analysis and host uses

There were significant interspecific differences for both the absolute (AOS—Kruskal-Wallis test: χ^2^_16df_ = 93.7; *p* < 10^−3^; *E. priotoni* and *E. simizonus* discarded because of lack of replicates) and relative (ROS—Kruskal-Wallis test: χ^2^_16df_ = 109.2; *p* < 10^−3^; *E. priotoni* and *E. simizonus* also discarded) ovipositor lengths (Fig. 3a). AOS ranged from 398 μm in *E. minozonus* to a maximum of 1179 μm in *E. cerris* while ROS ranged from a minimum of 0.58 in *E. fulvipes* to a maximum of 1.16 in *E. janstai*. Even if AOS and ROS medians were significantly correlated one with another (Kendall’s rank correlation: *z* = 2.73; *p* = 0.006), some discrepancies were observed as for *E cerris* which exhibits the highest AOS but an intermediate ROS (Fig. 3a).

Within the “*Eupelmus urozonus* species group”, there was no significant correlation between similarity in ovipositor length and phylogenetic distance (Mantel test for AOS: *r* = 0.09, *p* = 0.39 – Mantel test for ROS: *r* = 0.08, *p* = 0.44). When ovipositor length was treated as a binary variable with “long” ovipositors being those above the third quartile (4 or 5 cases among the 19 species), the observed D-statistics for AOS (0.13) and ROS (1.33) never departed from a random distribution (respectively *p* =0.13 and *p* =0.61) or a Brownian one (respectively *p* =0.48 and *p* =0.14). Consequently, it seems that no strong clustering existed on the length of the ovipositor sheaths. Remarkable differences in the length of the ovipositor sheaths were even observed between some sister species: *E. acinellus*—*E. gemellus* in clade A and *E. janstai*—*E. purpuricollis* in clade B (Fig. 3a).

Taken as a whole, our results indicated that both Cynipidae and Cecidomyiidae constitute the main host species for West Palearctic "*E. urozonus* species group" (Fig. 3b). Yet, contrasted feeding regimes (specialists versus generalists) were observed (Fig. 3b). Only three (*E. acinellus*, *E. pistaciae* and *E. tibicinis*) of the 13 species are strict specialists, with a distribution (*D* = 2.38) not significantly departing from both a random (*p* = 0.79) or a Brownian distribution (*p* =0.11). At the family and order level (same distributions), three other species were specialists of Cynipidae—*E. azureus* (reported on 21 host species), *E. cerris* (2 hosts) and *E. fulvipes* (4 hosts)—and one (*E. opacus*) on Cecidomyiidae. At these levels, the relative distribution of specialists and generalists (*D* = 1.65) does not differs from a random (*p* =0.72) or Brownian distribution (*p* =0.10) and, as shown in Fig. 33b, about 50–60 % of the described species in each of the three clades were specialists. The absence of a phylogenetic signal still holds under the assumption that all rare species (*E. janstai*, *E. longicalvus*, *E. minozonus*, *E. priotoni*, *E. purpuricollis*, *E. vindex*) are specialists. Departures from a random distribution is never significant (host species’ level: *D* =1.04 with *p* =0.51 – host order’s level: *D* =1.52 with *p* =0.76) while a significant departure is observed from a Brownian distribution at the host order’s level (host species’ level: *p* =0.12 – host order’s level: *p* =0.031). Interestingly, contrasted host ranges were observed between sister species: *E. gemellus* (six host species distributed in 3 orders)—*E. acinellus* (one host species) within clade A and *E. confusus* (thirteen species distributed in four orders)—*E. pistaciae* (one host species) within clade B (Fig. 3b).

We investigated the ability of the “*E. urozonus* species group” to parasitize host species belonging to Coleoptera, Diptera, Hymenoptera and Lepidoptera (ordinal level) or Cecidomyiidae within Diptera and Cynipidae within Hymenoptera (familial level) (see Fig. 3b). However, in all these cases, we were not able to observe significant departures from a random or a Brownian distribution (See Additional file 3: Table S4).

Correlations between phylogenetic, morphometric (absolute or relative lengths of the ovipositor sheaths, AOS and ROS) and ecological (host ranges) matrices were also tested using simple or partial Mantel tests, at each of the three levels (species, family and order). Overall, the Mantel coefficients ranged between −0.07 and +0.14 and were never significantly different from zero (see Additional file 4: Table S3). At the host species level, such a result could be explained by the fact that only 24 % of the hosts (mostly Cynipidae) are shared by at least two species of the “*E. urozonus* species group”. As a consequence, this level of investigation may be too precise to detect any signal. However, such a limit cannot be taken into account at the two other taxonomic levels since about half of the host families and all host orders except Neuroptera are shared by at least two species of *Eupelmus*. Taken as a whole, these results confirm those obtained using D-statistics about the absence of significant phylogenetic constraints on the host range evolution. The relative ovipositor length also does not appear to be a significant driver of the host use.

When host plants rather than host insects are taken into account, 18 plant families were identified (see Fig. 3c), eight of which being used by only one *Eupelmus* species. However, four main families were used by at least four *Eupelmus* species: Asteraceae (4 species), Fagaceae (9 species), Rosaceae (5 species) and Salicaceae (4 species). For each of these families, no phylogenetic signal was detected using the D-statistics (See Additional file 3: Table S4). Additionally, no correlation was found between the related ecological matrix and the phylogenetic, and/or morphometric (AOS/ROS) matrices (see Additional file 5: Table S5).

## Discussion

### Phylogenetic relationships within the “*E. urozonus* species group”

Phylogenetic inter-specific relationships within the “*E. urozonus* species group” occurring in the Palaearctic region were recently investigated by Al khatib et al. [32] based on morphological characters and two genetic markers (mitochondrial *COI* and nuclear *Wg*). This study showed an unsuspected diversity but it (i) failed to resolve phylogenetic relationships at both deep and intermediate levels, (ii) highlighted some discrepancies among tree topologies at the shallowest nodes resulting from *COI* and *Wg* sequences, (iii) did not include morphologically divergent but potentially phylogenetically closely related species. By considering new species and adding more informative markers, the present study improved the knowledge on the evolutionary history of the “*E. urozonus* species group”.

Although the phylogenetic resolution was proven to be sensitive to inclusion or exclusion of divergent blocks by using Gblocks procedure from the sequence alignments, we obtained a reliable phylogeny which strongly supported the monophyly of our focus group of *Eupelmus*, including the 18 species treated in Al khatib et al. [32] and *E. vindex*, which is morphologically distinct from other members of the group in the shape of the syntergum and the anterior displacement of the ovipositor sheaths (Gibson & Fusu, in prep). Additionally, the included species of the “*E. urozonus* species group” were distributed in three strongly supported clades, referred here as A, B and C (Fig. 1).

The molecular monophyly of the Palaearctic “*E. urozonus* species group” reflected in our concatenated datasets can be also supported through morphology. Al khatib et al. (in prep.) recently compared and combined the results of phylogenetic inferences using the molecular data presented here with morphological data. The main conclusion of this complementary work seems to be the structuration of *Eupelmus* as a set of independent species groups (including our focus group). Their delineation and their morphological supports are therefore not detailed here.

Despite using several loci from both the nuclear and mitochondrial genomes, some of the focal taxa remain poorly resolved. We expect that newer methods that dramatically increase the number of loci will help to better resolve these relationships (see for instance [78]).

### Ecological differentiation within the “*E. urozonus* species group”

The diversification of parasitic organisms has been explained by various processes linking ecological specialization and speciation. For parasitoids, phylogenetic information and reliable host ranges are necessary to describe the patterns (distribution of generalist and specialist species) and to understand the underlying processes (e.g. “musical chairs” *versus* “oscillation”). This motivated the present work. Although members of the genus *Eupelmus* are usually described as generalist ectoparasitoids [27, 28], our study nevertheless leads to a more complex pattern. Our results indeed showed the coexistence of “strict” specialists restricted to one specific host (i.e. *E acinellus*, *E. pistaciae*, *E. tibicinis*), intermediate specialists that can parasitize various species of Cynipidae (i.e. *E. azureus*, *E. cerris* and *E. fulvipes*) and generalists that are able to successfully develop on different insect orders (i.e. *E. annulatus*, *E. confusus*, *E. gemellus* and *E. kiefferi*).

This diversity in host use observed in the “*E. urozonus* species group” does not seem to be driven by phylogenetic history as generalists and specialists were recovered in each of the three clades. Moreover, some sister species exhibited fully contrasted ecologies (generalist species cited first): *E. confusus*—*E. pistaciae* and *E. gemellus*—*E. acinellus*. In this last case, because the facultative hyperparasitism lifestyle is recorded for some species of *Eupelmus*, we strongly suspect that *E. gemellus* develops as a hyperparasitoid of *E. acinellus* on *Mesophleps oxycedrella* (Lepidoptera). If this is true, it would mean that none of these generalists (*E. confusus* and *E. gemellus*) share any hosts with its sister species. Even if it is not the case, such contrasting patterns of host use remain, to our knowledge, rare in parasitoid species.

Quite similar conclusions arose when host plants instead hosts insects were taken into account. There was indeed no correlation between host plant ranges, phylogenetic and/or morphometric constraints. Moreover, the use of the four main plant families (Asteraceae, Fagaceae, Rosaceae and Salicaceae) did not seem to be constrained by the phylogenetic history. The underlying rationale of this complementary analysis was that host plants could at least partly determine ecological specialization of *Eupelmus* species insofar as the parasitoid species could use, innately or through learning, plant-linked cues in order to locate favourable environments, be the cues emitted passively (olfactory or visual information) or actively (synomones) (see for instance [79–81]). One criticism to this approach would, of course, be the level (plant family) at which our analysis was performed since it implies that only well-conserved cues could be detected.

A final facet of our investigation was the potential role of the ovipositor sheaths (as a proxy of the ovipositor length) as a driver of host use. The rationale was that (i) ovipositor structure could be constrained by the phylogenetic history of the species and, (ii) ovipositor length could determine accessibility to different hosts [82, 83]. None of these hypotheses was however verified, ovipositor length appearing to be a very labile trait within our focus group.

Another driver of host range evolution could be the complexity of gall communities exploited by the *Eupelmus* species. Indeed, in numerous cases, *Eupelmus* species are occurring with numerous parasitoid species belonging to different chalcid families (*e.g*. Torymidae, Eurytomidae or Pteromalidae) which seem to be more functionally adapted to their hosts (see for instance [34, 84] and [85]). Such recurrent interspecific competitions may represent a potential limit for the abundance of *Eupelmus* but may also, ultimately, offer evolutionary opportunities. In particular, such an ecological intimacy could promote some switches towards unusual but ecologically related host insects and/or transitions towards other developmental modes (hyperparasitism or even predation). Such kind of adaptations may be illustrated by *E. tibicinis*, a specialist predator of the eggs of the red cicada, *Tibicina haematodes* (Scopoli, 1763) (Hemiptera: Tibicinidae).

## Conclusions

This paper provides comprehensive information about the ecological differentiation within the Palaearctic species of the “*E. urozonus* species group” and contributes to our understanding of ecological specialization in parasitoids. Although further investigations are required, the intimate mixing of generalist and specialist species along the phylogeny leans toward the “oscillation hypothesis” (*sensu* Hardy and Otto [21]). It also raises new questions at both the inter- and intra-specific levels. At the intra-specific level, more detailed population genetics studies would be useful to test the existence of “host races” within generalist species, which could be a way to, (i) explain the capacity of a single species to develop in different hosts and (ii) offer opportunities for the recurrent apparition of specialized lineages and ultimately species. At the interspecific level, the partitioning of the available resources within sympatric *Eupelmus* species and with other chalcid wasps remains unclear. This would probably require a better knowledge of potential and realised host ranges, interspecific interactions (e.g., competition and hyperparasitism) and investigations on the influence of host plants on the associated parasitoids (e.g., attraction/repellence; phenology and structure of galls). Finally, an agronomic output of such investigations would be a better knowledge of the actual potential of some *Eupelmus* species to regulate certain insect pests such as the olive fruit fly, *Bactrocera oleae* (Gmelin, 1790) [86–89] or the chestnut gall wasp *Dryocosmus kuriphilus* Yasumatsu, 1951 [90–92].

### Availability of supporting data

The data sets supporting the results are available in Dryad (doi: 10.5061/dryad.115m1).

All sequences are available in Genbank (http://www.ncbi.nlm.nih.gov/genbank). Genbank accession numbers are given in Additional file 2: Table S2.

## Additional files

Additional file 1: Table S1. Primer sequences used in the study and related references. **Table S2.** Information (including identification codes, taxonomic identity and Genbank accession numbers) related the specimens used in the phylogenetic analyses. (DOCX 50 kb) Additional file 2: Figure S1. Trees from a) the ML and b) Bayesian analyses of the combined dataset (without Gblocks cleaning, 9 partitions). Likelihood bootstrap values and posterior probabilities are indicated at nodes. **Figure S2.** Trees from a) the ML and b) Bayesian analyses of the combined dataset (without Gblocks cleaning, 7 partitions). Likelihood bootstrap values and posterior probabilities are indicated at nodes. **Figure S3.** Trees from a) the ML and b) Bayesian analyses of the combined dataset (without Gblocks cleaning, 6 partitions). Likelihood bootstrap values and posterior probabilities are indicated at nodes. **Figure S4.** Trees from a) the ML and b) Bayesian analyses of the combined dataset (without Gblocks cleaning, 2 partitions). Likelihood bootstrap values and posterior probabilities are indicated at nodes. **Figure S5.** Trees from a) the ML and b) Bayesian analyses of the combined dataset (with Gblocks-default parameters, 9 partitions). Likelihood bootstrap values and posterior probabilities are indicated at nodes. **Figure S6.** Trees from a) the ML and b) Bayesian analyses of the combined dataset (with Gblocks-default parameters, 7 partitions). Likelihood bootstrap values and posterior probabilities are indicated at nodes. **Figure S7.** Trees from a) the ML and b) Bayesian analyses of the combined dataset (with Gblocks-default parameters, 6 partitions). Likelihood bootstrap values and posterior probabilities are indicated at nodes. **Figure S8.** Trees from a) the ML and b) Bayesian analyses of the combined dataset (with Gblocks-default parameters, 2 partitions). Likelihood bootstrap values and posterior probabilities are indicated at nodes. **Figure S9.** Tree from the ML analysis of the mitochondrial partition. Likelihood bootstrap values (1000 replicates) and posterior probabilities are indicated at nodes. **Figure S10.** Tree from the ML analysis of the *Wg* locus. Likelihood bootstrap values (1000 replicates) and posterior probabilities are indicated at nodes. **Figure S11.** Tree from the ML analysis of the *EF*-*1α* locus. Likelihood bootstrap values (1000 replicates) and posterior probabilities are indicated at nodes. **Figure S12.** Tree from the ML analysis of the *Bub3* locus (without Gblocks cleaning). Likelihood bootstrap values (1000 replicates) and posterior probabilities are indicated at nodes. **Figure S13.** Tree from the ML analysis of the *Bub3* locus (with Gblocks-default parameters). Likelihood bootstrap values (1000 replicates) and posterior probabilities are indicated at nodes. **Figure S14.** Tree from the ML analysis of the *RpS4* locus (without Gblocks cleaning). Likelihood bootstrap values (1000 replicates) and posterior probabilities are indicated at nodes. **Figure S15.** Tree from the ML analysis of the *RpS4* locus (with Gblocks-default parameters). Likelihood bootstrap values (1000 replicates) and posterior probabilities are indicated at nodes. **Figure S16.** Tree from the ML analysis of the *RpL27a* locus (without Gblocks cleaning). Likelihood bootstrap values (1000 replicates) and posterior probabilities are indicated at nodes. **Figure S17.** Tree from the ML analysis of the *RpL27a* locus (with Gblocks-default parameters). Likelihood bootstrap values (1000 replicates) and posterior probabilities are indicated at nodes. **Figure S18.** Illustrations of morphometric measurements on *Eupelmus* females. (A) ovipositor sheaths, (B) ovipositor stylet (second and third pairs of valvulae), and (C) hind tibia. (PDF 110496 kb) Additional file 3: Table S4. Summary of information related to the detection of a phylogenetic signal (both host insects and plants). (DOCX 20 kb) Additional file 4: Table S3. Summary of Mantel tests used for the comparative analysis dealing with host insects. (DOCX 21 kb) Additional file 5: Table S5. Summary of Mantel tests used for the comparative analysis dealing with host plants. (DOCX 19 kb)
