# Supplementary material for: Multilocus phylogeny and ecological differentiation of the “Eupelmus urozonus species group” (Hymenoptera, Eupelmidae) in the West-Palaearctic
Source: BMC Evol Biol. 2016 Jan 19;16:13. doi: 10.1186/s12862-015-0571-2 (PMC4717567; doi:10.1186/s12862-015-0571-2)
Supplement: Additional file 1: Table S1. — Primer sequences used in the study and related references. Table S2. Information (including identification codes, taxonomic identity and Genbank accession numbers) related the specimens used in the phylogenetic analyses. (DOCX 50 kb) [file 12862_2015_571_MOESM1_ESM.docx]

**Additional file 1: Table S1 Primer sequences used in the study and related references.**

| **Locus** | **Primer** | **F/R** | **Primer sequences** | **Primer sources** |
| --- | --- | --- | --- | --- |
| ***COI*** | LCO1490 | F | GGTCAACAAATCATAAAGATATTGG | Folmer *et al.* (1994) |
|  | HCO2198 | R | TAAACTTCAGGGTGACCAAAAAATCA |  |
| ***Cyt b*** | CB1 | F | TATGTACTACCATGACGACAAATATC | Jermiin & Crozier (1994) |
|  | CB2 | R | ATTACACCTCCTAATTTATTAGGAAT |  |
| ***Wg*** | wg1a | F | GARTGYAARTGYCAYGGYATGTCTGG | Brower & DeSalle (1998) |
|  | LepWg2 | R | ACTNCGCRCACCARTGGAATGTRCA | Danforth *et al.* (2004) |
| ***EF-1α*** | F2-557F | F | GAACGTGAACGTGGTTATYACSAT | Cruaud *et al.* (2011) |
|  | F2-1118R | R | TTACCTGAAGGGGAAGACGRAG |  |
| ***Bub3*** | Bub3F2 | F | GATCCYAGAACACCYACYTGTGTWGG | Kawakita *et al*. (2008) |
|  | Bub3Rev1 | R | CGYTTYTTRTTRAARCCATCCC |  |
| ***RpS4*** | 11F | F | BAARGCATGGATGTTRGACA | Lohse *et al.* (2011) |
|  | 11R | R | GGTCWGGRTADCGRATRGT |  |
| ***RpL27a*** | 28Fb | F | CAAYTTYGACAARTACCATCCWG | Lohse *et al.* (2011) |
|  | 28R | R | CCYTTKCCYARRAGTTTGTA |  |

**Additional file 2: Table S2 Information (including identification codes, taxonomic identity and Genbank accession numbers) related the specimens used in the phylogenetic analyses.**

| **Collection code** | **Molecular code** | **Species** | ***COI*** | ***Cyt b*** | ***Wg*** | ***EF-1α*** | ***Bub3*** | ***RpS4*** | ***RpL27a*** | **Voucher depository** |
| --- | --- | --- | --- | --- | --- | --- | --- | --- | --- | --- |
| GDEL4100 | 10107 | *Anastatus aff. temporalis* | KR348752 | KR348858 | KR708338 | KR708449 | KR905352 | KR905274 | KR708469 | GDPC |
| GDEL4098 | 10105 | *Anastatus sidereu*s | KR348751 | KR348859 | KR708337 | KR708448 | KR905351 | KR905273 | KR708468 | GDPC |
| GDEL4064 | 10057 | *Eupelmus juniperinus thuriferae* | KR348746 | KR348857 | KR708332 | KR708445 | KR905362 | KR905275 | KR708463 | GDPC |
| GDEL4069 | 10062 | *Eupelmus linearis* | KJ018334 | KR348854 | KJ018530 | KR708442 | KR905359 | KR905268 | KR708464 | GDPC |
| GDEL4073 | 10066 | *Eupelmus linearis* | KR348747 | KR348855 | KR708333 | KR708443 | KR905360 | KR905269 | KR708465 | GDPC |
| GDEL4078 | 10075 | *Eupelmus testaceiventris* | KR348748 | KR348856 | KR708334 | KR708444 | KR905361 | KR905270 | _ | GDPC |
| FAL1363 | 10235 | *Eupelmus* *acinellus* | KJ018383 | KR348783 | KJ018562 | KR708370 | KR905287 | KR905199 | KR708481 | FALPC |
| FAL1366 | 10237 | *Eupelmus* *acinellus* | KJ018384 | KR348784 | KJ018563 | KR708371 | KR905288 | KR905200 | KR708482 | FALPC |
| GDEL4053 | 10041 | *Eupelmus annulatus* | KJ018333 | KR348774 | KJ018529 | KR708361 | KR905277 | KR905190 | KR708460 | GDPC |
| FAL1176 | 10198 | *Eupelmus annulatus* | KJ018363 | KR348772 | KJ018547 | KR708359 | KR905276 | KR905188 | KR708477 | FALPC |
| NB783 | 10354 | *Eupelmus annulatus* | KJ018403 | KR348773 | KJ018579 | KR708360 | KR905278 | KR905189 | KR708496 | FALPC |
| LF.an.SW 01 | 10471 | *Eupelmus annulatus* | KJ018439 | KR348775 | KJ018601 | KR708362 | KR905279 | KR905191 | KR708516 | AICF |
| PJ11159_23_1 | 10580 | *Eupelmus atropurpureus* | KR348771 | KR348850 | KR708358 | KR708438 | KR905355 | KR905267 | KR708523 | FALPC |
| GDEL4048 | 10034 | *Eupelmus azureus* | KJ018331 | KR348778 | KJ018527 | KR708365 | KR905282 | KR905194 | KR708458 | GDPC |
| FAL1323 | 10222 | *Eupelmus azureus* | KR348755 | KR348776 | KR708341 | KR708363 | KR905280 | KR905192 | KR708479 | CBGP |
| L.Loru713 | 10245 | *Eupelmus azureus* | KR348757 | KR348779 | KR708343 | KR708366 | KR905283 | KR905195 | KR708484 | MNHN |
| NB773a | 10361 | *Eupelmus azureus* | KJ018404 | KR348777 | KJ018580 | KR708364 | KR905281 | KR905193 | KR708497 | FALPC |
| MC-C4 | 10486 | *Eupelmus azureus* | KR348769 | KR348782 | KR708356 | KR708369 | KR905286 | KR905198 | _ | CBGP |
| PJ10077-21-4 | 10575 | *Eupelmus azureus* | KJ018448 | KR348780 | KJ018606 | KR708367 | KR905284 | KR905196 | KR708521 | MNHN |
| PJ11054-2-2 | 10578 | *Eupelmus azureus* | KJ018449 | KR348781 | KJ018607 | KR708368 | KR905285 | KR905197 | KR708522 | FALPC |
| GDEL4109 | 10118 | *Eupelmus* *cerris* | KJ018335 | _ | KJ018531 | KR708372 | KR905289 | KR905201 | KR708470 | GDPC |
| FAL1051 | 10145 | *Eupelmus confusus* | KJ018345 | KR348795 | KJ018535 | KR708383 | KR905299 | KR905214 | KR708471 | GDPC |
| FAL1108 | 10250 | *Eupelmus confusus* | KR348758 | KR348796 | KR708344 | KR708384 | KR905304 | KR905211 | KR708485 | GDPC |
| FAL1519 | 10412 | *Eupelmus confusus* | KJ018409 | KR348794 | KJ018582 | KR708382 | KR905298 | KR905210 | KR708501 | MNHN |
| LF.ma.IR 05 | 10424 | *Eupelmus confusus* | KJ018474 | KR348800 | KJ018624 | KR708388 | _ | KR905215 | KR708504 | AICF |
| LF.ma.GR 01 | 10425 | *Eupelmus confusus* | KJ018416 | KR348797 | KJ018586 | KR708385 | KR905300 | KR905216 | KR708505 | AICF |
| LF.ma.GR 02 | 10426 | *Eupelmus confusus* | KJ018417 | KR348798 | KJ018587 | KR708386 | KR905301 | KR905212 | KR708506 | AICF |
| LF.ma.CY 01 | 10427 | *Eupelmus confusus* | KJ018473 | KR348801 | KJ018625 | KR708389 | KR905303 | _ | KR708507 | AICF |
| FAL1278 | 10443 | *Eupelmus confusus* | KJ018424 | KR348793 | KJ018592 | KR708381 | KR905305 | KR905213 | KR708510 | MNHN |
| GDEL4173 | 10596 | *Eupelmus confusus* | KJ018452 | KR348799 | KJ018608 | KR708387 | KR905302 | KR905217 | KR708525 | GDPC |
| FAL1221 | 10200 | *Eupelmus fulvipes* | KJ018364 | KR348845 | KJ018548 | KR708433 | KR905346 | KR905259 | KR708478 | FALPC |
| LF.ro.RO.02 | 10656 | *Eupelmus fulvipes* | KJ018465 | KR348846 | KJ018620 | KR708434 | KR905347 | KR905260 | KR708529 | AICF |
| LF.ro.GE.01 | 10657 | *Eupelmus fulvipes* | KJ018466 | KR348847 | KJ018621 | KR708435 | KR905348 | KR905261 | KR708530 | AICF |
| FAL1004 | 10130 | *Eupelmus gemellus* | KJ018338 | KR348791 | KJ018499 | KR708379 | KR905296 | KR905208 | _ | MNHN |
| FAL1359 | 10230 | *Eupelmus gemellus* | KJ018380 | KR348789 | KJ018503 | KR708377 | KR905294 | KR905205 | KR708480 | MNHG |
| FAL1508 | 10405 | *Eupelmus gemellus* | KJ018405 | KR348792 | KJ018506 | KR708380 | KR905297 | KR905209 | KR708498 | CNC |
| NB441 | 10415 | *Eupelmus gemellus* | KR348764 | KR348790 | KR708350 | KR708378 | KR905295 | KR905206 | KR708502 | FALPC |
| FAL1260 | 10438 | *Eupelmus gemellus* | KR348765 | KR348788 | KR708352 | KR708376 | KR905293 | KR905207 | KR708509 | FALPC |
| GDEL4046 | 10032 | *Eupelmus janstai* | KJ018330 | KR348843 | KJ018526 | KR708431 | KR905344 | KR905257 | KR708457 | MNHG |
| GDEL4043 | 10028 | *Eupelmus kiefferi* | KJ018328 | KR348829 | KJ018477 | KR708417 | KR905332 | KR905244 | _ | GDPC |
| GDEL4045 | 10030 | *Eupelmus kiefferi* | KJ018329 | KR348823 | KJ018478 | KR708411 | KR905326 | KR905240 | KR708456 | GDPC |
| FAL1070 | 10151 | *Eupelmus kiefferi* | KR348753 | KR348820 | KR708339 | KR708408 | KR905323 | KR905235 | KR708473 | FALPC |
| FAL1109 | 10167 | *Eupelmus kiefferi* | KJ018354 | KR348821 | KJ018487 | KR708409 | KR905324 | KR905236 | KR708475 | FALPC |
| NB666 | 10325 | *Eupelmus kiefferi* | KJ018393 | KR348819 | KJ018495 | KR708407 | KR905322 | KR905234 | KR708491 | FALPC |
| NB674b | 10341 | *Eupelmus kiefferi* | KJ018397 | KR348818 | KJ018496 | KR708406 | KR905321 | KR905239 | KR708494 | CNC |
| FAL1511 | 10406 | *Eupelmus kiefferi* | KJ018406 | KR348822 | KJ018497 | KR708410 | KR905325 | KR905237 | KR708499 | FALPC |
| LF.ma.RO 01 | 10423 | *Eupelmus kiefferi* | _ | KR348825 | KR708351 | KR708413 | KR905328 | KR905242 | KR708503 | AICF |
| LF.fu.ES 01 | 10463 | *Eupelmus kiefferi* | KJ018436 | KR348830 | KJ018490 | KR708418 | KR905333 | KR905245 | _ | AICF |
| LF.fu.SL 01 | 10467 | *Eupelmus kiefferi* | KJ018437 | KR348828 | KJ018491 | KR708416 | KR905331 | KR905243 | KR708513 | AICF |
| MC-C124 | 10492 | *Eupelmus kiefferi* | KR348770 | KR348824 | KR708357 | KR708412 | KR905327 | KR905241 | KR708518 | FALPC |
| ZL.fu.RO.05 | 10585 | *Eupelmus kiefferi* | KJ018450 | KR348826 | KJ018492 | KR708414 | KR905329 | KR905238 | KR708524 | GDPC |
| FAL1524 | 10593 | *Eupelmus kiefferi* | KJ018451 | KR348831 | KJ018498 | KR708419 | KR905334 | KR905246 | _ | FALPC |
| LF.fu.GE 02 | 10658 | *Eupelmus kiefferi* | KJ018450 | KR348827 | KJ018492 | KR708415 | KR905330 | _ | KR708531 | AICF |
| GDEL4038 | 10019 | *Eupelmus longicalvus* | KJ018327 | KR348837 | KJ018525 | KR708425 | KR905338 | KR905252 | KR708455 | GDPC |
| LF.ma.SW 02 | 10429 | *Eupelmus longicalvus* | KJ018418 | KR348838 | KJ018588 | KR708426 | KR905339 | KR905251 | KR708508 | NHRS |
| GDEL4191 | 10603 | *Eupelmus longicalvus* | KJ018455 | KR348839 | KJ018611 | KR708427 | KR905340 | KR905253 | KR708527 | FALPC |
| FAL1491 | 10318 | *Eupelmus matranus* | KR348759 | KR348848 | KR708345 | KR708436 | KR905354 | KR905263 | KR708490 | FALPC |
| GDEL4116 | 10192 | *Eupelmus microzonus* | KR348754 | KR348849 | KR708340 | KR708437 | KR905353 | KR905262 | KR708476 | GDPC |
| GDEL4030 | 10009 | *Eupelmus minozonus* | KJ018323 | KR348815 | KJ018521 | KR708403 | KR905318 | KR905233 | KR708452 | GDPC |
| GDEL4030 | 10010 | *Eupelmus minozonus* | KJ018324 | KR348817 | KJ018522 | KR708404 | KR905319 | KR905232 | KR708453 | MNHG |
| GDEL4030 | 10011 | *Eupelmus minozonus* | KJ018325 | KR348816 | KJ018523 | KR708405 | KR905320 | KR905231 | KR708454 | MNHN |
| LF.ur.GR 01 | 10459 | *Eupelmus opacus* | KJ018434 | KR348833 | _ | KR708421 | KR905336 | KR905248 | _ | AICF |
| LF.ur.SW 02 | 10460 | *Eupelmus opacus* | KJ018435 | KR348834 | KJ018600 | KR708422 | KR905337 | _ | KR708512 | NHRS |
| GDEL4058 | 10048 | *Eupelmus pini* | KR348745 | KR348851 | KR708331 | KR708439 | KR905356 | KR905264 | KR708462 | GDPC |
| GDEL4027 | 10004 | *Eupelmus pistaciae* | KJ018321 | KR348785 | KJ018519 | KR708373 | KR905290 | KR905202 | KR708450 | FALPC |
| GDEL4027 | 10005 | *Eupelmus pistaciae* | KJ018322 | KR348786 | KJ018520 | KR708374 | KR905291 | KR905203 | KR708451 | FALPC |
| GDEL4027 | 10507 | *Eupelmus pistaciae* | KJ018444 | KR348787 | KJ018603 | KR708375 | KR905292 | KR905204 | KR708519 | MNHG |
| GDEL4051 | 10038 | *Eupelmus priotoni* | KJ018332 | KR348844 | KJ018528 | KR708432 | KR905345 | KR905258 | KR708459 | MNHG |
| LF.ur.GR.02 | 10650 | *Eupelmus purpuricollis* | KJ018460 | KR348835 | KJ018616 | KR708423 | _ | KR905249 | KR708528 | AICF |
| LF.ur.GR.03 | 10651 | *Eupelmus purpuricollis* | KJ018461 | KR348836 | KJ018617 | KR708424 | _ | KR905250 | _ | GDPC |
| GDEL4142 | 10297 | *Eupelmus simizonus* | KJ018388 | KR348832 | KJ018567 | KR708420 | KR905335 | KR905247 | KR708487 | MNHG |
| GDEL4148 | 10299 | *Eupelmus tibicinis* | KJ018389 | KR348841 | KJ018568 | KR708428 | KR905341 | KR905254 | KR708488 | GDPC |
| GDEL4149 | 10300 | *Eupelmus tibicinis* | KJ018390 | KR348842 | KJ018569 | KR708429 | KR905342 | KR905255 | KR708489 | FALPC |
| GDEL4175 | 10598 | *Eupelmus tibicinis* | KJ018454 | KR348840 | KJ018610 | KR708430 | KR905343 | KR905256 | KR708526 | GDPC |
| FAL1060 | 10148 | *Eupelmus urozonus* | KJ018346 | KR348807 | KJ018536 | KR708395 | KR905311 | KR905225 | KR708472 | FALPC |
| FAL1106 | 10165 | *Eupelmus urozonus* | KJ018353 | KR348809 | KJ018541 | KR708397 | KR905313 | KR905227 | KR708474 | FALPC |
| L.Loru235 | 10241 | *Eupelmus urozonus* | KR348756 | KR348808 | KR708342 | KR708396 | KR905312 | KR905226 | KR708483 | CBGP |
| NB1117 | 10251 | *Eupelmus urozonus* | KJ018387 | KR348810 | KJ018566 | KR708398 | KR905314 | KR905228 | KR708486 | FALPC |
| NB677 | 10333 | *Eupelmus urozonus* | KR348760 | KR348805 | KR708346 | KR708393 | KR905309 | KR905224 | KR708492 | MNHN |
| FAL1518 | 10410 | *Eupelmus urozonus* | KR348763 | KR348806 | KR708349 | KR708394 | KR905310 | KR905221 | KR708500 | CNC |
| LF.ur.IR 02 | 10457 | *Eupelmus urozonus* | KJ018433 | KR348814 | KJ018599 | KR708402 | _ | KR905230 | KR708511 | AICF |
| LF.fu.RO 01 | 10464 | *Eupelmus urozonus* | KR348766 | KR348813 | KR708353 | KR708401 | KR905317 | KR905222 | _ | AICF |
| MC-C100 | 10488 | *Eupelmus urozonus* | KJ018443 | KR348811 | KJ018602 | KR708399 | KR905315 | KR905223 | KR708517 | FALPC |
| PJ10077-2-6 | 10573 | *Eupelmus urozonus* | KJ018447 | KR348812 | KJ018605 | KR708400 | KR905316 | KR905229 | KR708520 | FALPC |
| GDEL4054 | 10042 | *Eupelmus vindex* | KR348744 | KR348802 | KR708330 | KR708390 | KR905306 | KR905218 | KR708461 | FALPC |
| LF.vi.RO 02 | 10468 | *Eupelmus vindex* | KR348767 | KR348803 | KR708354 | KR708391 | KR905307 | KR905219 | KR708514 | FALPC |
| LF.vi.RO 01 | 10469 | *Eupelmus vindex* | KR348768 | KR348804 | KR708355 | KR708392 | KR905308 | KR905220 | KR708515 | AICF |
| GDEL4088 | 10090 | *Eupelmus falcatus* | KR348749 | KR348852 | KR708335 | KR708440 | KR905357 | KR905265 | KR708466 | GDPC |
| GDEL4089 | 10091 | *Eupelmus seculatus* | KR348750 | KR348853 | KR708336 | KR708441 | KR905358 | KR905266 | KR708467 | FALPC |
| NB670 | 10336 | *Reikosiella aff. rostrata* | KR348761 | KR348860 | KR708347 | KR708446 | KR905349 | KR905271 | KR708493 | FALPC |
| NB810 | 10350 | *Reikosiella aff. rostrata* | KR348762 | KR348861 | KR708348 | KR708447 | KR905350 | KR905272 | KR708495 | FALPC |
