# Supplementary material for: Multilocus phylogeny and ecological differentiation of the “Eupelmus urozonus species group” (Hymenoptera, Eupelmidae) in the West-Palaearctic
Source: BMC Evol Biol. 2016 Jan 19;16:13. doi: 10.1186/s12862-015-0571-2 (PMC4717567; doi:10.1186/s12862-015-0571-2)

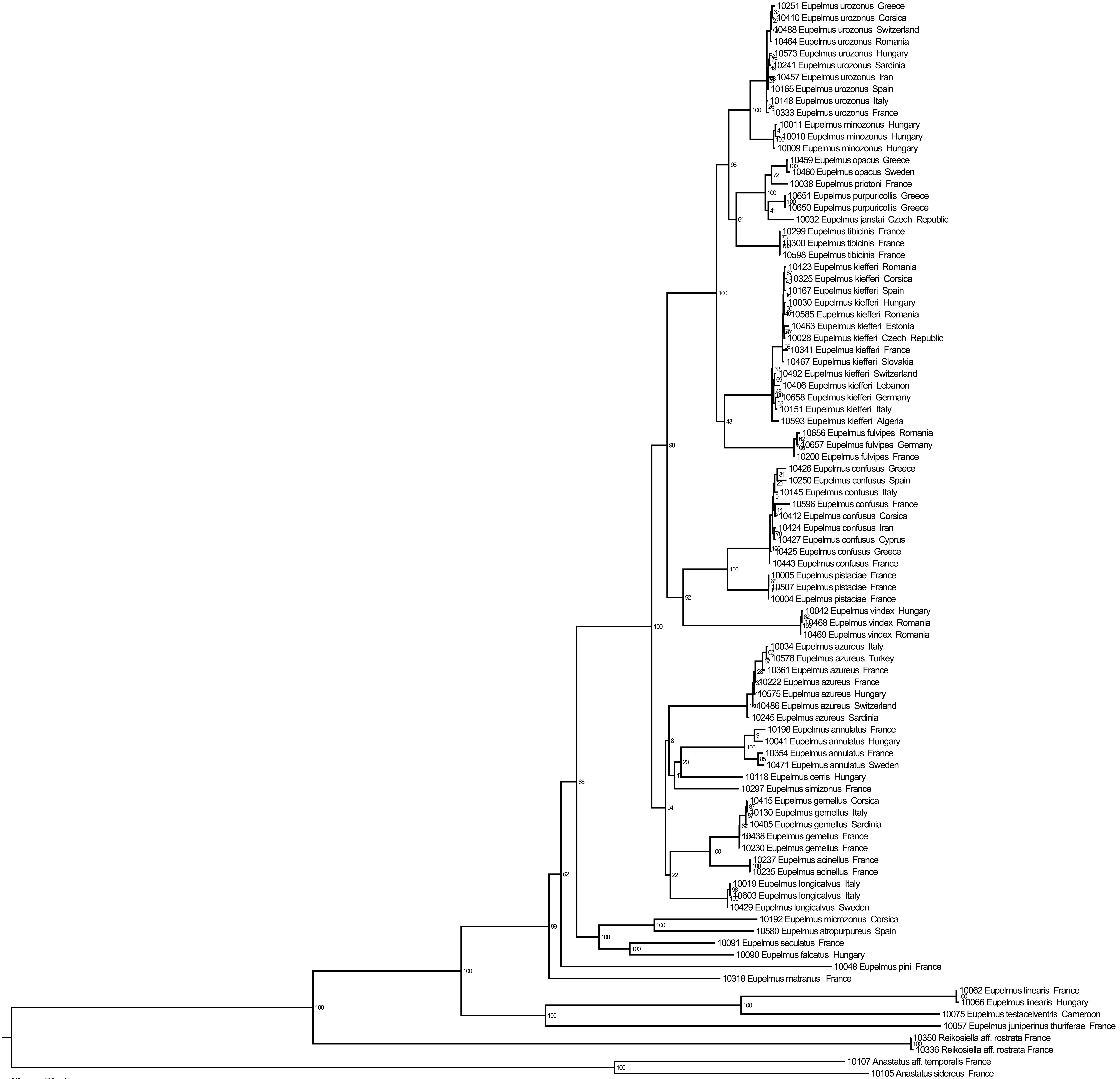
10251 Eupelmus urozonus Greece
10410 Eupelmus urozonus Corsica
10488 Eupelmus urozonus Switzerland
10464 Eupelmus urozonus Romania
10573 Eupelmus urozonus Hungary
10241 Eupelmus urozonus Sardinia
10457 Eupelmus urozonus Iran
10165 Eupelmus urozonus Spain
10148 Eupelmus urozonus Italy
10333 Eupelmus urozonus France
10011 Eupelmus minozonus Hungary
10010 Eupelmus minozonus Hungary
10009 Eupelmus minozonus Hungary
10459 Eupelmus opacus Greece
10460 Eupelmus opacus Sweden
10038 Eupelmus priotoni France
10651 Eupelmus purpuricollis Greece
10650 Eupelmus purpuricollis Greece
10032 Eupelmus janstai Czech Republic
10299 Eupelmus tibicinis France
10300 Eupelmus tibicinis France
10598 Eupelmus tibicinis France
10423 Eupelmus kiefferi Romania
10325 Eupelmus kiefferi Corsica
10167 Eupelmus kiefferi Spain
10030 Eupelmus kiefferi Hungary
10585 Eupelmus kiefferi Romania
10463 Eupelmus kiefferi Estonia
10028 Eupelmus kiefferi Czech Republic
10341 Eupelmus kiefferi France
10467 Eupelmus kiefferi Slovakia
10492 Eupelmus kiefferi Switzerland
10406 Eupelmus kiefferi Lebanon
10658 Eupelmus kiefferi Germany
10151 Eupelmus kiefferi Italy
10593 Eupelmus kiefferi Algeria
10656 Eupelmus fulvipes Romania
10657 Eupelmus fulvipes Germany
10200 Eupelmus fulvipes France
10426 Eupelmus confusus Greece
10250 Eupelmus confusus Spain
10145 Eupelmus confusus Italy
10596 Eupelmus confusus France
10412 Eupelmus confusus Corsica
10424 Eupelmus confusus Iran
10427 Eupelmus confusus Cyprus
10425 Eupelmus confusus Greece
10443 Eupelmus confusus France
10005 Eupelmus pistaciae France
10507 Eupelmus pistaciae France
10004 Eupelmus pistaciae France
10042 Eupelmus vindex Hungary
10468 Eupelmus vindex Romania
10469 Eupelmus vindex Romania
10034 Eupelmus azureus Italy
10578 Eupelmus azureus Turkey
10361 Eupelmus azureus France
10222 Eupelmus azureus France
10575 Eupelmus azureus Hungary
10486 Eupelmus azureus Switzerland
10245 Eupelmus azureus Sardinia
10198 Eupelmus annulatus France
10041 Eupelmus annulatus Hungary
10354 Eupelmus annulatus France
10471 Eupelmus annulatus Sweden
10118 Eupelmus cerris Hungary
10297 Eupelmus simizonus France
10415 Eupelmus gemellus Corsica
10130 Eupelmus gemellus Italy
10405 Eupelmus gemellus Sardinia
10438 Eupelmus gemellus France
10230 Eupelmus gemellus France
10237 Eupelmus acinellus France
10235 Eupelmus acinellus France
10019 Eupelmus longicalvus Italy
10603 Eupelmus longicalvus Italy
10429 Eupelmus longicalvus Sweden
10192 Eupelmus microzonus Corsica
10580 Eupelmus atropurpureus Spain
10091 Eupelmus seculatus France
10090 Eupelmus falcatus Hungary
10048 Eupelmus pini France
10318 Eupelmus matranus France
10062 Eupelmus linearis France
10066 Eupelmus linearis Hungary
10075 Eupelmus testaceiventris Cameroon
10057 Eupelmus juniperinus thuriferae France
10350 Reikosiella aff. rostrata France
10336 Reikosiella aff. rostrata France
10107 Anastatus aff. temporalis France
10105 Anastatus sidereus France
100
98
61
43
92
88
94
8
20
22
62
99
0.2

Figure S1 a)

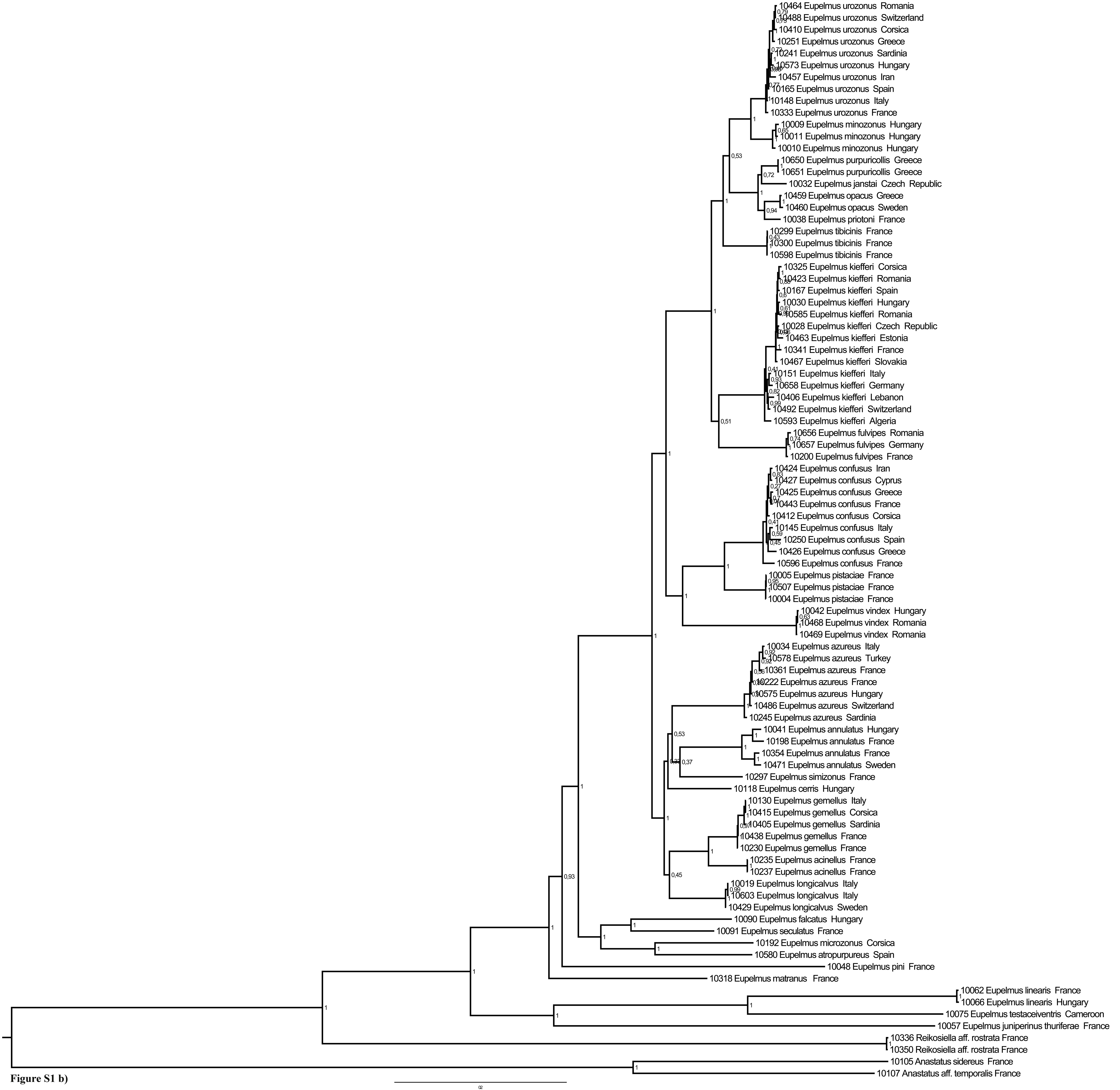

10464 Eupelmus urozonus Romania
10488 Eupelmus urozonus Switzerland
10410 Eupelmus urozonus Corsica
10251 Eupelmus urozonus Greece
10241 Eupelmus urozonus Sardinia
10573 Eupelmus urozonus Hungary
10457 Eupelmus urozonus Iran
10165 Eupelmus urozonus Spain
10148 Eupelmus urozonus Italy
10333 Eupelmus urozonus France
10009 Eupelmus minozonus Hungary
10011 Eupelmus minozonus Hungary
10010 Eupelmus minozonus Hungary
10650 Eupelmus purpuricollis Greece
10651 Eupelmus purpuricollis Greece
10032 Eupelmus janstai Czech Republic
10459 Eupelmus opacus Greece
10460 Eupelmus opacus Sweden
10038 Eupelmus priotoni France
10299 Eupelmus tibicinis France
10300 Eupelmus tibicinis France
10598 Eupelmus tibicinis France
10325 Eupelmus kiefferi Corsica
10423 Eupelmus kiefferi Romania
10167 Eupelmus kiefferi Spain
10030 Eupelmus kiefferi Hungary
10585 Eupelmus kiefferi Romania
10028 Eupelmus kiefferi Czech Republic
10463 Eupelmus kiefferi Estonia
10341 Eupelmus kiefferi France
10467 Eupelmus kiefferi Slovakia
10151 Eupelmus kiefferi Italy
10658 Eupelmus kiefferi Germany
10406 Eupelmus kiefferi Lebanon
10492 Eupelmus kiefferi Switzerland
10593 Eupelmus kiefferi Algeria
10656 Eupelmus fulvipes Romania
10657 Eupelmus fulvipes Germany
10200 Eupelmus fulvipes France
10424 Eupelmus confusus Iran
10427 Eupelmus confusus Cyprus
10425 Eupelmus confusus Greece
10443 Eupelmus confusus France
10412 Eupelmus confusus Corsica
10145 Eupelmus confusus Italy
10250 Eupelmus confusus Spain
10426 Eupelmus confusus Greece
10596 Eupelmus confusus France
10005 Eupelmus pistaciae France
10507 Eupelmus pistaciae France
10004 Eupelmus pistaciae France
10042 Eupelmus vindex Hungary
10468 Eupelmus vindex Romania
10469 Eupelmus vindex Romania
10034 Eupelmus azureus Italy
10578 Eupelmus azureus Turkey
10361 Eupelmus azureus France
10222 Eupelmus azureus France
10575 Eupelmus azureus Hungary
10486 Eupelmus azureus Switzerland
10245 Eupelmus azureus Sardinia
10041 Eupelmus annulatus Hungary
10198 Eupelmus annulatus France
10354 Eupelmus annulatus France
10471 Eupelmus annulatus Sweden
10297 Eupelmus simizonus France
10118 Eupelmus cerris Hungary
10130 Eupelmus gemellus Italy
10415 Eupelmus gemellus Corsica
10405 Eupelmus gemellus Sardinia
10438 Eupelmus gemellus France
10230 Eupelmus gemellus France
10235 Eupelmus acinellus France
10237 Eupelmus acinellus France
10019 Eupelmus longicalvus Italy
10603 Eupelmus longicalvus Italy
10429 Eupelmus longicalvus Sweden
10090 Eupelmus falcatus Hungary
10091 Eupelmus seculatus France
10192 Eupelmus microzonus Corsica
10580 Eupelmus atropurpureus Spain
10048 Eupelmus pini France
10318 Eupelmus matranus France
10062 Eupelmus linearis France
10066 Eupelmus linearis Hungary
10075 Eupelmus testaceiventris Cameroon
10057 Eupelmus juniperinus thuriferae France
10336 Reikosiella aff. rostrata France
10350 Reikosiella aff. rostrata France
10105 Anastatus sidereus France
10107 Anastatus aff. temporalis France

Figure S1 b)

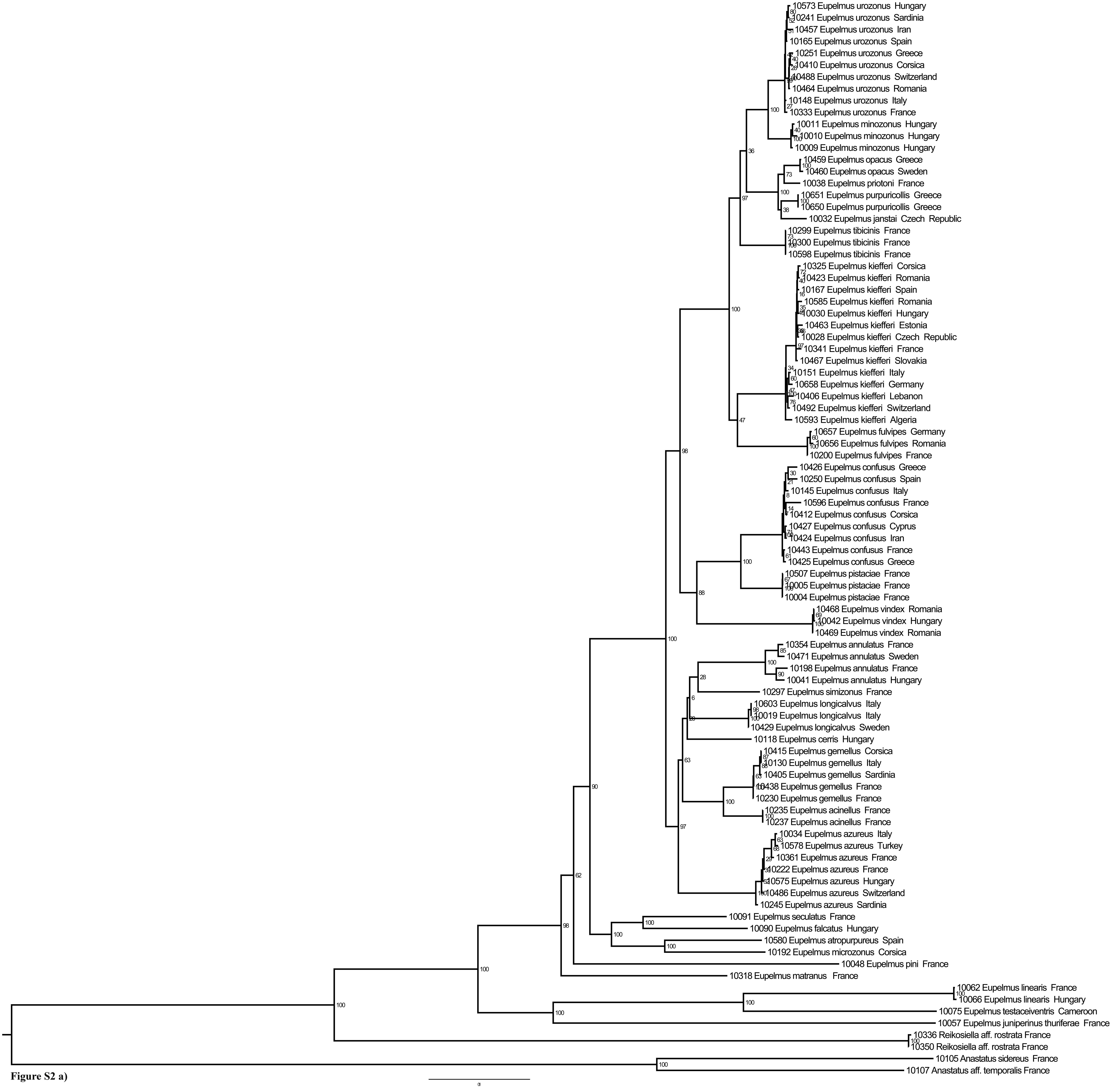
10573 Eupelmus urozonus Hungary
10241 Eupelmus urozonus Sardinia
10457 Eupelmus urozonus Iran
10165 Eupelmus urozonus Spain
10251 Eupelmus urozonus Greece
10410 Eupelmus urozonus Corsica
10488 Eupelmus urozonus Switzerland
10464 Eupelmus urozonus Romania
10148 Eupelmus urozonus Italy
10333 Eupelmus urozonus France
10011 Eupelmus minozonus Hungary
10010 Eupelmus minozonus Hungary
10009 Eupelmus minozonus Hungary
10459 Eupelmus opacus Greece
10460 Eupelmus opacus Sweden
10038 Eupelmus priotoni France
10651 Eupelmus purpuricollis Greece
10650 Eupelmus purpuricollis Greece
10032 Eupelmus janstai Czech Republic
10299 Eupelmus tibicinis France
10300 Eupelmus tibicinis France
10598 Eupelmus tibicinis France
10325 Eupelmus kiefferi Corsica
10423 Eupelmus kiefferi Romania
10167 Eupelmus kiefferi Spain
10585 Eupelmus kiefferi Romania
10030 Eupelmus kiefferi Hungary
10463 Eupelmus kiefferi Estonia
10028 Eupelmus kiefferi Czech Republic
10341 Eupelmus kiefferi France
10467 Eupelmus kiefferi Slovakia
10151 Eupelmus kiefferi Italy
10658 Eupelmus kiefferi Germany
10406 Eupelmus kiefferi Lebanon
10492 Eupelmus kiefferi Switzerland
10593 Eupelmus kiefferi Algeria
10657 Eupelmus fulvipes Germany
10656 Eupelmus fulvipes Romania
10200 Eupelmus fulvipes France
10426 Eupelmus confusus Greece
10250 Eupelmus confusus Spain
10145 Eupelmus confusus Italy
10596 Eupelmus confusus France
10412 Eupelmus confusus Corsica
10427 Eupelmus confusus Cyprus
10424 Eupelmus confusus Iran
10443 Eupelmus confusus France
10425 Eupelmus confusus Greece
10507 Eupelmus pistaciae France
10005 Eupelmus pistaciae France
10004 Eupelmus pistaciae France
10468 Eupelmus vindex Romania
10042 Eupelmus vindex Hungary
10469 Eupelmus vindex Romania
10354 Eupelmus annulatus France
10471 Eupelmus annulatus Sweden
10198 Eupelmus annulatus France
10041 Eupelmus annulatus Hungary
10297 Eupelmus simizonus France
10603 Eupelmus longicalvus Italy
10019 Eupelmus longicalvus Italy
10429 Eupelmus longicalvus Sweden
10118 Eupelmus cerris Hungary
10415 Eupelmus gemellus Corsica
10130 Eupelmus gemellus Italy
10405 Eupelmus gemellus Sardinia
10438 Eupelmus gemellus France
10230 Eupelmus gemellus France
10235 Eupelmus acinellus France
10237 Eupelmus acinellus France
10034 Eupelmus azureus Italy
10578 Eupelmus azureus Turkey
10361 Eupelmus azureus France
10222 Eupelmus azureus France
10575 Eupelmus azureus Hungary
10486 Eupelmus azureus Switzerland
10245 Eupelmus azureus Sardinia
10091 Eupelmus seculatus France
10090 Eupelmus falcatus Hungary
10580 Eupelmus atropurpureus Spain
10192 Eupelmus microzonus Corsica
10048 Eupelmus pini France
10318 Eupelmus matranus France
10062 Eupelmus linearis France
10066 Eupelmus linearis Hungary
10075 Eupelmus testaceiventris Cameroon
10057 Eupelmus juniperinus thuriferae France
10336 Reikosiella aff. rostrata France
10350 Reikosiella aff. rostrata France
10105 Anastatus sidereus France
10107 Anastatus aff. temporalis France

Figure S2 a)

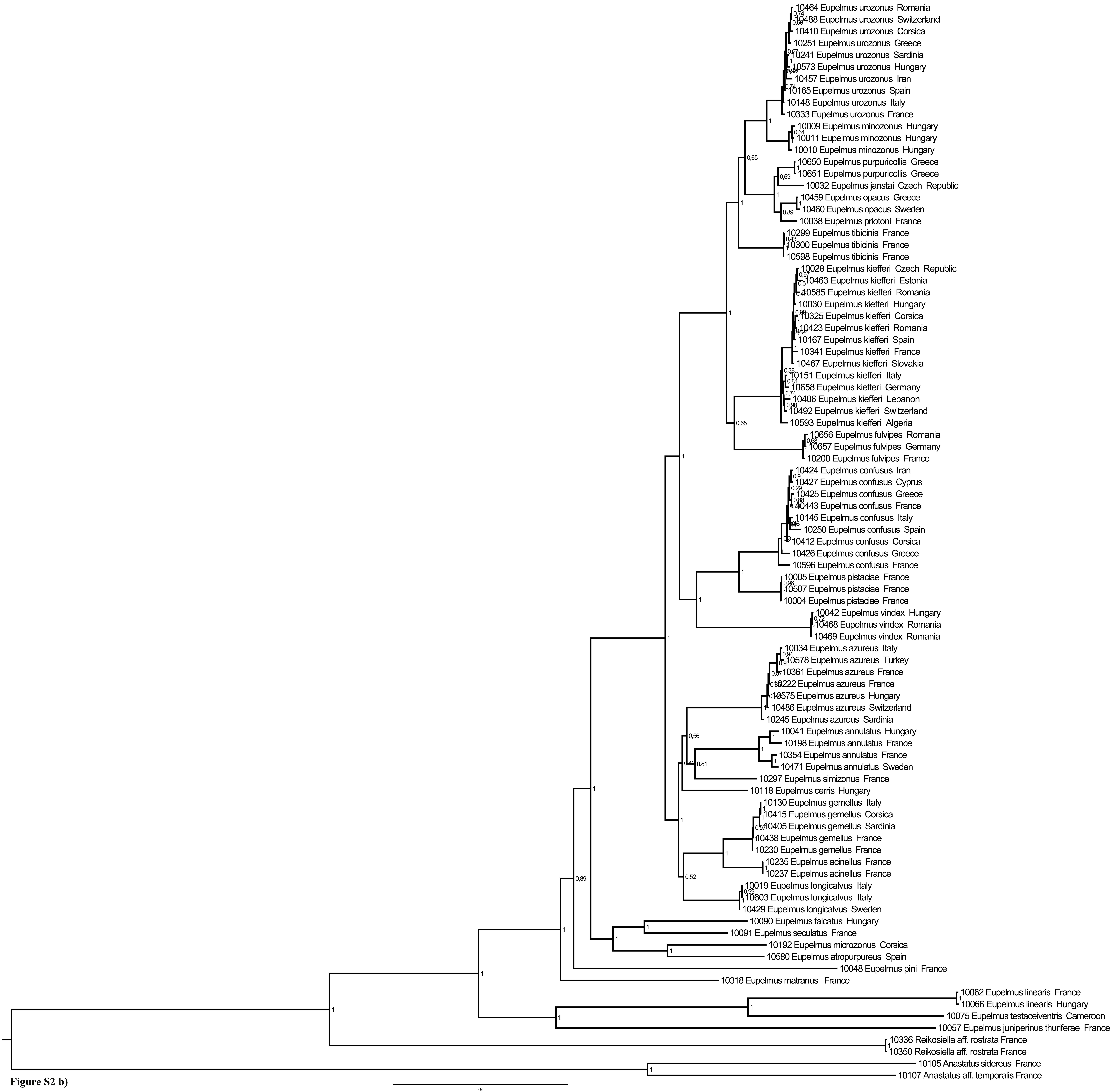

10464 Eupelmus urozonus Romania
10488 Eupelmus urozonus Switzerland
10410 Eupelmus urozonus Corsica
10251 Eupelmus urozonus Greece
10241 Eupelmus urozonus Sardinia
10573 Eupelmus urozonus Hungary
10457 Eupelmus urozonus Iran
10165 Eupelmus urozonus Spain
10148 Eupelmus urozonus Italy
10333 Eupelmus urozonus France
10009 Eupelmus minozonus Hungary
10011 Eupelmus minozonus Hungary
10010 Eupelmus minozonus Hungary
10650 Eupelmus purpuricollis Greece
10651 Eupelmus purpuricollis Greece
10032 Eupelmus janstai Czech Republic
10459 Eupelmus opacus Greece
10460 Eupelmus opacus Sweden
10038 Eupelmus priotoni France
10299 Eupelmus tibicinis France
10300 Eupelmus tibicinis France
10598 Eupelmus tibicinis France
10028 Eupelmus kiefferi Czech Republic
10463 Eupelmus kiefferi Estonia
10585 Eupelmus kiefferi Romania
10030 Eupelmus kiefferi Hungary
10325 Eupelmus kiefferi Corsica
10423 Eupelmus kiefferi Romania
10167 Eupelmus kiefferi Spain
10341 Eupelmus kiefferi France
10467 Eupelmus kiefferi Slovakia
10151 Eupelmus kiefferi Italy
10658 Eupelmus kiefferi Germany
10406 Eupelmus kiefferi Lebanon
10492 Eupelmus kiefferi Switzerland
10593 Eupelmus kiefferi Algeria
10656 Eupelmus fulvipes Romania
10657 Eupelmus fulvipes Germany
10200 Eupelmus fulvipes France
10424 Eupelmus confusus Iran
10427 Eupelmus confusus Cyprus
10425 Eupelmus confusus Greece
10443 Eupelmus confusus France
10145 Eupelmus confusus Italy
10250 Eupelmus confusus Spain
10412 Eupelmus confusus Corsica
10426 Eupelmus confusus Greece
10596 Eupelmus confusus France
10005 Eupelmus pistaciae France
10507 Eupelmus pistaciae France
10004 Eupelmus pistaciae France
10042 Eupelmus vindex Hungary
10468 Eupelmus vindex Romania
10469 Eupelmus vindex Romania
10034 Eupelmus azureus Italy
10578 Eupelmus azureus Turkey
10361 Eupelmus azureus France
10222 Eupelmus azureus France
10575 Eupelmus azureus Hungary
10486 Eupelmus azureus Switzerland
10245 Eupelmus azureus Sardinia
10041 Eupelmus annulatus Hungary
10198 Eupelmus annulatus France
10354 Eupelmus annulatus France
10471 Eupelmus annulatus Sweden
10297 Eupelmus simizonus France
10118 Eupelmus cerris Hungary
10130 Eupelmus gemellus Italy
10415 Eupelmus gemellus Corsica
10405 Eupelmus gemellus Sardinia
10438 Eupelmus gemellus France
10230 Eupelmus gemellus France
10235 Eupelmus acinellus France
10237 Eupelmus acinellus France
10019 Eupelmus longicalvus Italy
10603 Eupelmus longicalvus Italy
10429 Eupelmus longicalvus Sweden
10090 Eupelmus falcatus Hungary
10091 Eupelmus seculatus France
10192 Eupelmus microzonus Corsica
10580 Eupelmus atropurpureus Spain
10048 Eupelmus pini France
10318 Eupelmus matranus France
10062 Eupelmus linearis France
10066 Eupelmus linearis Hungary
10075 Eupelmus testaceiventris Cameroon
10057 Eupelmus juniperinus thuriferae France
10336 Reikosiella aff. rostrata France
10350 Reikosiella aff. rostrata France
10105 Anastatus sidereus France
10107 Anastatus aff. temporalis France
0,65
0,69
0,89
0,65
0,56
0,81
0,52
0,89
0,2

Figure S2 b)

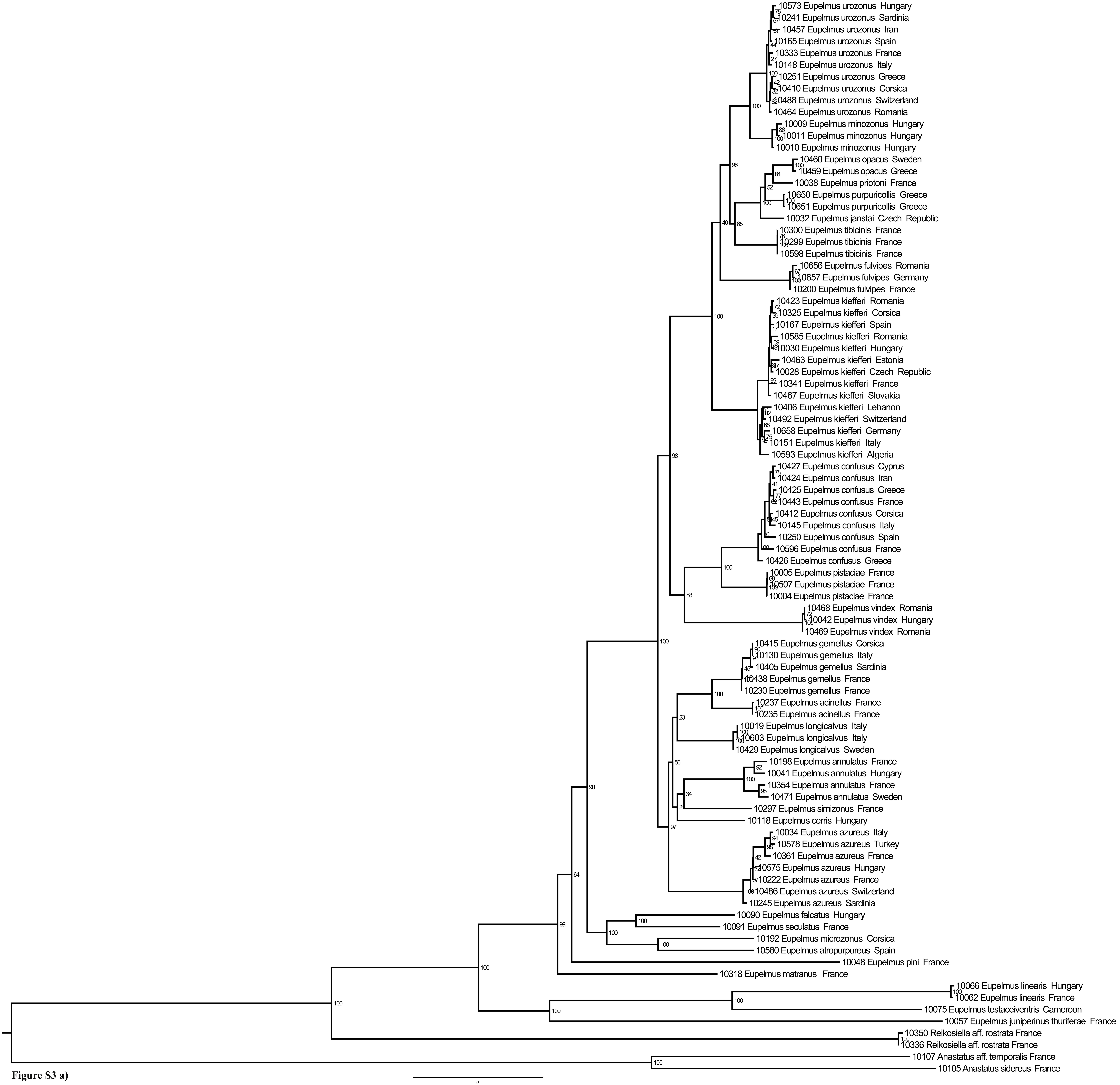
10573 Eupelmus urozonus Hungary
10241 Eupelmus urozonus Sardinia
10457 Eupelmus urozonus Iran
10165 Eupelmus urozonus Spain
10333 Eupelmus urozonus France
10148 Eupelmus urozonus Italy
10251 Eupelmus urozonus Greece
10410 Eupelmus urozonus Corsica
10488 Eupelmus urozonus Switzerland
10464 Eupelmus urozonus Romania
10009 Eupelmus minozonus Hungary
10011 Eupelmus minozonus Hungary
10010 Eupelmus minozonus Hungary
10460 Eupelmus opacus Sweden
10459 Eupelmus opacus Greece
10038 Eupelmus priotoni France
10650 Eupelmus purpuricollis Greece
10651 Eupelmus purpuricollis Greece
10032 Eupelmus janstai Czech Republic
10300 Eupelmus tibicinis France
10299 Eupelmus tibicinis France
10598 Eupelmus tibicinis France
10656 Eupelmus fulvipes Romania
10657 Eupelmus fulvipes Germany
10200 Eupelmus fulvipes France
10423 Eupelmus kiefferi Romania
10325 Eupelmus kiefferi Corsica
10167 Eupelmus kiefferi Spain
10585 Eupelmus kiefferi Romania
10030 Eupelmus kiefferi Hungary
10463 Eupelmus kiefferi Estonia
10028 Eupelmus kiefferi Czech Republic
10341 Eupelmus kiefferi France
10467 Eupelmus kiefferi Slovakia
10406 Eupelmus kiefferi Lebanon
10492 Eupelmus kiefferi Switzerland
10658 Eupelmus kiefferi Germany
10151 Eupelmus kiefferi Italy
10593 Eupelmus kiefferi Algeria
10427 Eupelmus confusus Cyprus
10424 Eupelmus confusus Iran
10425 Eupelmus confusus Greece
10443 Eupelmus confusus France
10412 Eupelmus confusus Corsica
10145 Eupelmus confusus Italy
10250 Eupelmus confusus Spain
10596 Eupelmus confusus France
10426 Eupelmus confusus Greece
10005 Eupelmus pistaciae France
10507 Eupelmus pistaciae France
10004 Eupelmus pistaciae France
10468 Eupelmus vindex Romania
10042 Eupelmus vindex Hungary
10469 Eupelmus vindex Romania
10415 Eupelmus gemellus Corsica
10130 Eupelmus gemellus Italy
10405 Eupelmus gemellus Sardinia
10438 Eupelmus gemellus France
10230 Eupelmus gemellus France
10237 Eupelmus acinellus France
10235 Eupelmus acinellus France
10019 Eupelmus longicalvus Italy
10603 Eupelmus longicalvus Italy
10429 Eupelmus longicalvus Sweden
10198 Eupelmus annulatus France
10041 Eupelmus annulatus Hungary
10354 Eupelmus annulatus France
10471 Eupelmus annulatus Sweden
10297 Eupelmus simizonus France
10118 Eupelmus cerris Hungary
10034 Eupelmus azureus Italy
10578 Eupelmus azureus Turkey
10361 Eupelmus azureus France
10575 Eupelmus azureus Hungary
10222 Eupelmus azureus France
10486 Eupelmus azureus Switzerland
10245 Eupelmus azureus Sardinia
10090 Eupelmus falcatus Hungary
10091 Eupelmus seculatus France
10192 Eupelmus microzonus Corsica
10580 Eupelmus atropurpureus Spain
10048 Eupelmus pini France
10318 Eupelmus matranus France
10066 Eupelmus linearis Hungary
10062 Eupelmus linearis France
10075 Eupelmus testaceiventris Cameroon
10057 Eupelmus juniperinus thuriferae France
10350 Reikosiella aff. rostrata France
10336 Reikosiella aff. rostrata France
10107 Anastatus aff. temporalis France
10105 Anastatus sidereus France

Figure S3 a)

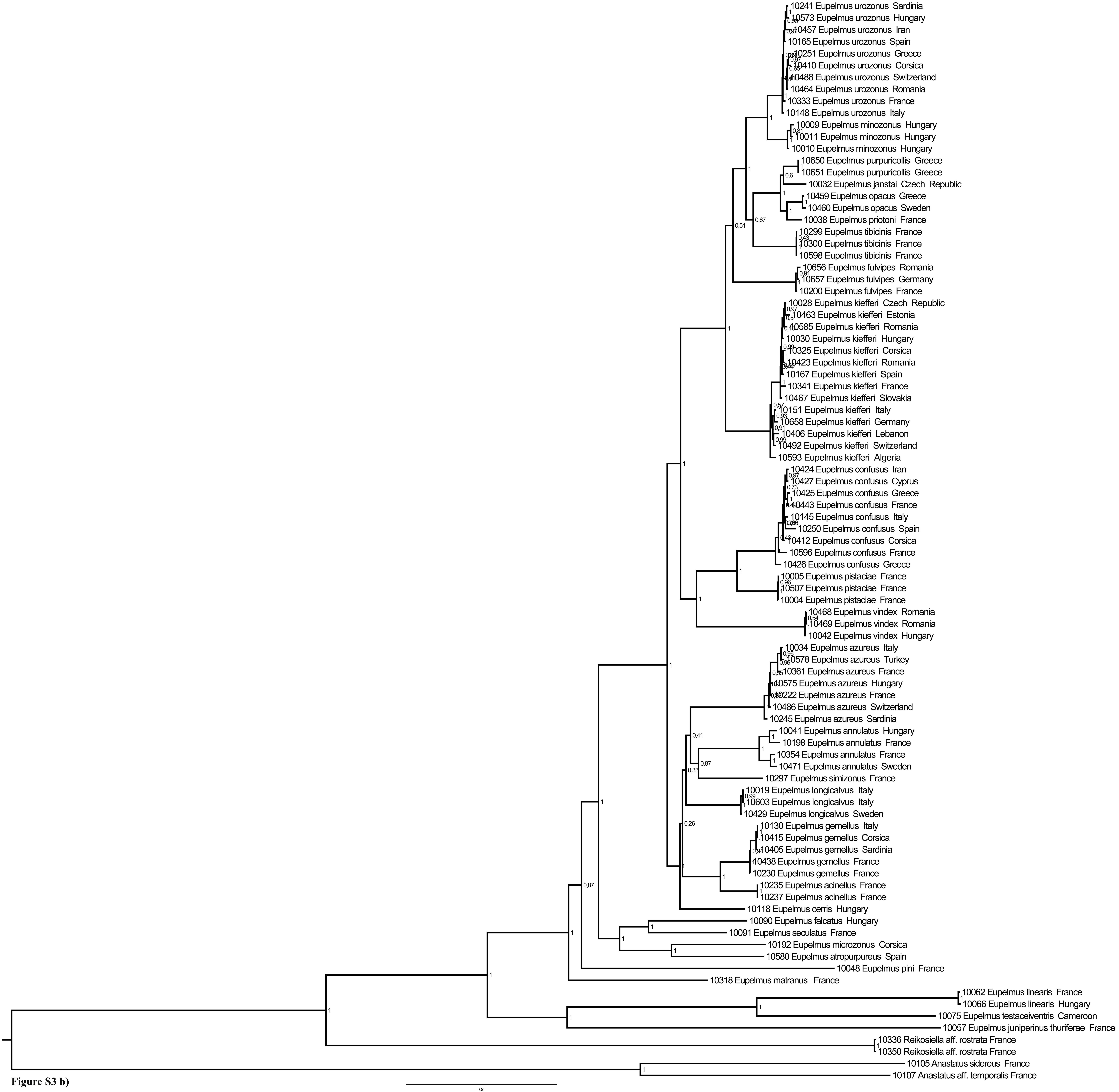
10241 Eupelmus urozonus Sardinia
10573 Eupelmus urozonus Hungary
10457 Eupelmus urozonus Iran
10165 Eupelmus urozonus Spain
10251 Eupelmus urozonus Greece
10410 Eupelmus urozonus Corsica
10488 Eupelmus urozonus Switzerland
10464 Eupelmus urozonus Romania
10333 Eupelmus urozonus France
10148 Eupelmus urozonus Italy
10009 Eupelmus minozonus Hungary
10011 Eupelmus minozonus Hungary
10010 Eupelmus minozonus Hungary
10650 Eupelmus purpuricollis Greece
10651 Eupelmus purpuricollis Greece
10032 Eupelmus janstai Czech Republic
10459 Eupelmus opacus Greece
10460 Eupelmus opacus Sweden
10038 Eupelmus priotoni France
10299 Eupelmus tibicinis France
10300 Eupelmus tibicinis France
10598 Eupelmus tibicinis France
10656 Eupelmus fulvipes Romania
10657 Eupelmus fulvipes Germany
10200 Eupelmus fulvipes France
10028 Eupelmus kiefferi Czech Republic
10463 Eupelmus kiefferi Estonia
10585 Eupelmus kiefferi Romania
10030 Eupelmus kiefferi Hungary
10325 Eupelmus kiefferi Corsica
10423 Eupelmus kiefferi Romania
10167 Eupelmus kiefferi Spain
10341 Eupelmus kiefferi France
10467 Eupelmus kiefferi Slovakia
10151 Eupelmus kiefferi Italy
10658 Eupelmus kiefferi Germany
10406 Eupelmus kiefferi Lebanon
10492 Eupelmus kiefferi Switzerland
10593 Eupelmus kiefferi Algeria
10424 Eupelmus confusus Iran
10427 Eupelmus confusus Cyprus
10425 Eupelmus confusus Greece
10443 Eupelmus confusus France
10145 Eupelmus confusus Italy
10250 Eupelmus confusus Spain
10412 Eupelmus confusus Corsica
10596 Eupelmus confusus France
10426 Eupelmus confusus Greece
10005 Eupelmus pistaciae France
10507 Eupelmus pistaciae France
10004 Eupelmus pistaciae France
10468 Eupelmus vindex Romania
10469 Eupelmus vindex Romania
10042 Eupelmus vindex Hungary
10034 Eupelmus azureus Italy
10578 Eupelmus azureus Turkey
10361 Eupelmus azureus France
10575 Eupelmus azureus Hungary
10222 Eupelmus azureus France
10486 Eupelmus azureus Switzerland
10245 Eupelmus azureus Sardinia
10041 Eupelmus annulatus Hungary
10198 Eupelmus annulatus France
10354 Eupelmus annulatus France
10471 Eupelmus annulatus Sweden
10297 Eupelmus simizonus France
10019 Eupelmus longicalvus Italy
10603 Eupelmus longicalvus Italy
10429 Eupelmus longicalvus Sweden
10130 Eupelmus gemellus Italy
10415 Eupelmus gemellus Corsica
10405 Eupelmus gemellus Sardinia
10438 Eupelmus gemellus France
10230 Eupelmus gemellus France
10235 Eupelmus acinellus France
10237 Eupelmus acinellus France
10118 Eupelmus cerris Hungary
10090 Eupelmus falcatus Hungary
10091 Eupelmus seculatus France
10192 Eupelmus microzonus Corsica
10580 Eupelmus atropurpureus Spain
10048 Eupelmus pini France
10318 Eupelmus matranus France
10062 Eupelmus linearis France
10066 Eupelmus linearis Hungary
10075 Eupelmus testaceiventris Cameroon
10057 Eupelmus juniperinus thuriferae France
10336 Reikosiella aff. rostrata France
10350 Reikosiella aff. rostrata France
10105 Anastatus sidereus France
10107 Anastatus aff. temporalis France

Figure S3 b)

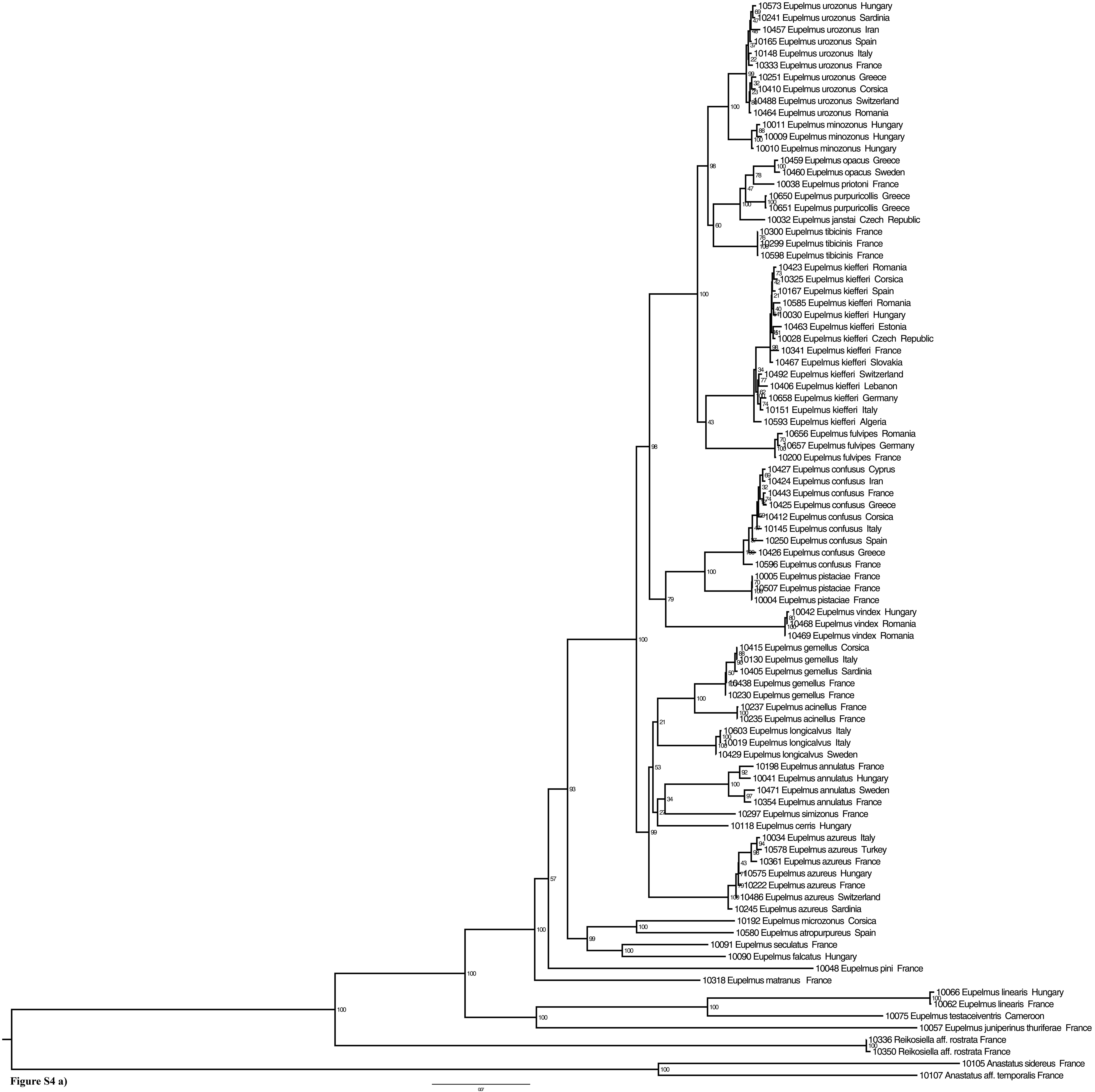
10573 Eupelmus urozonus Hungary
10241 Eupelmus urozonus Sardinia
10457 Eupelmus urozonus Iran
10165 Eupelmus urozonus Spain
10148 Eupelmus urozonus Italy
10333 Eupelmus urozonus France
10251 Eupelmus urozonus Greece
10410 Eupelmus urozonus Corsica
10488 Eupelmus urozonus Switzerland
10464 Eupelmus urozonus Romania
10011 Eupelmus minozonus Hungary
10009 Eupelmus minozonus Hungary
10010 Eupelmus minozonus Hungary
10459 Eupelmus opacus Greece
10460 Eupelmus opacus Sweden
10038 Eupelmus priotoni France
10650 Eupelmus purpuricollis Greece
10651 Eupelmus purpuricollis Greece
10032 Eupelmus janstai Czech Republic
10300 Eupelmus tibicinis France
10299 Eupelmus tibicinis France
10598 Eupelmus tibicinis France
10423 Eupelmus kiefferi Romania
10325 Eupelmus kiefferi Corsica
10167 Eupelmus kiefferi Spain
10585 Eupelmus kiefferi Romania
10030 Eupelmus kiefferi Hungary
10463 Eupelmus kiefferi Estonia
10028 Eupelmus kiefferi Czech Republic
10341 Eupelmus kiefferi France
10467 Eupelmus kiefferi Slovakia
10492 Eupelmus kiefferi Switzerland
10406 Eupelmus kiefferi Lebanon
10658 Eupelmus kiefferi Germany
10151 Eupelmus kiefferi Italy
10593 Eupelmus kiefferi Algeria
10656 Eupelmus fulvipes Romania
10657 Eupelmus fulvipes Germany
10200 Eupelmus fulvipes France
10427 Eupelmus confusus Cyprus
10424 Eupelmus confusus Iran
10443 Eupelmus confusus France
10425 Eupelmus confusus Greece
10412 Eupelmus confusus Corsica
10145 Eupelmus confusus Italy
10250 Eupelmus confusus Spain
10426 Eupelmus confusus Greece
10596 Eupelmus confusus France
10005 Eupelmus pistaciae France
10507 Eupelmus pistaciae France
10004 Eupelmus pistaciae France
10042 Eupelmus vindex Hungary
10468 Eupelmus vindex Romania
10469 Eupelmus vindex Romania
10415 Eupelmus gemellus Corsica
10130 Eupelmus gemellus Italy
10405 Eupelmus gemellus Sardinia
10438 Eupelmus gemellus France
10230 Eupelmus gemellus France
10237 Eupelmus acinellus France
10235 Eupelmus acinellus France
10603 Eupelmus longicalvus Italy
10019 Eupelmus longicalvus Italy
10429 Eupelmus longicalvus Sweden
10198 Eupelmus annulatus France
10041 Eupelmus annulatus Hungary
10471 Eupelmus annulatus Sweden
10354 Eupelmus annulatus France
10297 Eupelmus simizonus France
10118 Eupelmus cerris Hungary
10034 Eupelmus azureus Italy
10578 Eupelmus azureus Turkey
10361 Eupelmus azureus France
10575 Eupelmus azureus Hungary
10222 Eupelmus azureus France
10486 Eupelmus azureus Switzerland
10245 Eupelmus azureus Sardinia
10192 Eupelmus microzonus Corsica
10580 Eupelmus atropurpureus Spain
10091 Eupelmus seculatus France
10090 Eupelmus falcatus Hungary
10048 Eupelmus pini France
10318 Eupelmus matranus France
10066 Eupelmus linearis Hungary
10062 Eupelmus linearis France
10075 Eupelmus testaceiventris Cameroon
10057 Eupelmus juniperinus thuriferae France
10336 Reikosiella aff. rostrata France
10350 Reikosiella aff. rostrata France
10105 Anastatus sidereus France
10107 Anastatus aff. temporalis France

Figure S4 a)

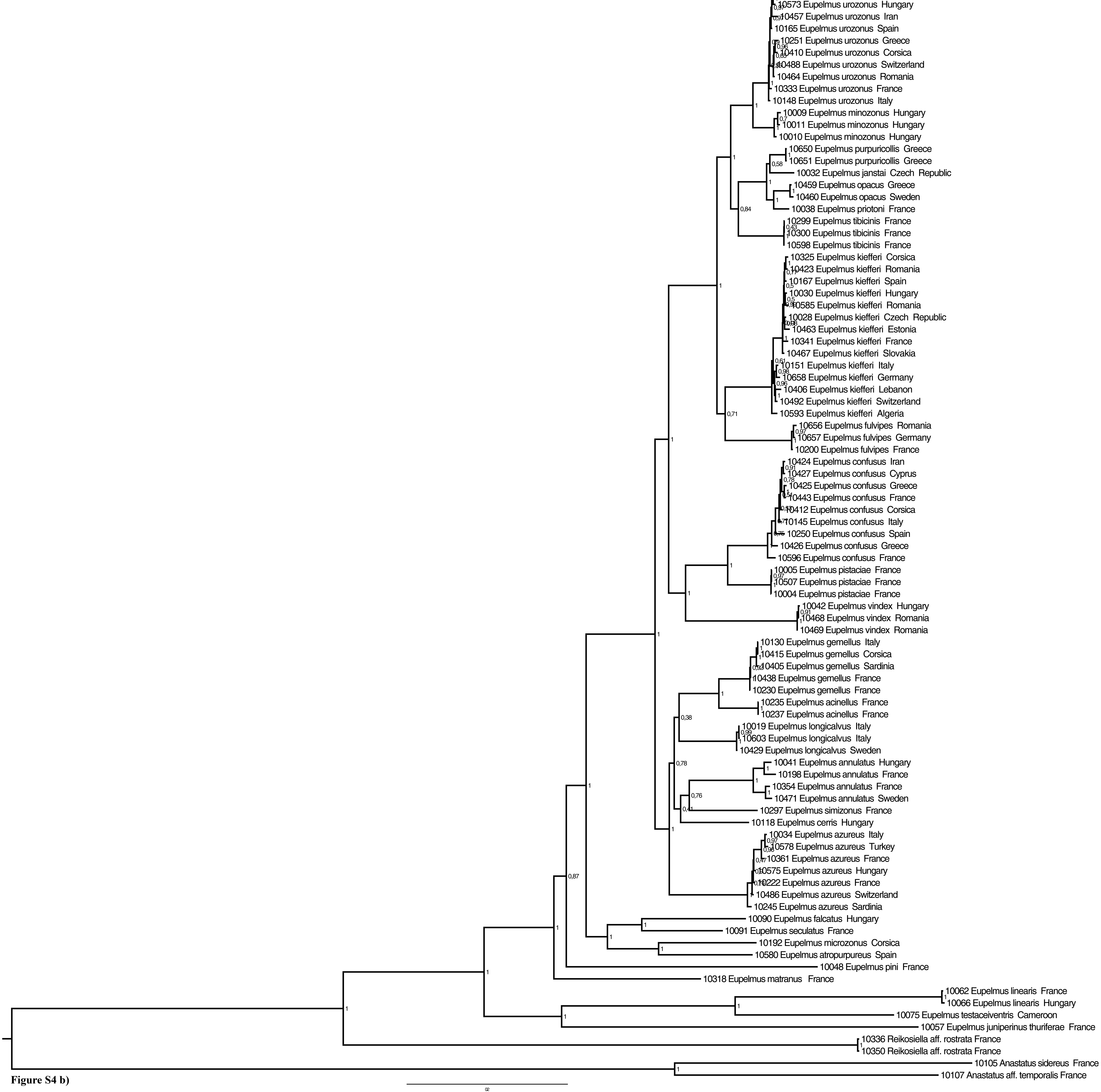
10241 Eupelmus urozonus Sardinia
10573 Eupelmus urozonus Hungary
10457 Eupelmus urozonus Iran
10165 Eupelmus urozonus Spain
10251 Eupelmus urozonus Greece
10410 Eupelmus urozonus Corsica
10488 Eupelmus urozonus Switzerland
10464 Eupelmus urozonus Romania
10333 Eupelmus urozonus France
10148 Eupelmus urozonus Italy
10009 Eupelmus minozonus Hungary
10011 Eupelmus minozonus Hungary
10010 Eupelmus minozonus Hungary
10650 Eupelmus purpuricollis Greece
10651 Eupelmus purpuricollis Greece
10032 Eupelmus janstai Czech Republic
10459 Eupelmus opacus Greece
10460 Eupelmus opacus Sweden
10038 Eupelmus priotoni France
10299 Eupelmus tibicinis France
10300 Eupelmus tibicinis France
10598 Eupelmus tibicinis France
10325 Eupelmus kiefferi Corsica
10423 Eupelmus kiefferi Romania
10167 Eupelmus kiefferi Spain
10030 Eupelmus kiefferi Hungary
10585 Eupelmus kiefferi Romania
10028 Eupelmus kiefferi Czech Republic
10463 Eupelmus kiefferi Estonia
10341 Eupelmus kiefferi France
10467 Eupelmus kiefferi Slovakia
10151 Eupelmus kiefferi Italy
10658 Eupelmus kiefferi Germany
10406 Eupelmus kiefferi Lebanon
10492 Eupelmus kiefferi Switzerland
10593 Eupelmus kiefferi Algeria
10656 Eupelmus fulvipes Romania
10657 Eupelmus fulvipes Germany
10200 Eupelmus fulvipes France
10424 Eupelmus confusus Iran
10427 Eupelmus confusus Cyprus
10425 Eupelmus confusus Greece
10443 Eupelmus confusus France
10412 Eupelmus confusus Corsica
10145 Eupelmus confusus Italy
10250 Eupelmus confusus Spain
10426 Eupelmus confusus Greece
10596 Eupelmus confusus France
10005 Eupelmus pistaciae France
10507 Eupelmus pistaciae France
10004 Eupelmus pistaciae France
10042 Eupelmus vindex Hungary
10468 Eupelmus vindex Romania
10469 Eupelmus vindex Romania
10130 Eupelmus gemellus Italy
10415 Eupelmus gemellus Corsica
10405 Eupelmus gemellus Sardinia
10438 Eupelmus gemellus France
10230 Eupelmus gemellus France
10235 Eupelmus acinellus France
10237 Eupelmus acinellus France
10019 Eupelmus longicalvus Italy
10603 Eupelmus longicalvus Italy
10429 Eupelmus longicalvus Sweden
10041 Eupelmus annulatus Hungary
10198 Eupelmus annulatus France
10354 Eupelmus annulatus France
10471 Eupelmus annulatus Sweden
10297 Eupelmus simizonus France
10118 Eupelmus cerris Hungary
10034 Eupelmus azureus Italy
10578 Eupelmus azureus Turkey
10361 Eupelmus azureus France
10575 Eupelmus azureus Hungary
10222 Eupelmus azureus France
10486 Eupelmus azureus Switzerland
10245 Eupelmus azureus Sardinia
10090 Eupelmus falcatus Hungary
10091 Eupelmus seculatus France
10192 Eupelmus microzonus Corsica
10580 Eupelmus atropurpureus Spain
10048 Eupelmus pini France
10318 Eupelmus matranus France
10062 Eupelmus linearis France
10066 Eupelmus linearis Hungary
10075 Eupelmus testaceiventris Cameroon
10057 Eupelmus juniperinus thuriferae France
10336 Reikosiella aff. rostrata France
10350 Reikosiella aff. rostrata France
10105 Anastatus sidereus France
10107 Anastatus aff. temporalis France

Figure S4 b)

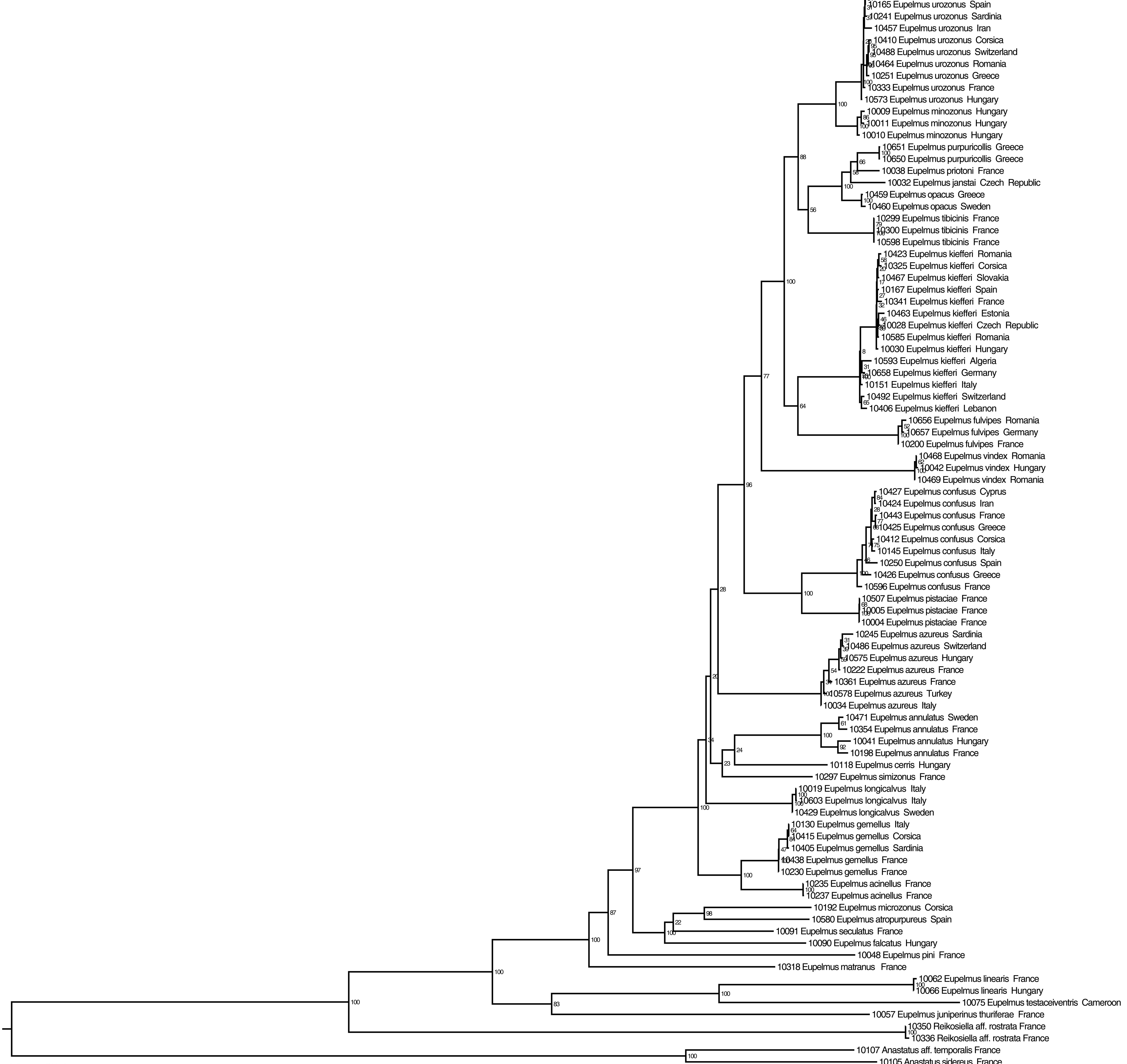
10148 Eupelmus urozonus Italy
10165 Eupelmus urozonus Spain
10241 Eupelmus urozonus Sardinia
10457 Eupelmus urozonus Iran
10410 Eupelmus urozonus Corsica
10488 Eupelmus urozonus Switzerland
10464 Eupelmus urozonus Romania
10251 Eupelmus urozonus Greece
10333 Eupelmus urozonus France
10573 Eupelmus urozonus Hungary
10009 Eupelmus minozonus Hungary
10011 Eupelmus minozonus Hungary
10010 Eupelmus minozonus Hungary
10651 Eupelmus purpuricollis Greece
10650 Eupelmus purpuricollis Greece
10038 Eupelmus priotoni France
10032 Eupelmus janstai Czech Republic
10459 Eupelmus opacus Greece
10460 Eupelmus opacus Sweden
10299 Eupelmus tibicinis France
10300 Eupelmus tibicinis France
10598 Eupelmus tibicinis France
10423 Eupelmus kiefferi Romania
10325 Eupelmus kiefferi Corsica
10467 Eupelmus kiefferi Slovakia
10167 Eupelmus kiefferi Spain
10341 Eupelmus kiefferi France
10463 Eupelmus kiefferi Estonia
10028 Eupelmus kiefferi Czech Republic
10585 Eupelmus kiefferi Romania
10030 Eupelmus kiefferi Hungary
10593 Eupelmus kiefferi Algeria
10658 Eupelmus kiefferi Germany
10151 Eupelmus kiefferi Italy
10492 Eupelmus kiefferi Switzerland
10406 Eupelmus kiefferi Lebanon
10656 Eupelmus fulvipes Romania
10657 Eupelmus fulvipes Germany
10200 Eupelmus fulvipes France
10468 Eupelmus vindex Romania
10042 Eupelmus vindex Hungary
10469 Eupelmus vindex Romania
10427 Eupelmus confusus Cyprus
10424 Eupelmus confusus Iran
10443 Eupelmus confusus France
10425 Eupelmus confusus Greece
10412 Eupelmus confusus Corsica
10145 Eupelmus confusus Italy
10250 Eupelmus confusus Spain
10426 Eupelmus confusus Greece
10596 Eupelmus confusus France
10507 Eupelmus pistaciae France
10005 Eupelmus pistaciae France
10004 Eupelmus pistaciae France
10245 Eupelmus azureus Sardinia
10486 Eupelmus azureus Switzerland
10575 Eupelmus azureus Hungary
10222 Eupelmus azureus France
10361 Eupelmus azureus France
10578 Eupelmus azureus Turkey
10034 Eupelmus azureus Italy
10471 Eupelmus annulatus Sweden
10354 Eupelmus annulatus France
10041 Eupelmus annulatus Hungary
10198 Eupelmus annulatus France
10118 Eupelmus cerris Hungary
10297 Eupelmus simizonus France
10019 Eupelmus longicalvus Italy
10603 Eupelmus longicalvus Italy
10429 Eupelmus longicalvus Sweden
10130 Eupelmus gemellus Italy
10415 Eupelmus gemellus Corsica
10405 Eupelmus gemellus Sardinia
10438 Eupelmus gemellus France
10230 Eupelmus gemellus France
10235 Eupelmus acinellus France
10237 Eupelmus acinellus France
10192 Eupelmus microzonus Corsica
10580 Eupelmus atropurpureus Spain
10091 Eupelmus seculatus France
10090 Eupelmus falcatus Hungary
10048 Eupelmus pini France
10318 Eupelmus matranus France
10062 Eupelmus linearis France
10066 Eupelmus linearis Hungary
10075 Eupelmus testaceiventris Cameroon
10057 Eupelmus juniperinus thuriferae France
10350 Reikosiella aff. rostrata France
10336 Reikosiella aff. rostrata France
10107 Anastatus aff. temporalis France
10105 Anastatus sidereus France

Figure S5 a)

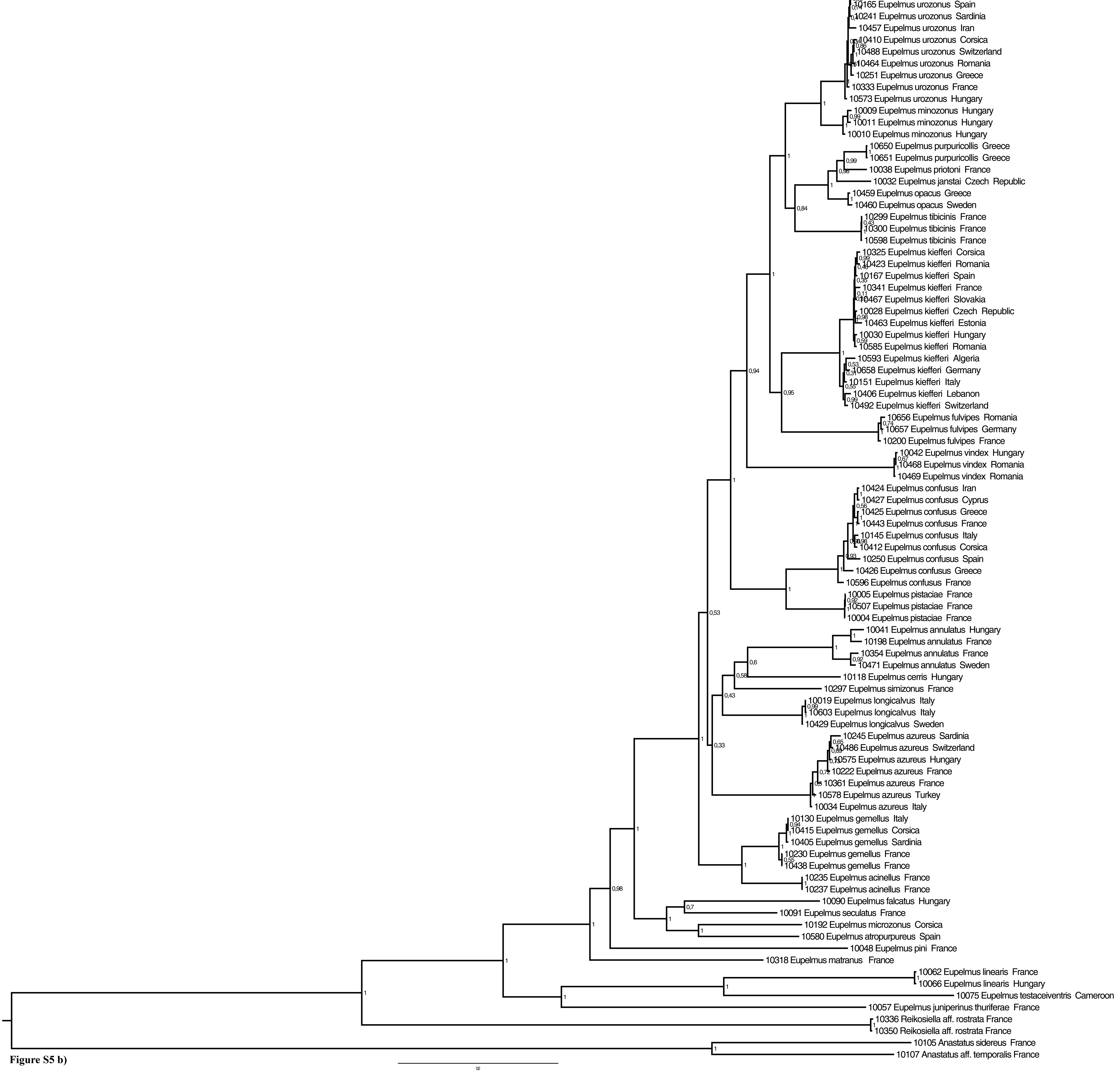
10148 Eupelmus urozonus Italy
10165 Eupelmus urozonus Spain
10241 Eupelmus urozonus Sardinia
10457 Eupelmus urozonus Iran
10410 Eupelmus urozonus Corsica
10488 Eupelmus urozonus Switzerland
10464 Eupelmus urozonus Romania
10251 Eupelmus urozonus Greece
10333 Eupelmus urozonus France
10573 Eupelmus urozonus Hungary
10009 Eupelmus minozonus Hungary
10011 Eupelmus minozonus Hungary
10010 Eupelmus minozonus Hungary
10650 Eupelmus purpuricollis Greece
10651 Eupelmus purpuricollis Greece
10038 Eupelmus priotoni France
10032 Eupelmus janstai Czech Republic
10459 Eupelmus opacus Greece
10460 Eupelmus opacus Sweden
10299 Eupelmus tibicinis France
10300 Eupelmus tibicinis France
10598 Eupelmus tibicinis France
10325 Eupelmus kiefferi Corsica
10423 Eupelmus kiefferi Romania
10167 Eupelmus kiefferi Spain
10341 Eupelmus kiefferi France
10467 Eupelmus kiefferi Slovakia
10028 Eupelmus kiefferi Czech Republic
10463 Eupelmus kiefferi Estonia
10030 Eupelmus kiefferi Hungary
10585 Eupelmus kiefferi Romania
10593 Eupelmus kiefferi Algeria
10658 Eupelmus kiefferi Germany
10151 Eupelmus kiefferi Italy
10406 Eupelmus kiefferi Lebanon
10492 Eupelmus kiefferi Switzerland
10656 Eupelmus fulvipes Romania
10657 Eupelmus fulvipes Germany
10200 Eupelmus fulvipes France
10042 Eupelmus vindex Hungary
10468 Eupelmus vindex Romania
10469 Eupelmus vindex Romania
10424 Eupelmus confusus Iran
10427 Eupelmus confusus Cyprus
10425 Eupelmus confusus Greece
10443 Eupelmus confusus France
10145 Eupelmus confusus Italy
10412 Eupelmus confusus Corsica
10250 Eupelmus confusus Spain
10426 Eupelmus confusus Greece
10596 Eupelmus confusus France
10005 Eupelmus pistaciae France
10507 Eupelmus pistaciae France
10004 Eupelmus pistaciae France
10041 Eupelmus annulatus Hungary
10198 Eupelmus annulatus France
10354 Eupelmus annulatus France
10471 Eupelmus annulatus Sweden
10118 Eupelmus cerris Hungary
10297 Eupelmus simizonus France
10019 Eupelmus longicalvus Italy
10603 Eupelmus longicalvus Italy
10429 Eupelmus longicalvus Sweden
10245 Eupelmus azureus Sardinia
10486 Eupelmus azureus Switzerland
10575 Eupelmus azureus Hungary
10222 Eupelmus azureus France
10361 Eupelmus azureus France
10578 Eupelmus azureus Turkey
10034 Eupelmus azureus Italy
10130 Eupelmus gemellus Italy
10415 Eupelmus gemellus Corsica
10405 Eupelmus gemellus Sardinia
10230 Eupelmus gemellus France
10438 Eupelmus gemellus France
10235 Eupelmus acinellus France
10237 Eupelmus acinellus France
10090 Eupelmus falcatus Hungary
10091 Eupelmus seculatus France
10192 Eupelmus microzonus Corsica
10580 Eupelmus atropurpureus Spain
10048 Eupelmus pini France
10318 Eupelmus matranus France
10062 Eupelmus linearis France
10066 Eupelmus linearis Hungary
10075 Eupelmus testaceiventris Cameroon
10057 Eupelmus juniperinus thuriferae France
10336 Reikosiella aff. rostrata France
10350 Reikosiella aff. rostrata France
10105 Anastatus sidereus France
10107 Anastatus aff. temporalis France
0.2

Figure S5 b)

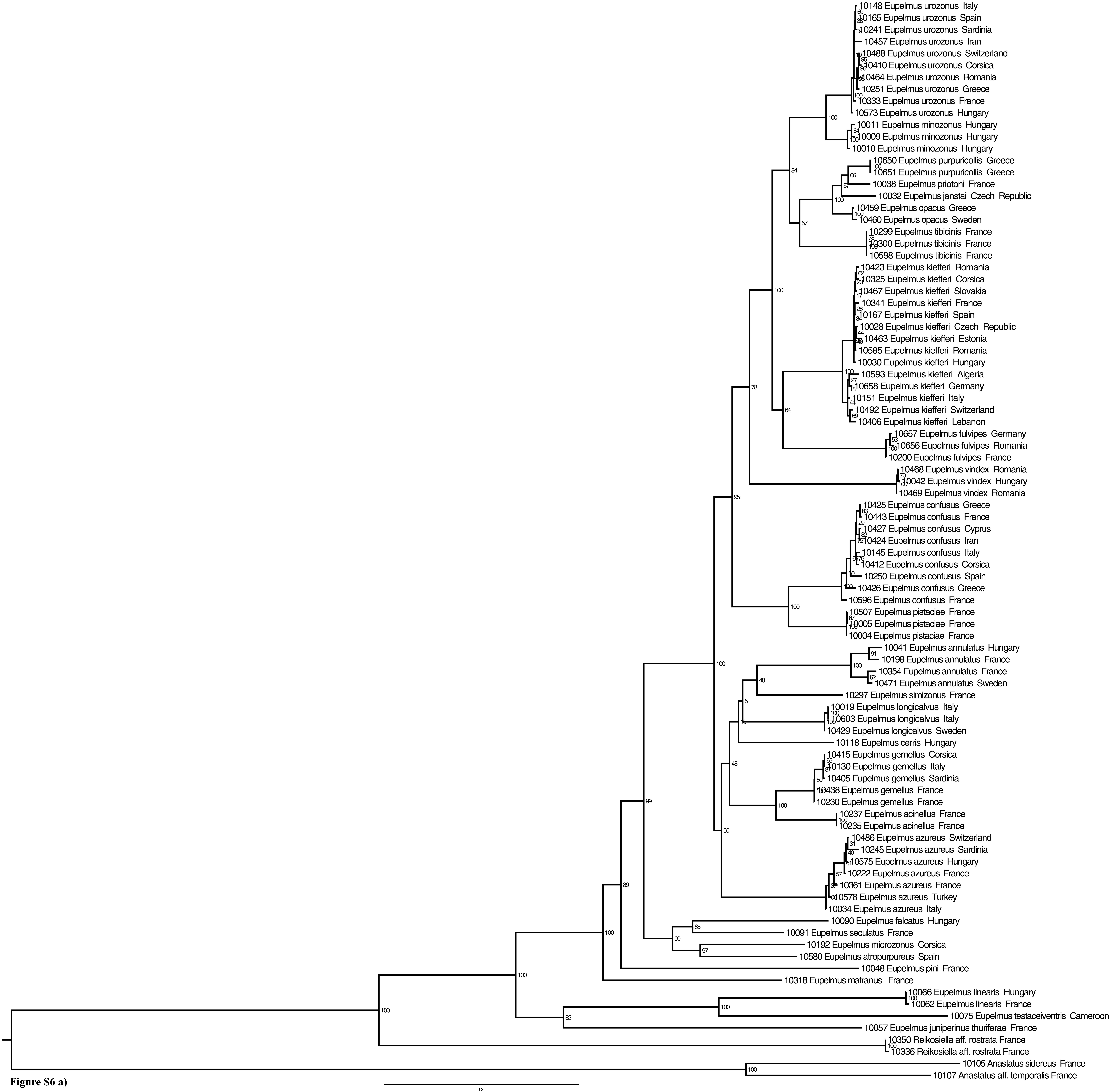
10148 Eupelmus urozonus Italy
10165 Eupelmus urozonus Spain
10241 Eupelmus urozonus Sardinia
10457 Eupelmus urozonus Iran
10488 Eupelmus urozonus Switzerland
10410 Eupelmus urozonus Corsica
10464 Eupelmus urozonus Romania
10251 Eupelmus urozonus Greece
10333 Eupelmus urozonus France
10573 Eupelmus urozonus Hungary
10011 Eupelmus minozonus Hungary
10009 Eupelmus minozonus Hungary
10010 Eupelmus minozonus Hungary
10650 Eupelmus purpuricollis Greece
10651 Eupelmus purpuricollis Greece
10038 Eupelmus priotoni France
10032 Eupelmus janstai Czech Republic
10459 Eupelmus opacus Greece
10460 Eupelmus opacus Sweden
10299 Eupelmus tibicinis France
10300 Eupelmus tibicinis France
10598 Eupelmus tibicinis France
10423 Eupelmus kiefferi Romania
10325 Eupelmus kiefferi Corsica
10467 Eupelmus kiefferi Slovakia
10341 Eupelmus kiefferi France
10167 Eupelmus kiefferi Spain
10028 Eupelmus kiefferi Czech Republic
10463 Eupelmus kiefferi Estonia
10585 Eupelmus kiefferi Romania
10030 Eupelmus kiefferi Hungary
10593 Eupelmus kiefferi Algeria
10658 Eupelmus kiefferi Germany
10151 Eupelmus kiefferi Italy
10492 Eupelmus kiefferi Switzerland
10406 Eupelmus kiefferi Lebanon
10657 Eupelmus fulvipes Germany
10656 Eupelmus fulvipes Romania
10200 Eupelmus fulvipes France
10468 Eupelmus vindex Romania
10042 Eupelmus vindex Hungary
10469 Eupelmus vindex Romania
10425 Eupelmus confusus Greece
10443 Eupelmus confusus France
10427 Eupelmus confusus Cyprus
10424 Eupelmus confusus Iran
10145 Eupelmus confusus Italy
10412 Eupelmus confusus Corsica
10250 Eupelmus confusus Spain
10426 Eupelmus confusus Greece
10596 Eupelmus confusus France
10507 Eupelmus pistaciae France
10005 Eupelmus pistaciae France
10004 Eupelmus pistaciae France
10041 Eupelmus annulatus Hungary
10198 Eupelmus annulatus France
10354 Eupelmus annulatus France
10471 Eupelmus annulatus Sweden
10297 Eupelmus simizonus France
10019 Eupelmus longicalvus Italy
10603 Eupelmus longicalvus Italy
10429 Eupelmus longicalvus Sweden
10118 Eupelmus cerris Hungary
10415 Eupelmus gemellus Corsica
10130 Eupelmus gemellus Italy
10405 Eupelmus gemellus Sardinia
10438 Eupelmus gemellus France
10230 Eupelmus gemellus France
10237 Eupelmus acinellus France
10235 Eupelmus acinellus France
10486 Eupelmus azureus Switzerland
10245 Eupelmus azureus Sardinia
10575 Eupelmus azureus Hungary
10222 Eupelmus azureus France
10361 Eupelmus azureus France
10578 Eupelmus azureus Turkey
10034 Eupelmus azureus Italy
10090 Eupelmus falcatus Hungary
10091 Eupelmus seculatus France
10192 Eupelmus microzonus Corsica
10580 Eupelmus atropurpureus Spain
10048 Eupelmus pini France
10318 Eupelmus matranus France
10066 Eupelmus linearis Hungary
10062 Eupelmus linearis France
10075 Eupelmus testaceiventris Cameroon
10057 Eupelmus juniperinus thuriferae France
10350 Reikosiella aff. rostrata France
10336 Reikosiella aff. rostrata France
10105 Anastatus sidereus France
10107 Anastatus aff. temporalis France
0.2

Figure S6 a)

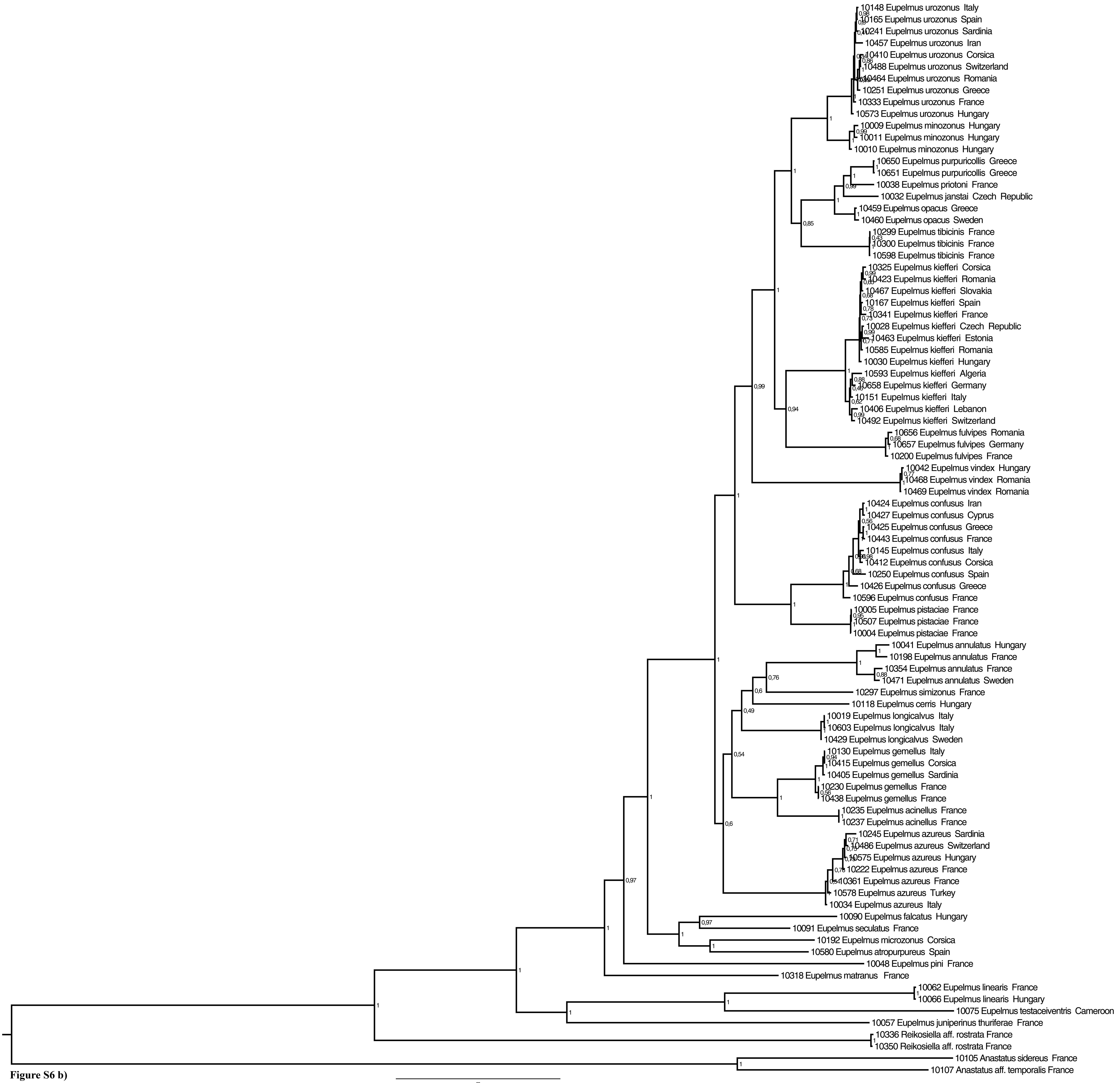
10148 Eupelmus urozonus Italy
10165 Eupelmus urozonus Spain
10241 Eupelmus urozonus Sardinia
10457 Eupelmus urozonus Iran
10410 Eupelmus urozonus Corsica
10488 Eupelmus urozonus Switzerland
10464 Eupelmus urozonus Romania
10251 Eupelmus urozonus Greece
10333 Eupelmus urozonus France
10573 Eupelmus urozonus Hungary
10009 Eupelmus minozonus Hungary
10011 Eupelmus minozonus Hungary
10010 Eupelmus minozonus Hungary
10650 Eupelmus purpuricollis Greece
10651 Eupelmus purpuricollis Greece
10038 Eupelmus priotoni France
10032 Eupelmus janstai Czech Republic
10459 Eupelmus opacus Greece
10460 Eupelmus opacus Sweden
10299 Eupelmus tibicinis France
10300 Eupelmus tibicinis France
10598 Eupelmus tibicinis France
10325 Eupelmus kiefferi Corsica
10423 Eupelmus kiefferi Romania
10467 Eupelmus kiefferi Slovakia
10167 Eupelmus kiefferi Spain
10341 Eupelmus kiefferi France
10028 Eupelmus kiefferi Czech Republic
10463 Eupelmus kiefferi Estonia
10585 Eupelmus kiefferi Romania
10030 Eupelmus kiefferi Hungary
10593 Eupelmus kiefferi Algeria
10658 Eupelmus kiefferi Germany
10151 Eupelmus kiefferi Italy
10406 Eupelmus kiefferi Lebanon
10492 Eupelmus kiefferi Switzerland
10656 Eupelmus fulvipes Romania
10657 Eupelmus fulvipes Germany
10200 Eupelmus fulvipes France
10042 Eupelmus vindex Hungary
10468 Eupelmus vindex Romania
10469 Eupelmus vindex Romania
10424 Eupelmus confusus Iran
10427 Eupelmus confusus Cyprus
10425 Eupelmus confusus Greece
10443 Eupelmus confusus France
10145 Eupelmus confusus Italy
10412 Eupelmus confusus Corsica
10250 Eupelmus confusus Spain
10426 Eupelmus confusus Greece
10596 Eupelmus confusus France
10005 Eupelmus pistaciae France
10507 Eupelmus pistaciae France
10004 Eupelmus pistaciae France
10041 Eupelmus annulatus Hungary
10198 Eupelmus annulatus France
10354 Eupelmus annulatus France
10471 Eupelmus annulatus Sweden
10297 Eupelmus simizonus France
10118 Eupelmus cerris Hungary
10019 Eupelmus longicalvus Italy
10603 Eupelmus longicalvus Italy
10429 Eupelmus longicalvus Sweden
10130 Eupelmus gemellus Italy
10415 Eupelmus gemellus Corsica
10405 Eupelmus gemellus Sardinia
10230 Eupelmus gemellus France
10438 Eupelmus gemellus France
10235 Eupelmus acinellus France
10237 Eupelmus acinellus France
10245 Eupelmus azureus Sardinia
10486 Eupelmus azureus Switzerland
10575 Eupelmus azureus Hungary
10222 Eupelmus azureus France
10361 Eupelmus azureus France
10578 Eupelmus azureus Turkey
10034 Eupelmus azureus Italy
10090 Eupelmus falcatus Hungary
10091 Eupelmus seculatus France
10192 Eupelmus microzonus Corsica
10580 Eupelmus atropurpureus Spain
10048 Eupelmus pini France
10318 Eupelmus matranus France
10062 Eupelmus linearis France
10066 Eupelmus linearis Hungary
10075 Eupelmus testaceiventris Cameroon
10057 Eupelmus juniperinus thuriferae France
10336 Reikosiella aff. rostrata France
10350 Reikosiella aff. rostrata France
10105 Anastatus sidereus France
10107 Anastatus aff. temporalis France
0.2

Figure S6 b)

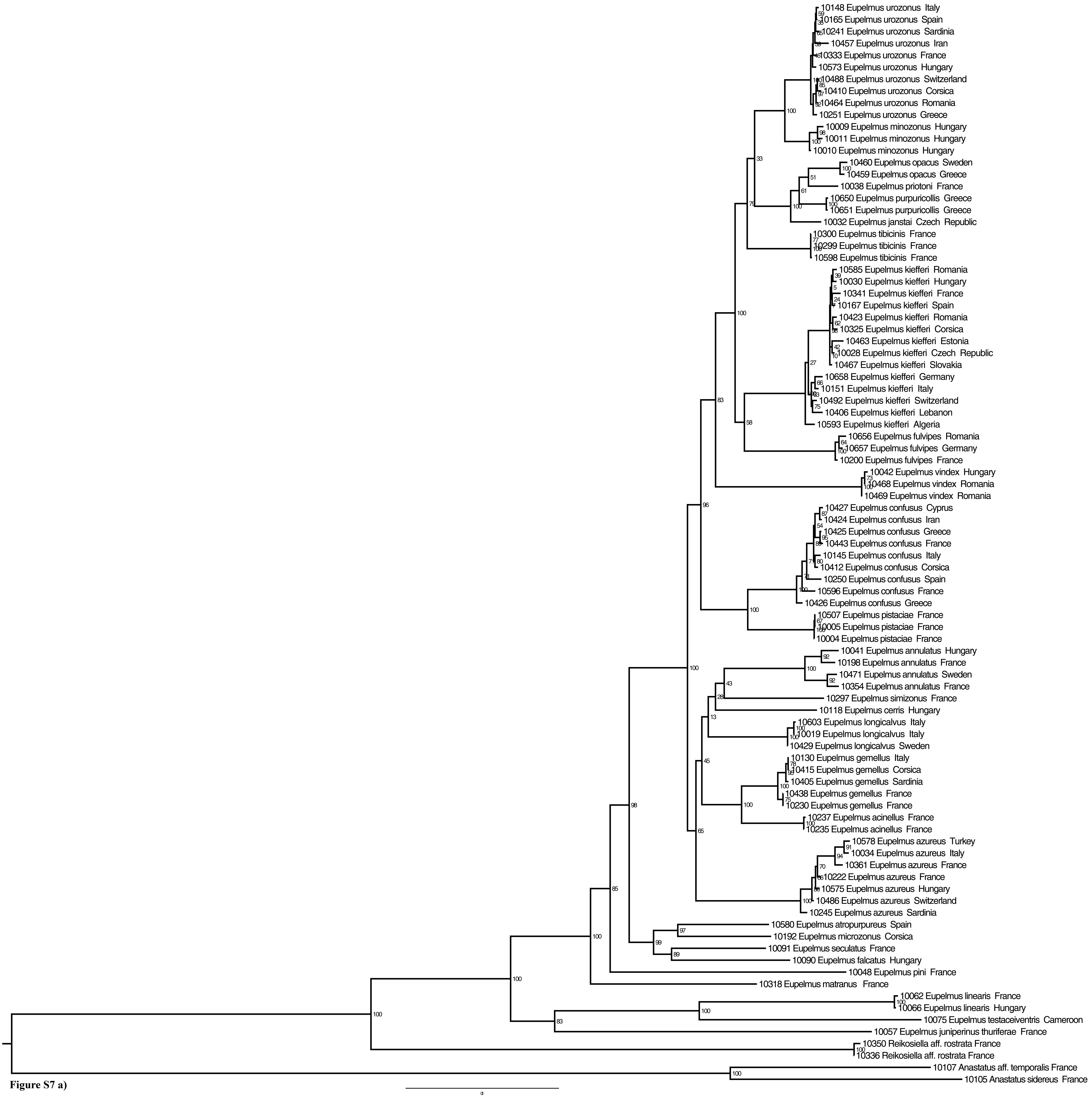
10148 Eupelmus urozonus Italy
10165 Eupelmus urozonus Spain
10241 Eupelmus urozonus Sardinia
10457 Eupelmus urozonus Iran
10333 Eupelmus urozonus France
10573 Eupelmus urozonus Hungary
10488 Eupelmus urozonus Switzerland
10410 Eupelmus urozonus Corsica
10464 Eupelmus urozonus Romania
10251 Eupelmus urozonus Greece
10009 Eupelmus minozonus Hungary
10011 Eupelmus minozonus Hungary
10010 Eupelmus minozonus Hungary
10460 Eupelmus opacus Sweden
10459 Eupelmus opacus Greece
10038 Eupelmus priotoni France
10650 Eupelmus purpuricollis Greece
10651 Eupelmus purpuricollis Greece
10032 Eupelmus janstai Czech Republic
10300 Eupelmus tibicinis France
10299 Eupelmus tibicinis France
10598 Eupelmus tibicinis France
10585 Eupelmus kiefferi Romania
10030 Eupelmus kiefferi Hungary
10341 Eupelmus kiefferi France
10167 Eupelmus kiefferi Spain
10423 Eupelmus kiefferi Romania
10325 Eupelmus kiefferi Corsica
10463 Eupelmus kiefferi Estonia
10028 Eupelmus kiefferi Czech Republic
10467 Eupelmus kiefferi Slovakia
10658 Eupelmus kiefferi Germany
10151 Eupelmus kiefferi Italy
10492 Eupelmus kiefferi Switzerland
10406 Eupelmus kiefferi Lebanon
10593 Eupelmus kiefferi Algeria
10656 Eupelmus fulvipes Romania
10657 Eupelmus fulvipes Germany
10200 Eupelmus fulvipes France
10042 Eupelmus vindex Hungary
10468 Eupelmus vindex Romania
10469 Eupelmus vindex Romania
10427 Eupelmus confusus Cyprus
10424 Eupelmus confusus Iran
10425 Eupelmus confusus Greece
10443 Eupelmus confusus France
10145 Eupelmus confusus Italy
10412 Eupelmus confusus Corsica
10250 Eupelmus confusus Spain
10596 Eupelmus confusus France
10426 Eupelmus confusus Greece
10507 Eupelmus pistaciae France
10005 Eupelmus pistaciae France
10004 Eupelmus pistaciae France
10041 Eupelmus annulatus Hungary
10198 Eupelmus annulatus France
10471 Eupelmus annulatus Sweden
10354 Eupelmus annulatus France
10297 Eupelmus simizonus France
10118 Eupelmus cerris Hungary
10603 Eupelmus longicalvus Italy
10019 Eupelmus longicalvus Italy
10429 Eupelmus longicalvus Sweden
10130 Eupelmus gemellus Italy
10415 Eupelmus gemellus Corsica
10405 Eupelmus gemellus Sardinia
10438 Eupelmus gemellus France
10230 Eupelmus gemellus France
10237 Eupelmus acinellus France
10235 Eupelmus acinellus France
10578 Eupelmus azureus Turkey
10034 Eupelmus azureus Italy
10361 Eupelmus azureus France
10222 Eupelmus azureus France
10575 Eupelmus azureus Hungary
10486 Eupelmus azureus Switzerland
10245 Eupelmus azureus Sardinia
10580 Eupelmus atropurpureus Spain
10192 Eupelmus microzonus Corsica
10091 Eupelmus seculatus France
10090 Eupelmus falcatus Hungary
10048 Eupelmus pini France
10318 Eupelmus matranus France
10062 Eupelmus linearis France
10066 Eupelmus linearis Hungary
10075 Eupelmus testaceiventris Cameroon
10057 Eupelmus juniperinus thuriferae France
10350 Reikosiella aff. rostrata France
10336 Reikosiella aff. rostrata France
10107 Anastatus aff. temporalis France
10105 Anastatus sidereus France

Figure S7 a)

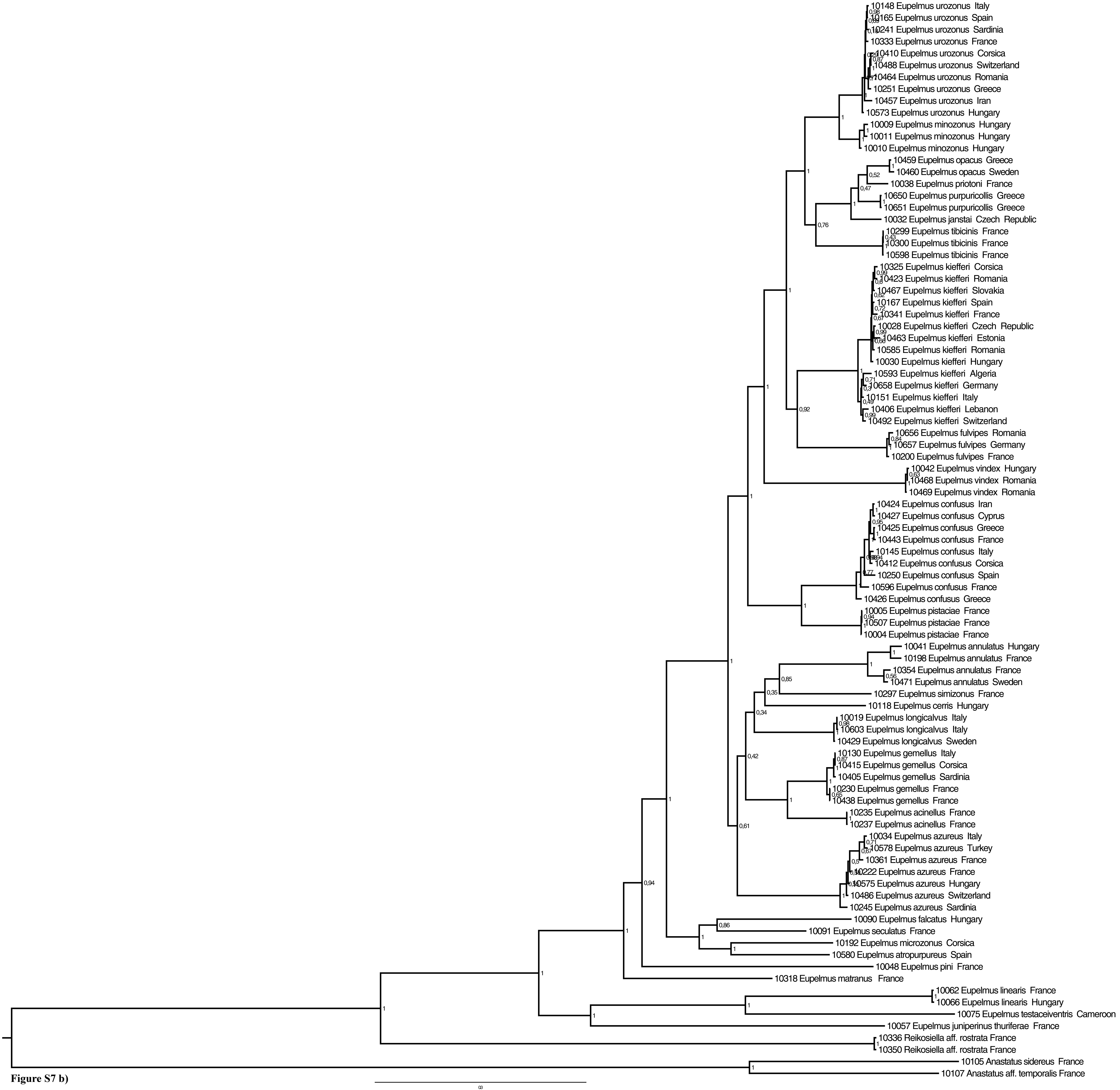
10148 Eupelmus urozonus Italy
10165 Eupelmus urozonus Spain
10241 Eupelmus urozonus Sardinia
10333 Eupelmus urozonus France
10410 Eupelmus urozonus Corsica
10488 Eupelmus urozonus Switzerland
10464 Eupelmus urozonus Romania
10251 Eupelmus urozonus Greece
10457 Eupelmus urozonus Iran
10573 Eupelmus urozonus Hungary
10009 Eupelmus minozonus Hungary
10011 Eupelmus minozonus Hungary
10010 Eupelmus minozonus Hungary
10459 Eupelmus opacus Greece
10460 Eupelmus opacus Sweden
10038 Eupelmus priotoni France
10650 Eupelmus purpuricollis Greece
10651 Eupelmus purpuricollis Greece
10032 Eupelmus janstai Czech Republic
10299 Eupelmus tibicinis France
10300 Eupelmus tibicinis France
10598 Eupelmus tibicinis France
10325 Eupelmus kiefferi Corsica
10423 Eupelmus kiefferi Romania
10467 Eupelmus kiefferi Slovakia
10167 Eupelmus kiefferi Spain
10341 Eupelmus kiefferi France
10028 Eupelmus kiefferi Czech Republic
10463 Eupelmus kiefferi Estonia
10585 Eupelmus kiefferi Romania
10030 Eupelmus kiefferi Hungary
10593 Eupelmus kiefferi Algeria
10658 Eupelmus kiefferi Germany
10151 Eupelmus kiefferi Italy
10406 Eupelmus kiefferi Lebanon
10492 Eupelmus kiefferi Switzerland
10656 Eupelmus fulvipes Romania
10657 Eupelmus fulvipes Germany
10200 Eupelmus fulvipes France
10042 Eupelmus vindex Hungary
10468 Eupelmus vindex Romania
10469 Eupelmus vindex Romania
10424 Eupelmus confusus Iran
10427 Eupelmus confusus Cyprus
10425 Eupelmus confusus Greece
10443 Eupelmus confusus France
10145 Eupelmus confusus Italy
10412 Eupelmus confusus Corsica
10250 Eupelmus confusus Spain
10596 Eupelmus confusus France
10426 Eupelmus confusus Greece
10005 Eupelmus pistaciae France
10507 Eupelmus pistaciae France
10004 Eupelmus pistaciae France
10041 Eupelmus annulatus Hungary
10198 Eupelmus annulatus France
10354 Eupelmus annulatus France
10471 Eupelmus annulatus Sweden
10297 Eupelmus simizonus France
10118 Eupelmus cerris Hungary
10019 Eupelmus longicalvus Italy
10603 Eupelmus longicalvus Italy
10429 Eupelmus longicalvus Sweden
10130 Eupelmus gemellus Italy
10415 Eupelmus gemellus Corsica
10405 Eupelmus gemellus Sardinia
10230 Eupelmus gemellus France
10438 Eupelmus gemellus France
10235 Eupelmus acinellus France
10237 Eupelmus acinellus France
10034 Eupelmus azureus Italy
10578 Eupelmus azureus Turkey
10361 Eupelmus azureus France
10222 Eupelmus azureus France
10575 Eupelmus azureus Hungary
10486 Eupelmus azureus Switzerland
10245 Eupelmus azureus Sardinia
10090 Eupelmus falcatus Hungary
10091 Eupelmus seculatus France
10192 Eupelmus microzonus Corsica
10580 Eupelmus atropurpureus Spain
10048 Eupelmus pini France
10318 Eupelmus matranus France
10062 Eupelmus linearis France
10066 Eupelmus linearis Hungary
10075 Eupelmus testaceiventris Cameroon
10057 Eupelmus juniperinus thuriferae France
10336 Reikosiella aff. rostrata France
10350 Reikosiella aff. rostrata France
10105 Anastatus sidereus France
10107 Anastatus aff. temporalis France

Figure S7 b)

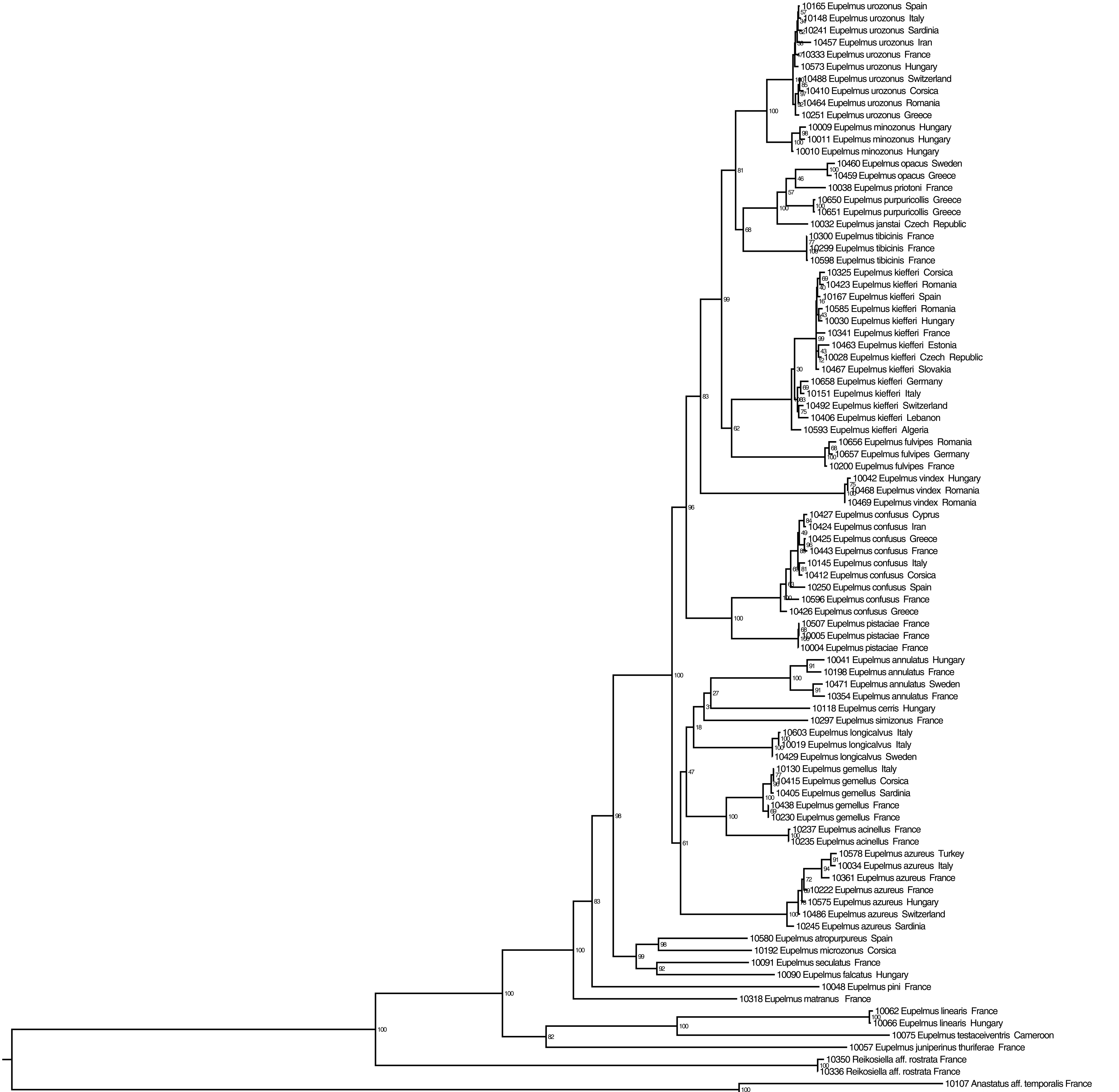
10165 Eupelmus urozonus Spain
10148 Eupelmus urozonus Italy
10241 Eupelmus urozonus Sardinia
10457 Eupelmus urozonus Iran
10333 Eupelmus urozonus France
10573 Eupelmus urozonus Hungary
10488 Eupelmus urozonus Switzerland
10410 Eupelmus urozonus Corsica
10464 Eupelmus urozonus Romania
10251 Eupelmus urozonus Greece
10009 Eupelmus minozonus Hungary
10011 Eupelmus minozonus Hungary
10010 Eupelmus minozonus Hungary
10460 Eupelmus opacus Sweden
10459 Eupelmus opacus Greece
10038 Eupelmus priotoni France
10650 Eupelmus purpuricollis Greece
10651 Eupelmus purpuricollis Greece
10032 Eupelmus janstai Czech Republic
10300 Eupelmus tibicinis France
10299 Eupelmus tibicinis France
10598 Eupelmus tibicinis France
10325 Eupelmus kiefferi Corsica
10423 Eupelmus kiefferi Romania
10167 Eupelmus kiefferi Spain
10585 Eupelmus kiefferi Romania
10030 Eupelmus kiefferi Hungary
10341 Eupelmus kiefferi France
10463 Eupelmus kiefferi Estonia
10028 Eupelmus kiefferi Czech Republic
10467 Eupelmus kiefferi Slovakia
10658 Eupelmus kiefferi Germany
10151 Eupelmus kiefferi Italy
10492 Eupelmus kiefferi Switzerland
10406 Eupelmus kiefferi Lebanon
10593 Eupelmus kiefferi Algeria
10656 Eupelmus fulvipes Romania
10657 Eupelmus fulvipes Germany
10200 Eupelmus fulvipes France
10042 Eupelmus vindex Hungary
10468 Eupelmus vindex Romania
10469 Eupelmus vindex Romania
10427 Eupelmus confusus Cyprus
10424 Eupelmus confusus Iran
10425 Eupelmus confusus Greece
10443 Eupelmus confusus France
10145 Eupelmus confusus Italy
10412 Eupelmus confusus Corsica
10250 Eupelmus confusus Spain
10596 Eupelmus confusus France
10426 Eupelmus confusus Greece
10507 Eupelmus pistaciae France
10005 Eupelmus pistaciae France
10004 Eupelmus pistaciae France
10041 Eupelmus annulatus Hungary
10198 Eupelmus annulatus France
10471 Eupelmus annulatus Sweden
10354 Eupelmus annulatus France
10118 Eupelmus cerris Hungary
10297 Eupelmus simizonus France
10603 Eupelmus longicalvus Italy
10019 Eupelmus longicalvus Italy
10429 Eupelmus longicalvus Sweden
10130 Eupelmus gemellus Italy
10415 Eupelmus gemellus Corsica
10405 Eupelmus gemellus Sardinia
10438 Eupelmus gemellus France
10230 Eupelmus gemellus France
10237 Eupelmus acinellus France
10235 Eupelmus acinellus France
10578 Eupelmus azureus Turkey
10034 Eupelmus azureus Italy
10361 Eupelmus azureus France
10222 Eupelmus azureus France
10575 Eupelmus azureus Hungary
10486 Eupelmus azureus Switzerland
10245 Eupelmus azureus Sardinia
10580 Eupelmus atropurpureus Spain
10192 Eupelmus microzonus Corsica
10091 Eupelmus seculatus France
10090 Eupelmus falcatus Hungary
10048 Eupelmus pini France
10318 Eupelmus matranus France
10062 Eupelmus linearis France
10066 Eupelmus linearis Hungary
10075 Eupelmus testaceiventris Cameroon
10057 Eupelmus juniperinus thuriferae France
10350 Reikosiella aff. rostrata France
10336 Reikosiella aff. rostrata France
10107 Anastatus aff. temporalis France
10105 Anastatus sidereus France

Figure S8 a)

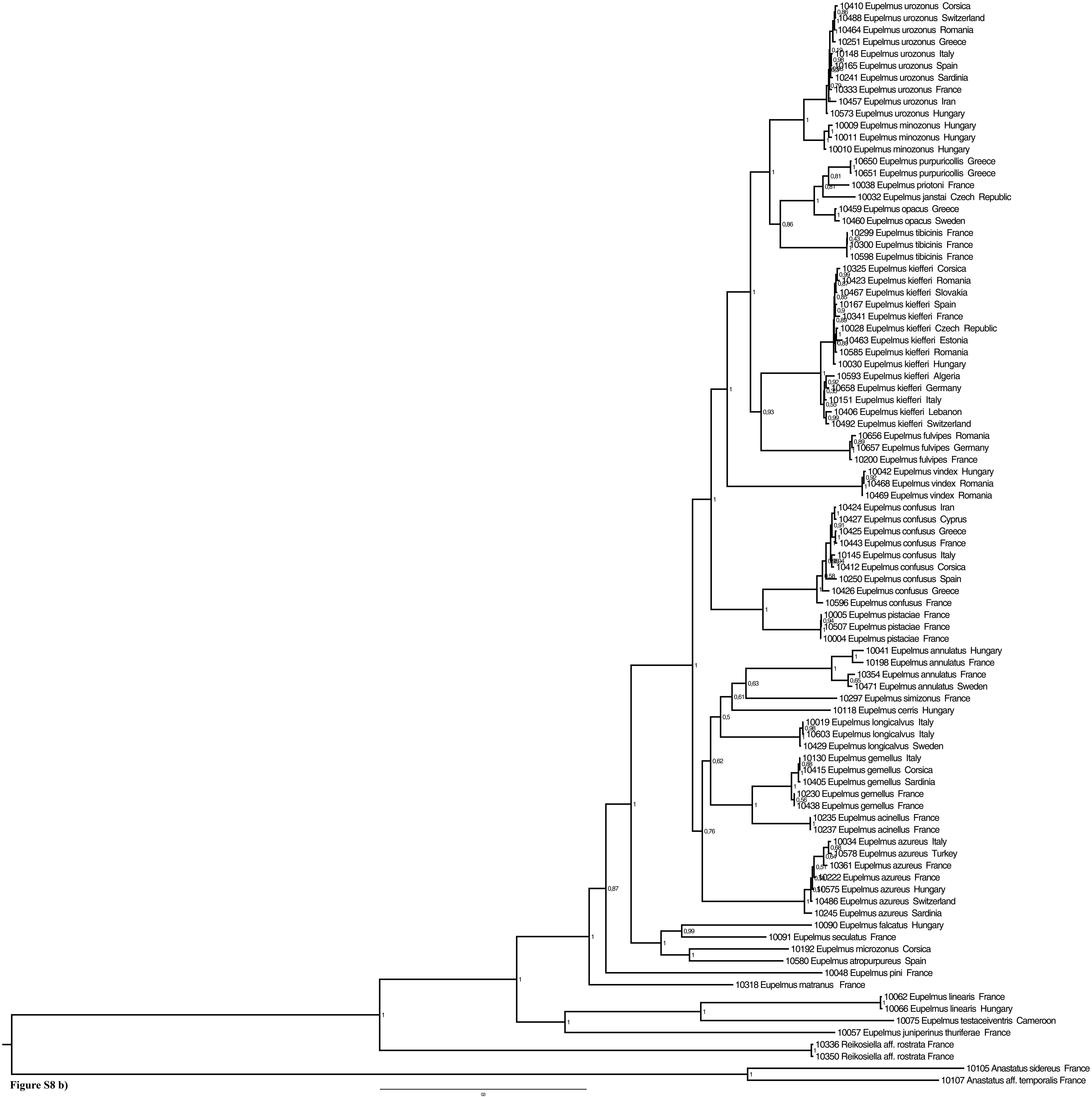
10410 Eupelmus urozonus Corsica
10488 Eupelmus urozonus Switzerland
10464 Eupelmus urozonus Romania
10251 Eupelmus urozonus Greece
10148 Eupelmus urozonus Italy
10165 Eupelmus urozonus Spain
10241 Eupelmus urozonus Sardinia
10333 Eupelmus urozonus France
10457 Eupelmus urozonus Iran
10573 Eupelmus urozonus Hungary
10009 Eupelmus minozonus Hungary
10011 Eupelmus minozonus Hungary
10010 Eupelmus minozonus Hungary
10650 Eupelmus purpuricollis Greece
10651 Eupelmus purpuricollis Greece
10038 Eupelmus priotoni France
10032 Eupelmus janstai Czech Republic
10459 Eupelmus opacus Greece
10460 Eupelmus opacus Sweden
10299 Eupelmus tibicinis France
10300 Eupelmus tibicinis France
10598 Eupelmus tibicinis France
10325 Eupelmus kiefferi Corsica
10423 Eupelmus kiefferi Romania
10467 Eupelmus kiefferi Slovakia
10167 Eupelmus kiefferi Spain
10341 Eupelmus kiefferi France
10028 Eupelmus kiefferi Czech Republic
10463 Eupelmus kiefferi Estonia
10585 Eupelmus kiefferi Romania
10030 Eupelmus kiefferi Hungary
10593 Eupelmus kiefferi Algeria
10658 Eupelmus kiefferi Germany
10151 Eupelmus kiefferi Italy
10406 Eupelmus kiefferi Lebanon
10492 Eupelmus kiefferi Switzerland
10656 Eupelmus fulvipes Romania
10657 Eupelmus fulvipes Germany
10200 Eupelmus fulvipes France
10042 Eupelmus vindex Hungary
10468 Eupelmus vindex Romania
10469 Eupelmus vindex Romania
10424 Eupelmus confusus Iran
10427 Eupelmus confusus Cyprus
10425 Eupelmus confusus Greece
10443 Eupelmus confusus France
10145 Eupelmus confusus Italy
10412 Eupelmus confusus Corsica
10250 Eupelmus confusus Spain
10426 Eupelmus confusus Greece
10596 Eupelmus confusus France
10005 Eupelmus pistaciae France
10507 Eupelmus pistaciae France
10004 Eupelmus pistaciae France
10041 Eupelmus annulatus Hungary
10198 Eupelmus annulatus France
10354 Eupelmus annulatus France
10471 Eupelmus annulatus Sweden
10297 Eupelmus simizonus France
10118 Eupelmus cerris Hungary
10019 Eupelmus longicalvus Italy
10603 Eupelmus longicalvus Italy
10429 Eupelmus longicalvus Sweden
10130 Eupelmus gemellus Italy
10415 Eupelmus gemellus Corsica
10405 Eupelmus gemellus Sardinia
10230 Eupelmus gemellus France
10438 Eupelmus gemellus France
10235 Eupelmus acinellus France
10237 Eupelmus acinellus France
10034 Eupelmus azureus Italy
10578 Eupelmus azureus Turkey
10361 Eupelmus azureus France
10222 Eupelmus azureus France
10575 Eupelmus azureus Hungary
10486 Eupelmus azureus Switzerland
10245 Eupelmus azureus Sardinia
10090 Eupelmus falcatus Hungary
10091 Eupelmus seculatus France
10192 Eupelmus microzonus Corsica
10580 Eupelmus atropurpureus Spain
10048 Eupelmus pini France
10318 Eupelmus matranus France
10062 Eupelmus linearis France
10066 Eupelmus linearis Hungary
10075 Eupelmus testaceiventris Cameroon
10057 Eupelmus juniperinus thuriferae France
10336 Reikosiella aff. rostrata France
10350 Reikosiella aff. rostrata France
10105 Anastatus sidereus France
10107 Anastatus aff. temporalis France

Figure S8 b)

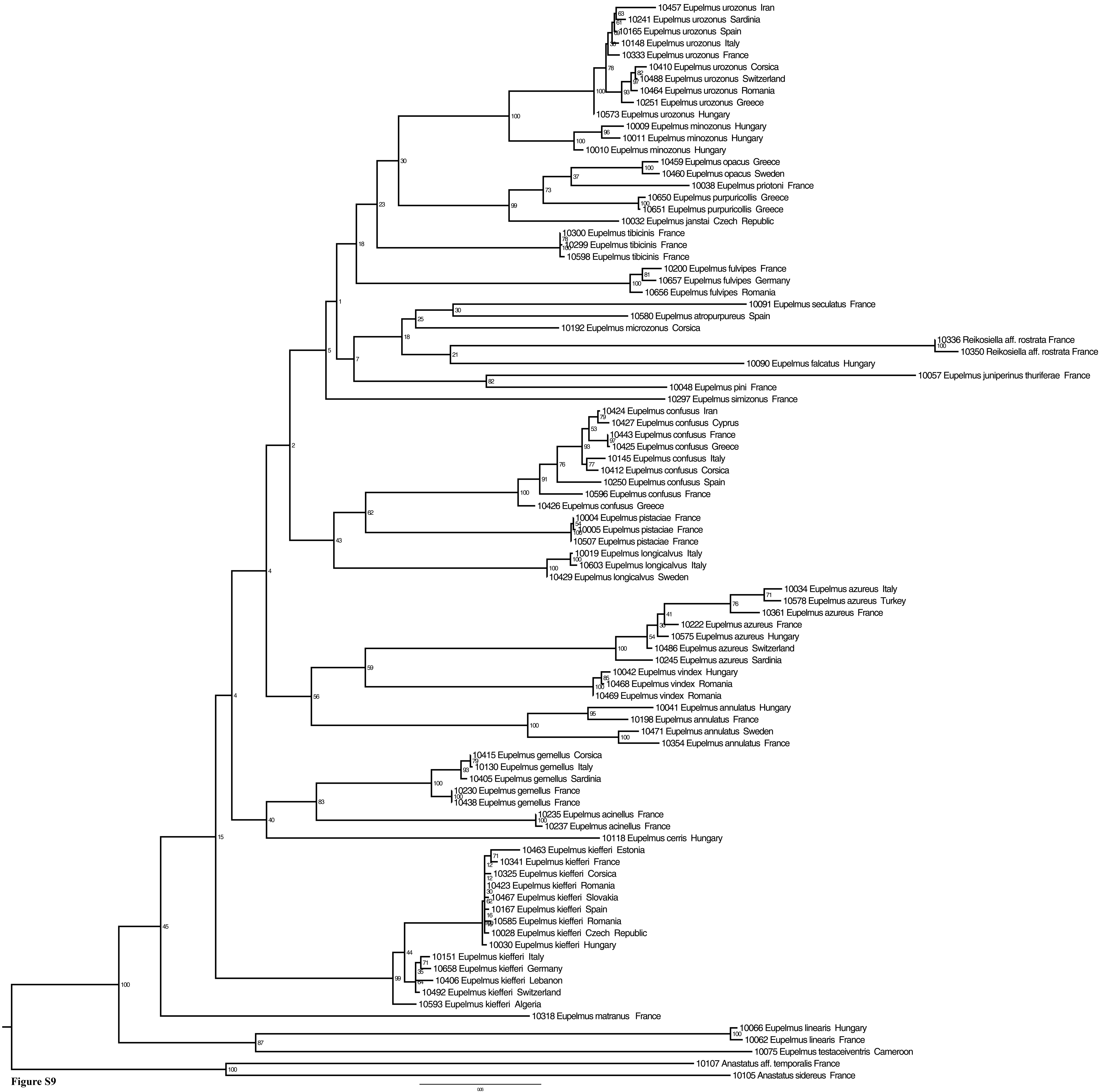
10457 Eupelmus urozonus Iran
10241 Eupelmus urozonus Sardinia
10165 Eupelmus urozonus Spain
10148 Eupelmus urozonus Italy
10333 Eupelmus urozonus France
10410 Eupelmus urozonus Corsica
10488 Eupelmus urozonus Switzerland
10464 Eupelmus urozonus Romania
10251 Eupelmus urozonus Greece
10573 Eupelmus urozonus Hungary
10009 Eupelmus minozonus Hungary
10011 Eupelmus minozonus Hungary
10010 Eupelmus minozonus Hungary
10459 Eupelmus opacus Greece
10460 Eupelmus opacus Sweden
10038 Eupelmus priotoni France
10650 Eupelmus purpuricollis Greece
10651 Eupelmus purpuricollis Greece
10032 Eupelmus janstai Czech Republic
10300 Eupelmus tibicinis France
10299 Eupelmus tibicinis France
10598 Eupelmus tibicinis France
10200 Eupelmus fulvipes France
10657 Eupelmus fulvipes Germany
10656 Eupelmus fulvipes Romania
10091 Eupelmus seculatus France
10580 Eupelmus atropurpureus Spain
10192 Eupelmus microzonus Corsica
10336 Reikosiella aff. rostrata France
10350 Reikosiella aff. rostrata France
10090 Eupelmus falcatus Hungary
10057 Eupelmus juniperinus thuriferae France
10048 Eupelmus pini France
10297 Eupelmus simizonus France
10424 Eupelmus confusus Iran
10427 Eupelmus confusus Cyprus
10443 Eupelmus confusus France
10425 Eupelmus confusus Greece
10145 Eupelmus confusus Italy
10412 Eupelmus confusus Corsica
10250 Eupelmus confusus Spain
10596 Eupelmus confusus France
10426 Eupelmus confusus Greece
10004 Eupelmus pistaciae France
10005 Eupelmus pistaciae France
10507 Eupelmus pistaciae France
10019 Eupelmus longicalvus Italy
10603 Eupelmus longicalvus Italy
10429 Eupelmus longicalvus Sweden
10034 Eupelmus azureus Italy
10578 Eupelmus azureus Turkey
10361 Eupelmus azureus France
10222 Eupelmus azureus France
10575 Eupelmus azureus Hungary
10486 Eupelmus azureus Switzerland
10245 Eupelmus azureus Sardinia
10042 Eupelmus vindex Hungary
10468 Eupelmus vindex Romania
10469 Eupelmus vindex Romania
10041 Eupelmus annulatus Hungary
10198 Eupelmus annulatus France
10471 Eupelmus annulatus Sweden
10354 Eupelmus annulatus France
10415 Eupelmus gemellus Corsica
10130 Eupelmus gemellus Italy
10405 Eupelmus gemellus Sardinia
10230 Eupelmus gemellus France
10438 Eupelmus gemellus France
10235 Eupelmus acinellus France
10237 Eupelmus acinellus France
10118 Eupelmus cerris Hungary
10463 Eupelmus kiefferi Estonia
10341 Eupelmus kiefferi France
10325 Eupelmus kiefferi Corsica
10423 Eupelmus kiefferi Romania
10467 Eupelmus kiefferi Slovakia
10167 Eupelmus kiefferi Spain
10585 Eupelmus kiefferi Romania
10028 Eupelmus kiefferi Czech Republic
10030 Eupelmus kiefferi Hungary
10151 Eupelmus kiefferi Italy
10658 Eupelmus kiefferi Germany
10406 Eupelmus kiefferi Lebanon
10492 Eupelmus kiefferi Switzerland
10593 Eupelmus kiefferi Algeria
10318 Eupelmus matranus France
10066 Eupelmus linearis Hungary
10062 Eupelmus linearis France
10075 Eupelmus testaceiventris Cameroon
10107 Anastatus aff. temporalis France
10105 Anastatus sidereus France

Figure S9

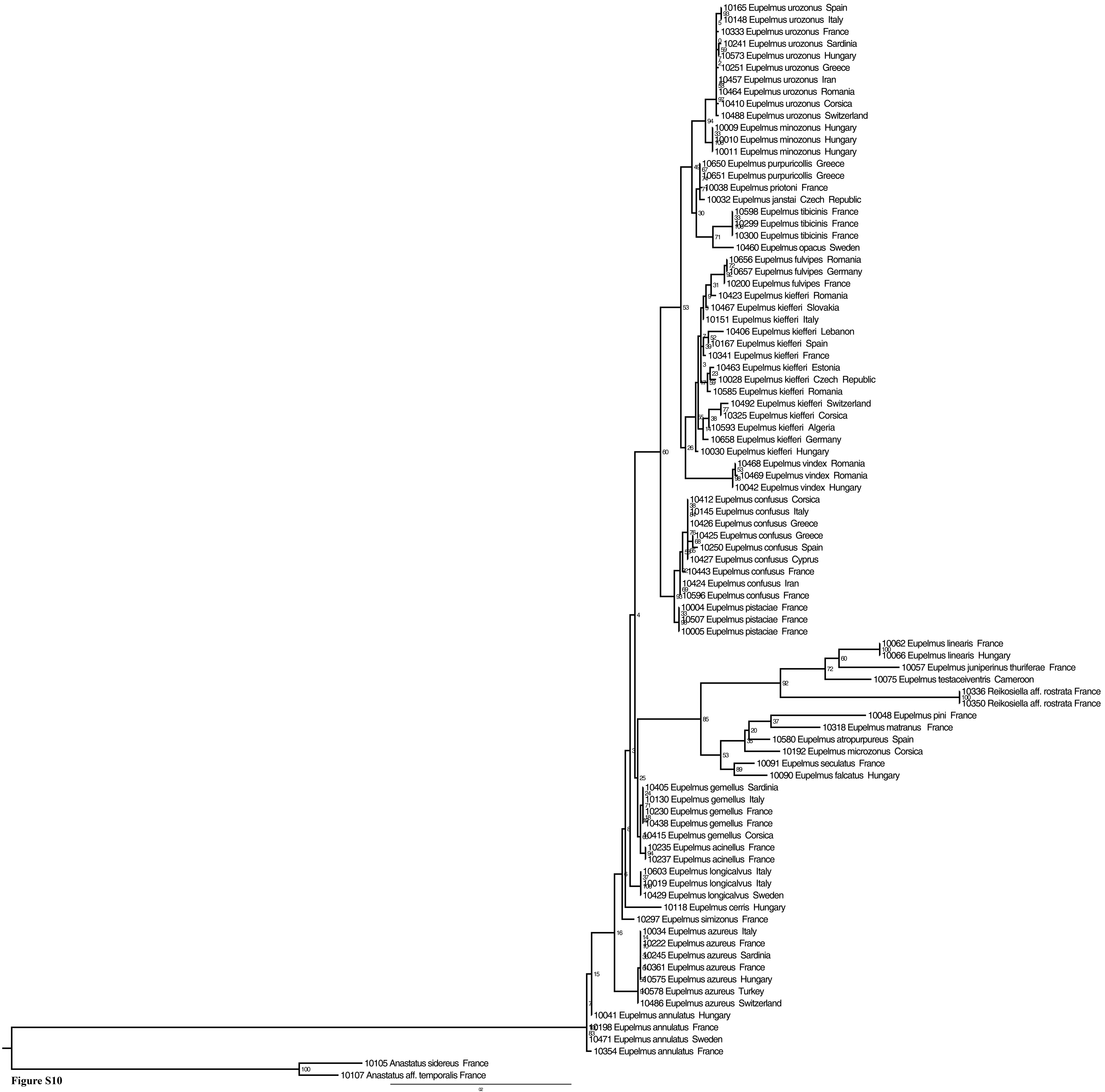

10165 Eupelmus urozonus Spain
10148 Eupelmus urozonus Italy
10333 Eupelmus urozonus France
10241 Eupelmus urozonus Sardinia
10573 Eupelmus urozonus Hungary
10251 Eupelmus urozonus Greece
10457 Eupelmus urozonus Iran
10464 Eupelmus urozonus Romania
10410 Eupelmus urozonus Corsica
10488 Eupelmus urozonus Switzerland
10009 Eupelmus minozonus Hungary
10010 Eupelmus minozonus Hungary
10011 Eupelmus minozonus Hungary
10650 Eupelmus purpuricollis Greece
10651 Eupelmus purpuricollis Greece
10038 Eupelmus priotoni France
10032 Eupelmus janstai Czech Republic
10598 Eupelmus tibicinis France
10299 Eupelmus tibicinis France
10300 Eupelmus tibicinis France
10460 Eupelmus opacus Sweden
10656 Eupelmus fulvipes Romania
10657 Eupelmus fulvipes Germany
10200 Eupelmus fulvipes France
10423 Eupelmus kiefferi Romania
10467 Eupelmus kiefferi Slovakia
10151 Eupelmus kiefferi Italy
10406 Eupelmus kiefferi Lebanon
10167 Eupelmus kiefferi Spain
10341 Eupelmus kiefferi France
10463 Eupelmus kiefferi Estonia
10028 Eupelmus kiefferi Czech Republic
10585 Eupelmus kiefferi Romania
10492 Eupelmus kiefferi Switzerland
10325 Eupelmus kiefferi Corsica
10593 Eupelmus kiefferi Algeria
10658 Eupelmus kiefferi Germany
10030 Eupelmus kiefferi Hungary
10468 Eupelmus vindex Romania
10469 Eupelmus vindex Romania
10042 Eupelmus vindex Hungary
10412 Eupelmus confusus Corsica
10145 Eupelmus confusus Italy
10426 Eupelmus confusus Greece
10425 Eupelmus confusus Greece
10250 Eupelmus confusus Spain
10427 Eupelmus confusus Cyprus
10443 Eupelmus confusus France
10424 Eupelmus confusus Iran
10596 Eupelmus confusus France
10004 Eupelmus pistaciae France
10507 Eupelmus pistaciae France
10005 Eupelmus pistaciae France
10062 Eupelmus linearis France
10066 Eupelmus linearis Hungary
10057 Eupelmus juniperinus thuriferae France
10075 Eupelmus testaceiventris Cameroon
10336 Reikosiella aff. rostrata France
10350 Reikosiella aff. rostrata France
10048 Eupelmus pini France
10318 Eupelmus matranus France
10580 Eupelmus atropurpureus Spain
10192 Eupelmus microzonus Corsica
10091 Eupelmus seculatus France
10090 Eupelmus falcatus Hungary
10405 Eupelmus gemellus Sardinia
10130 Eupelmus gemellus Italy
10230 Eupelmus gemellus France
10438 Eupelmus gemellus France
10415 Eupelmus gemellus Corsica
10235 Eupelmus acinellus France
10237 Eupelmus acinellus France
10603 Eupelmus longicalvus Italy
10019 Eupelmus longicalvus Italy
10429 Eupelmus longicalvus Sweden
10118 Eupelmus cerris Hungary
10297 Eupelmus simizonus France
10034 Eupelmus azureus Italy
10222 Eupelmus azureus France
10245 Eupelmus azureus Sardinia
10361 Eupelmus azureus France
10575 Eupelmus azureus Hungary
10578 Eupelmus azureus Turkey
10486 Eupelmus azureus Switzerland
10041 Eupelmus annulatus Hungary
10198 Eupelmus annulatus France
10471 Eupelmus annulatus Sweden
10354 Eupelmus annulatus France
10105 Anastatus sidereus France
10107 Anastatus aff. temporalis France

Figure S10

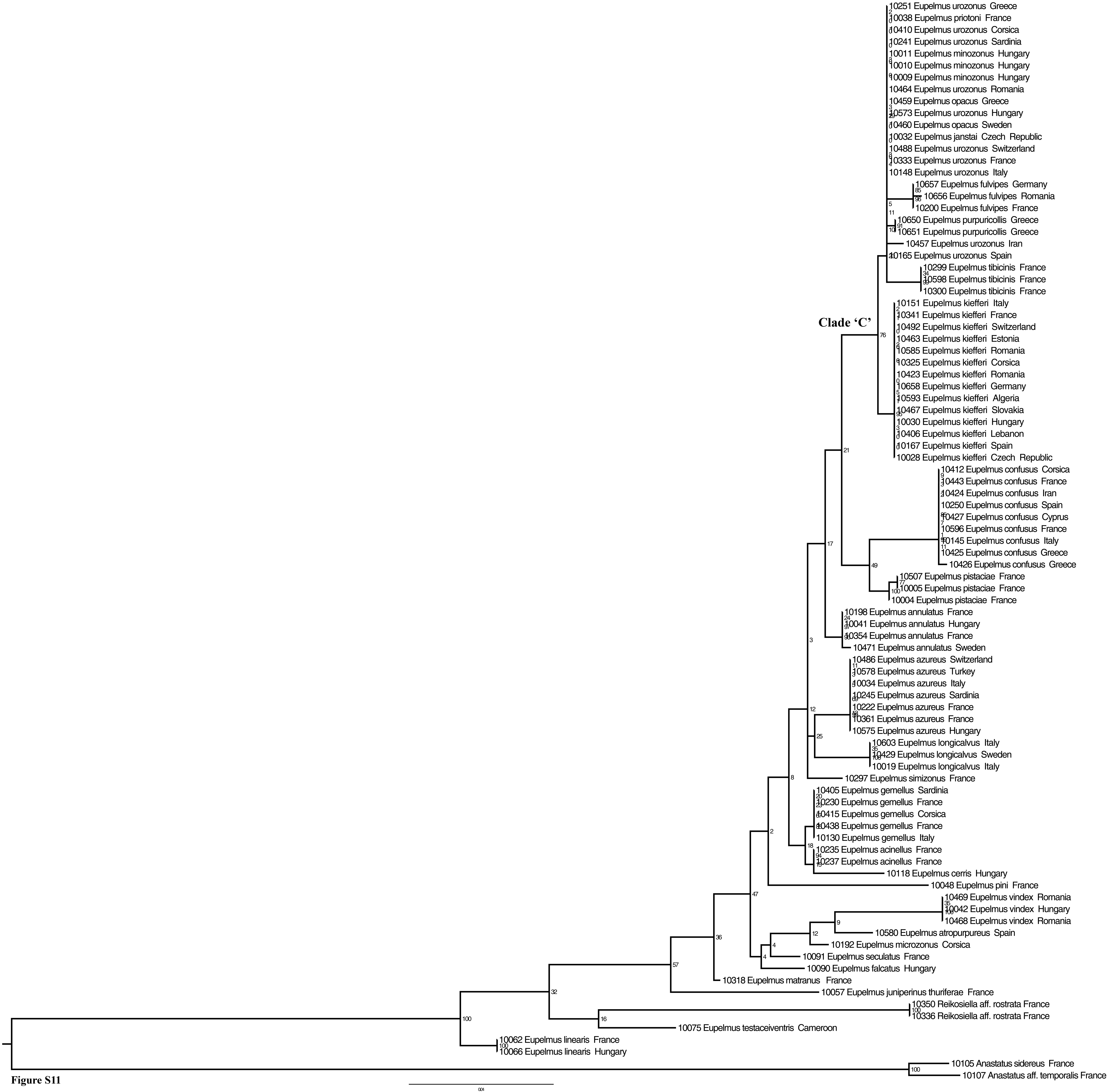
10251 Eupelmus urozonus Greece
10038 Eupelmus priotoni France
10410 Eupelmus urozonus Corsica
10241 Eupelmus urozonus Sardinia
10011 Eupelmus minozonus Hungary
10010 Eupelmus minozonus Hungary
10009 Eupelmus minozonus Hungary
10464 Eupelmus urozonus Romania
10459 Eupelmus opacus Greece
10573 Eupelmus urozonus Hungary
10460 Eupelmus opacus Sweden
10032 Eupelmus janstai Czech Republic
10488 Eupelmus urozonus Switzerland
10333 Eupelmus urozonus France
10148 Eupelmus urozonus Italy
10657 Eupelmus fulvipes Germany
10656 Eupelmus fulvipes Romania
10200 Eupelmus fulvipes France
10650 Eupelmus purpuricollis Greece
10651 Eupelmus purpuricollis Greece
10457 Eupelmus urozonus Iran
10165 Eupelmus urozonus Spain
10299 Eupelmus tibicinis France
10598 Eupelmus tibicinis France
10300 Eupelmus tibicinis France
10151 Eupelmus kiefferi Italy
Clade 'C'
10341 Eupelmus kiefferi France
10492 Eupelmus kiefferi Switzerland
10463 Eupelmus kiefferi Estonia
10585 Eupelmus kiefferi Romania
10325 Eupelmus kiefferi Corsica
10423 Eupelmus kiefferi Romania
10658 Eupelmus kiefferi Germany
10593 Eupelmus kiefferi Algeria
10467 Eupelmus kiefferi Slovakia
10030 Eupelmus kiefferi Hungary
10406 Eupelmus kiefferi Lebanon
10167 Eupelmus kiefferi Spain
10028 Eupelmus kiefferi Czech Republic
10412 Eupelmus confusus Corsica
10443 Eupelmus confusus France
10424 Eupelmus confusus Iran
10250 Eupelmus confusus Spain
10427 Eupelmus confusus Cyprus
10596 Eupelmus confusus France
10145 Eupelmus confusus Italy
10425 Eupelmus confusus Greece
10426 Eupelmus confusus Greece
10507 Eupelmus pistaciae France
10005 Eupelmus pistaciae France
10004 Eupelmus pistaciae France
10198 Eupelmus annulatus France
10041 Eupelmus annulatus Hungary
10354 Eupelmus annulatus France
10471 Eupelmus annulatus Sweden
10486 Eupelmus azureus Switzerland
10578 Eupelmus azureus Turkey
10034 Eupelmus azureus Italy
10245 Eupelmus azureus Sardinia
10222 Eupelmus azureus France
10361 Eupelmus azureus France
10575 Eupelmus azureus Hungary
10603 Eupelmus longicalvus Italy
10429 Eupelmus longicalvus Sweden
10019 Eupelmus longicalvus Italy
10297 Eupelmus simizonus France
10405 Eupelmus gemellus Sardinia
10230 Eupelmus gemellus France
10415 Eupelmus gemellus Corsica
10438 Eupelmus gemellus France
10130 Eupelmus gemellus Italy
10235 Eupelmus acinellus France
10237 Eupelmus acinellus France
10118 Eupelmus cerris Hungary
10048 Eupelmus pini France
10469 Eupelmus vindex Romania
10042 Eupelmus vindex Hungary
10468 Eupelmus vindex Romania
10580 Eupelmus atropurpureus Spain
10192 Eupelmus microzonus Corsica
10091 Eupelmus seculatus France
10090 Eupelmus falcatus Hungary
10318 Eupelmus matranus France
10057 Eupelmus juniperinus thuriferae France
10350 Reikosiella aff. rostrata France
10336 Reikosiella aff. rostrata France
10075 Eupelmus testaceiventris Cameroon
10062 Eupelmus linearis France
10066 Eupelmus linearis Hungary
10105 Anastatus sidereus France
10107 Anastatus aff. temporalis France

Figure S11

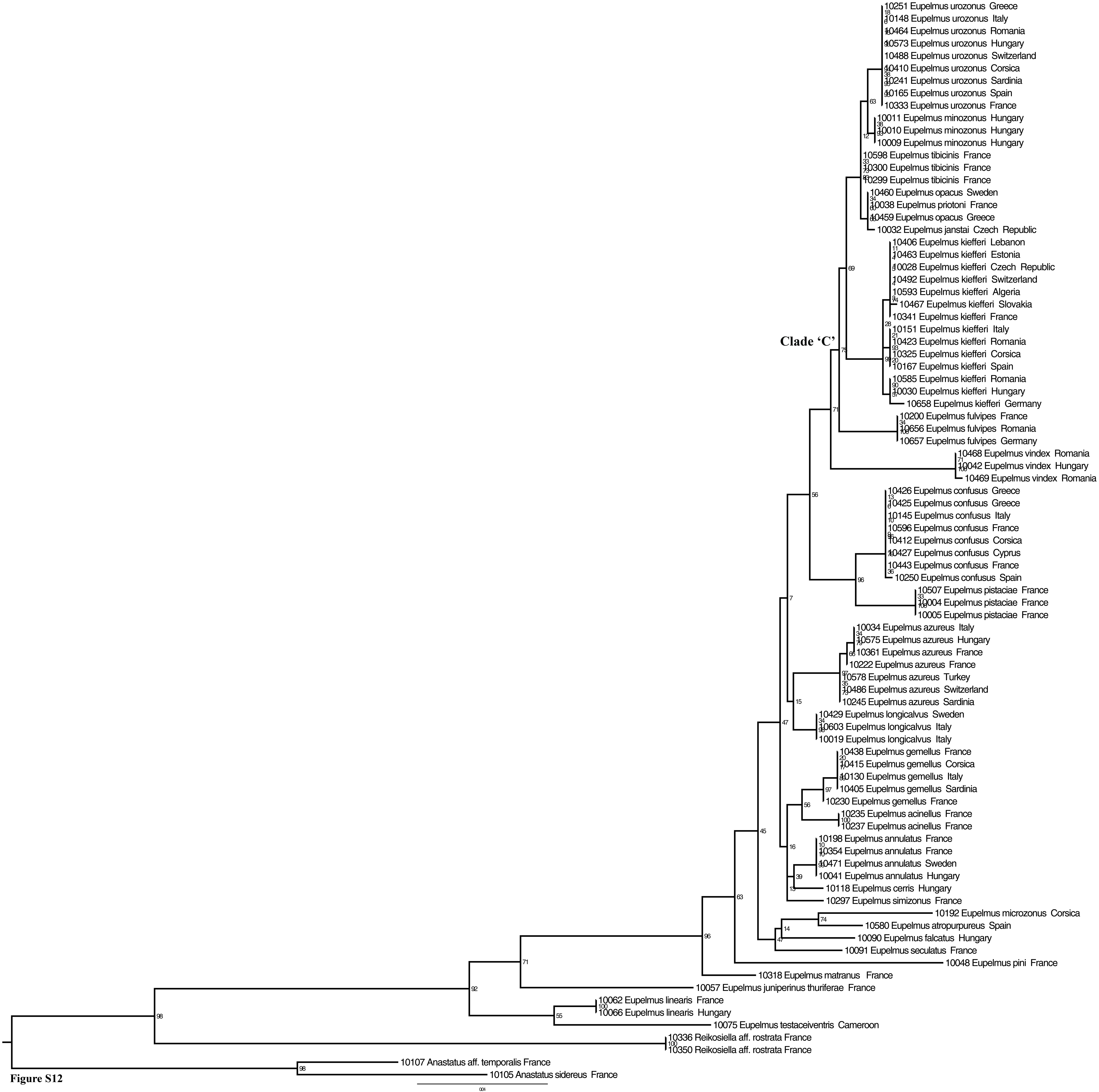

10251 Eupelmus urozonus Greece
10148 Eupelmus urozonus Italy
10464 Eupelmus urozonus Romania
10573 Eupelmus urozonus Hungary
10488 Eupelmus urozonus Switzerland
10410 Eupelmus urozonus Corsica
10241 Eupelmus urozonus Sardinia
10165 Eupelmus urozonus Spain
10333 Eupelmus urozonus France
10011 Eupelmus minozonus Hungary
10010 Eupelmus minozonus Hungary
10009 Eupelmus minozonus Hungary
10598 Eupelmus tibicinis France
10300 Eupelmus tibicinis France
10299 Eupelmus tibicinis France
10460 Eupelmus opacus Sweden
10038 Eupelmus priotoni France
10459 Eupelmus opacus Greece
10032 Eupelmus janstai Czech Republic
10406 Eupelmus kiefferi Lebanon
10463 Eupelmus kiefferi Estonia
10028 Eupelmus kiefferi Czech Republic
10492 Eupelmus kiefferi Switzerland
10593 Eupelmus kiefferi Algeria
10467 Eupelmus kiefferi Slovakia
10341 Eupelmus kiefferi France
10151 Eupelmus kiefferi Italy
10423 Eupelmus kiefferi Romania
10325 Eupelmus kiefferi Corsica
10167 Eupelmus kiefferi Spain
10585 Eupelmus kiefferi Romania
10030 Eupelmus kiefferi Hungary
10658 Eupelmus kiefferi Germany
Clade 'C'
10200 Eupelmus fulvipes France
10656 Eupelmus fulvipes Romania
10657 Eupelmus fulvipes Germany
10468 Eupelmus vindex Romania
10042 Eupelmus vindex Hungary
10469 Eupelmus vindex Romania
10426 Eupelmus confusus Greece
10425 Eupelmus confusus Greece
10145 Eupelmus confusus Italy
10596 Eupelmus confusus France
10412 Eupelmus confusus Corsica
10427 Eupelmus confusus Cyprus
10443 Eupelmus confusus France
10250 Eupelmus confusus Spain
10507 Eupelmus pistaciae France
10004 Eupelmus pistaciae France
10005 Eupelmus pistaciae France
10034 Eupelmus azureus Italy
10575 Eupelmus azureus Hungary
10361 Eupelmus azureus France
10222 Eupelmus azureus France
10578 Eupelmus azureus Turkey
10486 Eupelmus azureus Switzerland
10245 Eupelmus azureus Sardinia
10429 Eupelmus longicalvus Sweden
10603 Eupelmus longicalvus Italy
10019 Eupelmus longicalvus Italy
10438 Eupelmus gemellus France
10415 Eupelmus gemellus Corsica
10130 Eupelmus gemellus Italy
10405 Eupelmus gemellus Sardinia
10230 Eupelmus gemellus France
10235 Eupelmus acinellus France
10237 Eupelmus acinellus France
10198 Eupelmus annulatus France
10354 Eupelmus annulatus France
10471 Eupelmus annulatus Sweden
10041 Eupelmus annulatus Hungary
10118 Eupelmus cerris Hungary
10297 Eupelmus simizonus France
10192 Eupelmus microzonus Corsica
10580 Eupelmus atropurpureus Spain
10090 Eupelmus falcatus Hungary
10091 Eupelmus seculatus France
10048 Eupelmus pini France
10318 Eupelmus matranus France
10057 Eupelmus juniperinus thuriferae France
10062 Eupelmus linearis France
10066 Eupelmus linearis Hungary
10075 Eupelmus testaceiventris Cameroon
10336 Reikosiella aff. rostrata France
10350 Reikosiella aff. rostrata France
10107 Anastatus aff. temporalis France
10105 Anastatus sidereus France

Figure S12

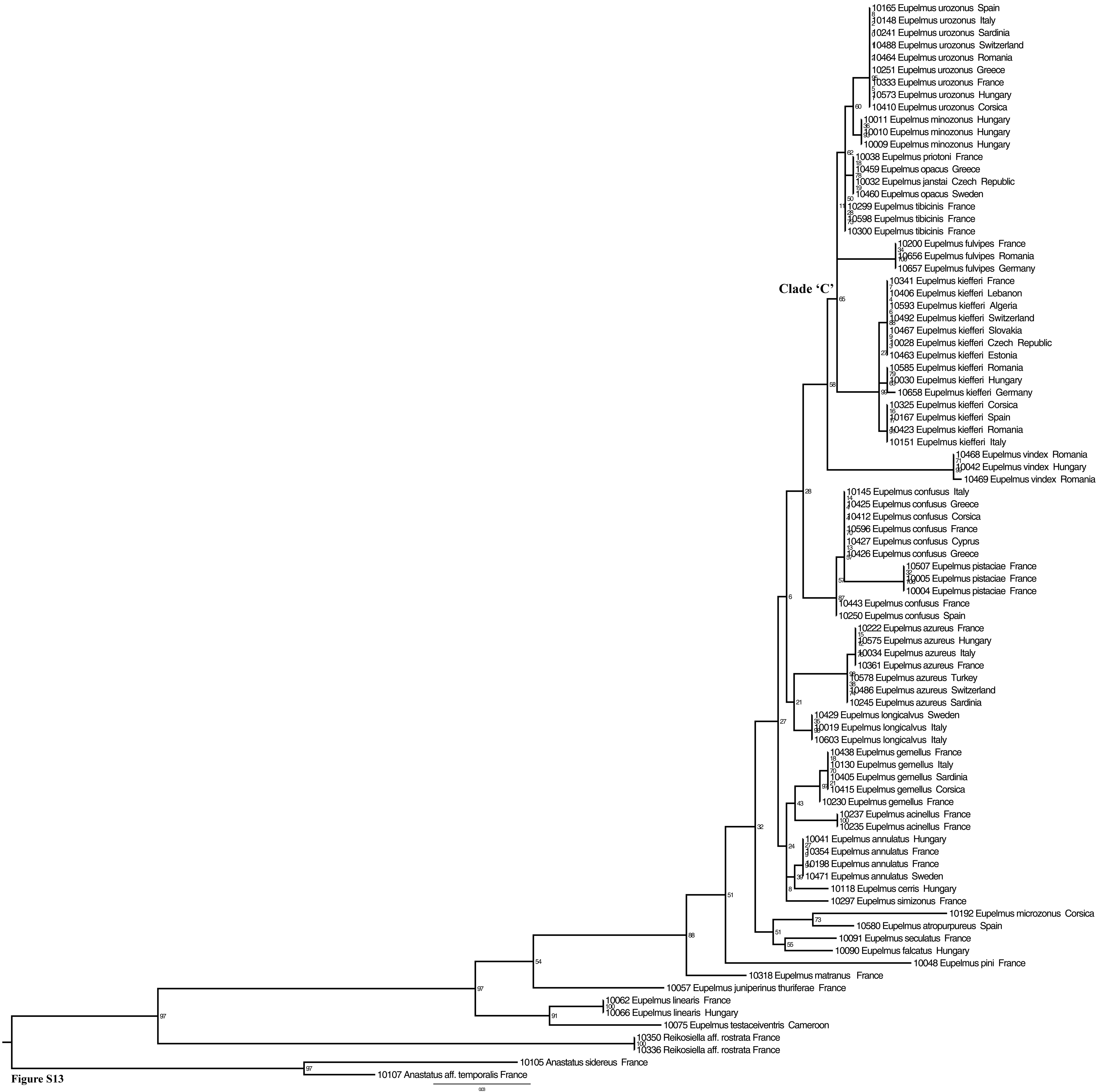

10165 Eupelmus urozonus Spain
10148 Eupelmus urozonus Italy
10241 Eupelmus urozonus Sardinia
10488 Eupelmus urozonus Switzerland
10464 Eupelmus urozonus Romania
10251 Eupelmus urozonus Greece
10333 Eupelmus urozonus France
10573 Eupelmus urozonus Hungary
10410 Eupelmus urozonus Corsica
10011 Eupelmus minozonus Hungary
10010 Eupelmus minozonus Hungary
10009 Eupelmus minozonus Hungary
10038 Eupelmus priotoni France
10459 Eupelmus opacus Greece
10032 Eupelmus janstai Czech Republic
10460 Eupelmus opacus Sweden
10299 Eupelmus tibicinis France
10598 Eupelmus tibicinis France
10300 Eupelmus tibicinis France
10200 Eupelmus fulvipes France
10656 Eupelmus fulvipes Romania
10657 Eupelmus fulvipes Germany
Clade ‘C’
10341 Eupelmus kiefferi France
10406 Eupelmus kiefferi Lebanon
10593 Eupelmus kiefferi Algeria
10492 Eupelmus kiefferi Switzerland
10467 Eupelmus kiefferi Slovakia
10028 Eupelmus kiefferi Czech Republic
10463 Eupelmus kiefferi Estonia
10585 Eupelmus kiefferi Romania
10030 Eupelmus kiefferi Hungary
10658 Eupelmus kiefferi Germany
10325 Eupelmus kiefferi Corsica
10167 Eupelmus kiefferi Spain
10423 Eupelmus kiefferi Romania
10151 Eupelmus kiefferi Italy
10468 Eupelmus vindex Romania
10042 Eupelmus vindex Hungary
10469 Eupelmus vindex Romania
10145 Eupelmus confusus Italy
10425 Eupelmus confusus Greece
10412 Eupelmus confusus Corsica
10596 Eupelmus confusus France
10427 Eupelmus confusus Cyprus
10426 Eupelmus confusus Greece
10507 Eupelmus pistaciae France
10005 Eupelmus pistaciae France
10004 Eupelmus pistaciae France
10443 Eupelmus confusus France
10250 Eupelmus confusus Spain
10222 Eupelmus azureus France
10575 Eupelmus azureus Hungary
10034 Eupelmus azureus Italy
10361 Eupelmus azureus France
10578 Eupelmus azureus Turkey
10486 Eupelmus azureus Switzerland
10245 Eupelmus azureus Sardinia
10429 Eupelmus longicalvus Sweden
10019 Eupelmus longicalvus Italy
10603 Eupelmus longicalvus Italy
10438 Eupelmus gemellus France
10130 Eupelmus gemellus Italy
10405 Eupelmus gemellus Sardinia
10415 Eupelmus gemellus Corsica
10230 Eupelmus gemellus France
10237 Eupelmus acinellus France
10235 Eupelmus acinellus France
10041 Eupelmus annulatus Hungary
10354 Eupelmus annulatus France
10198 Eupelmus annulatus France
10471 Eupelmus annulatus Sweden
10118 Eupelmus cerris Hungary
10297 Eupelmus simizonus France
10192 Eupelmus microzonus Corsica
10580 Eupelmus atropurpureus Spain
10091 Eupelmus seculatus France
10090 Eupelmus falcatus Hungary
10048 Eupelmus pini France
10318 Eupelmus matranus France
10057 Eupelmus juniperinus thuriferae France
10062 Eupelmus linearis France
10066 Eupelmus linearis Hungary
10075 Eupelmus testaceiventris Cameroon
10350 Reikosiella aff. rostrata France
10336 Reikosiella aff. rostrata France
10105 Anastatus sidereus France
10107 Anastatus aff. temporalis France

Figure S13

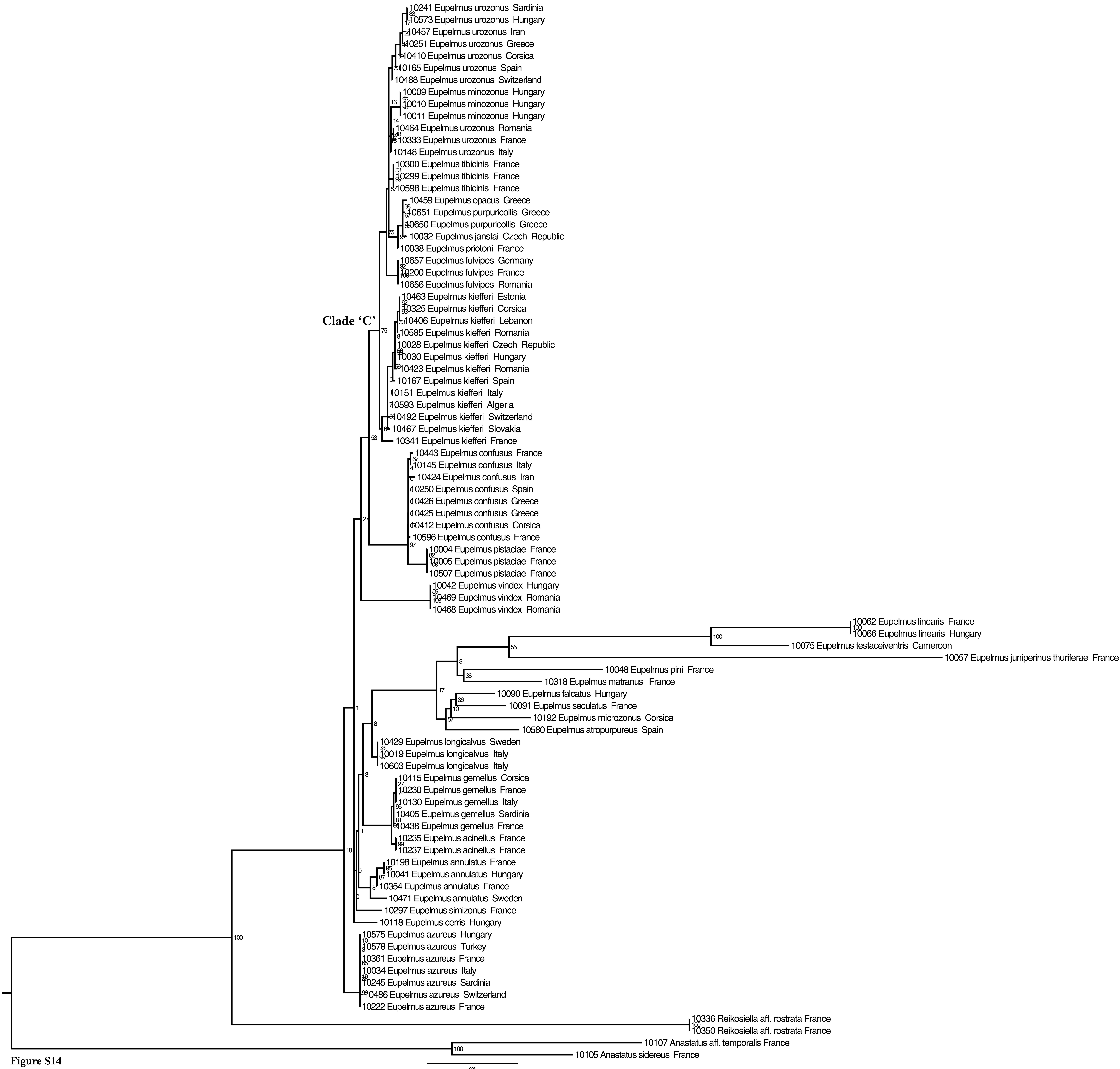
Clade 'C'
10241 Eupelmus urozonus Sardinia
10573 Eupelmus urozonus Hungary
10457 Eupelmus urozonus Iran
10251 Eupelmus urozonus Greece
10410 Eupelmus urozonus Corsica
10165 Eupelmus urozonus Spain
10488 Eupelmus urozonus Switzerland
10009 Eupelmus minozonus Hungary
10010 Eupelmus minozonus Hungary
10011 Eupelmus minozonus Hungary
10464 Eupelmus urozonus Romania
10333 Eupelmus urozonus France
10148 Eupelmus urozonus Italy
10300 Eupelmus tibicinis France
10299 Eupelmus tibicinis France
10598 Eupelmus tibicinis France
10459 Eupelmus opacus Greece
10651 Eupelmus purpuricollis Greece
10650 Eupelmus purpuricollis Greece
10032 Eupelmus janstai Czech Republic
10038 Eupelmus priotoni France
10657 Eupelmus fulvipes Germany
10200 Eupelmus fulvipes France
10656 Eupelmus fulvipes Romania
10463 Eupelmus kiefferi Estonia
10325 Eupelmus kiefferi Corsica
10406 Eupelmus kiefferi Lebanon
10585 Eupelmus kiefferi Romania
10028 Eupelmus kiefferi Czech Republic
10030 Eupelmus kiefferi Hungary
10423 Eupelmus kiefferi Romania
10167 Eupelmus kiefferi Spain
10151 Eupelmus kiefferi Italy
10593 Eupelmus kiefferi Algeria
10492 Eupelmus kiefferi Switzerland
10467 Eupelmus kiefferi Slovakia
10341 Eupelmus kiefferi France
10443 Eupelmus confusus France
10145 Eupelmus confusus Italy
10424 Eupelmus confusus Iran
10250 Eupelmus confusus Spain
10426 Eupelmus confusus Greece
10425 Eupelmus confusus Greece
10412 Eupelmus confusus Corsica
10596 Eupelmus confusus France
10004 Eupelmus pistaciae France
10005 Eupelmus pistaciae France
10507 Eupelmus pistaciae France
10042 Eupelmus vindex Hungary
10469 Eupelmus vindex Romania
10468 Eupelmus vindex Romania
10062 Eupelmus linearis France
10066 Eupelmus linearis Hungary
10075 Eupelmus testaceiventris Cameroon
10057 Eupelmus juniperinus thuriferae France
10048 Eupelmus pini France
10318 Eupelmus matranus France
10090 Eupelmus falcatus Hungary
10091 Eupelmus seculatus France
10192 Eupelmus microzonus Corsica
10580 Eupelmus atropurpureus Spain
10429 Eupelmus longicalvus Sweden
10019 Eupelmus longicalvus Italy
10603 Eupelmus longicalvus Italy
10415 Eupelmus gemellus Corsica
10230 Eupelmus gemellus France
10130 Eupelmus gemellus Italy
10405 Eupelmus gemellus Sardinia
10438 Eupelmus gemellus France
10235 Eupelmus acinellus France
10237 Eupelmus acinellus France
10198 Eupelmus annulatus France
10041 Eupelmus annulatus Hungary
10354 Eupelmus annulatus France
10471 Eupelmus annulatus Sweden
10297 Eupelmus simizonus France
10118 Eupelmus cerris Hungary
10575 Eupelmus azureus Hungary
10578 Eupelmus azureus Turkey
10361 Eupelmus azureus France
10034 Eupelmus azureus Italy
10245 Eupelmus azureus Sardinia
10486 Eupelmus azureus Switzerland
10222 Eupelmus azureus France
10336 Reikosiella aff. rostrata France
10350 Reikosiella aff. rostrata France
10107 Anastatus aff. temporalis France
10105 Anastatus sidereus France
0.05

Figure S14

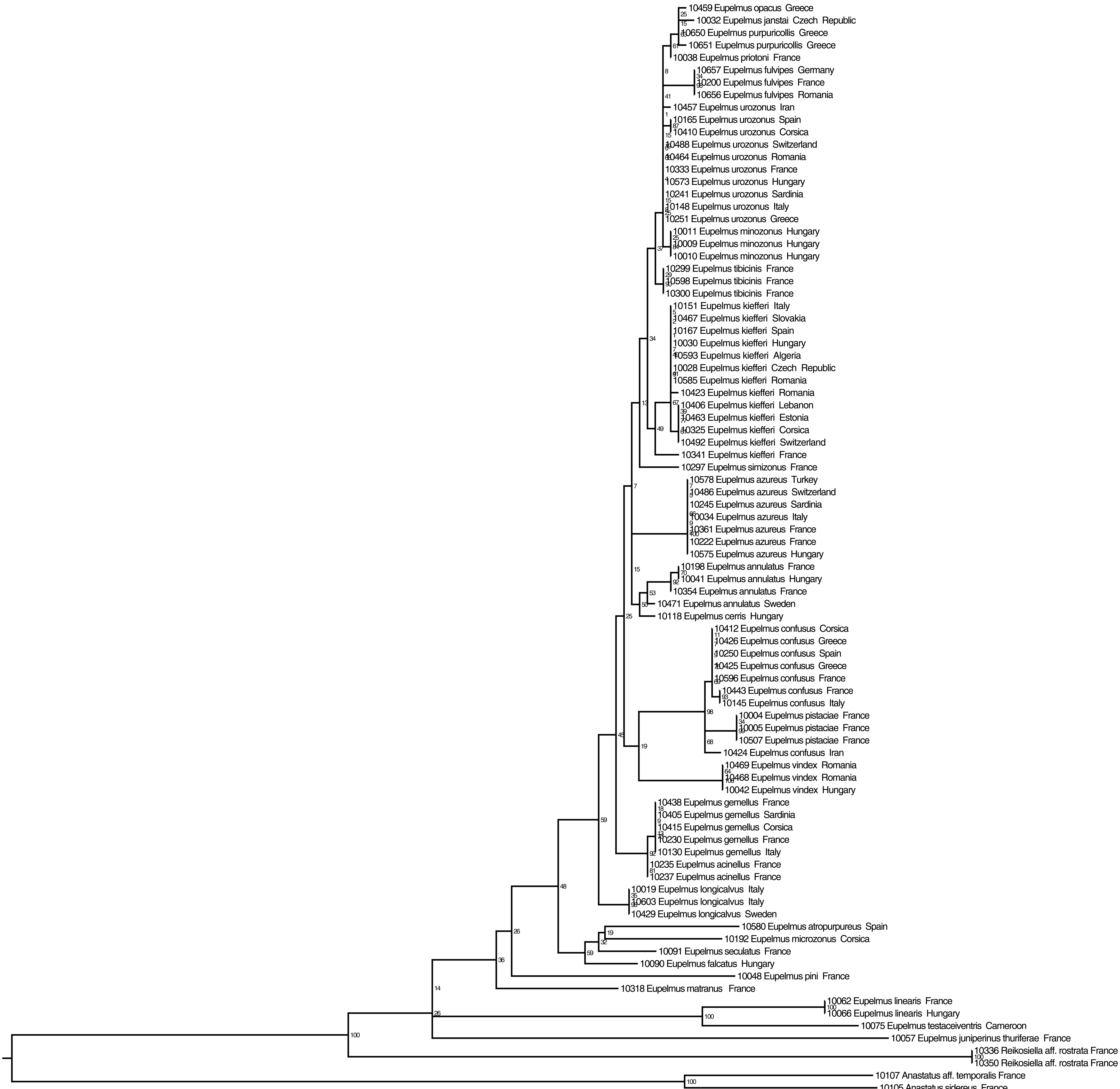
10459 Eupelmus opacus Greece
10032 Eupelmus janstai Czech Republic
10650 Eupelmus purpuricollis Greece
10651 Eupelmus purpuricollis Greece
10038 Eupelmus priotoni France
10657 Eupelmus fulvipes Germany
10200 Eupelmus fulvipes France
10656 Eupelmus fulvipes Romania
10457 Eupelmus urozonus Iran
10165 Eupelmus urozonus Spain
10410 Eupelmus urozonus Corsica
10488 Eupelmus urozonus Switzerland
10464 Eupelmus urozonus Romania
10333 Eupelmus urozonus France
10573 Eupelmus urozonus Hungary
10241 Eupelmus urozonus Sardinia
10148 Eupelmus urozonus Italy
10251 Eupelmus urozonus Greece
10011 Eupelmus minozonus Hungary
10009 Eupelmus minozonus Hungary
10010 Eupelmus minozonus Hungary
10299 Eupelmus tibicinis France
10598 Eupelmus tibicinis France
10300 Eupelmus tibicinis France
10151 Eupelmus kiefferi Italy
10467 Eupelmus kiefferi Slovakia
10167 Eupelmus kiefferi Spain
10030 Eupelmus kiefferi Hungary
10593 Eupelmus kiefferi Algeria
10028 Eupelmus kiefferi Czech Republic
10585 Eupelmus kiefferi Romania
10423 Eupelmus kiefferi Romania
10406 Eupelmus kiefferi Lebanon
10463 Eupelmus kiefferi Estonia
10325 Eupelmus kiefferi Corsica
10492 Eupelmus kiefferi Switzerland
10341 Eupelmus kiefferi France
10297 Eupelmus simizonus France
10578 Eupelmus azureus Turkey
10486 Eupelmus azureus Switzerland
10245 Eupelmus azureus Sardinia
10034 Eupelmus azureus Italy
10361 Eupelmus azureus France
10222 Eupelmus azureus France
10575 Eupelmus azureus Hungary
10198 Eupelmus annulatus France
10041 Eupelmus annulatus Hungary
10354 Eupelmus annulatus France
10471 Eupelmus annulatus Sweden
10118 Eupelmus cerris Hungary
10412 Eupelmus confusus Corsica
10426 Eupelmus confusus Greece
10250 Eupelmus confusus Spain
10425 Eupelmus confusus Greece
10596 Eupelmus confusus France
10443 Eupelmus confusus France
10145 Eupelmus confusus Italy
10004 Eupelmus pistaciae France
10005 Eupelmus pistaciae France
10507 Eupelmus pistaciae France
10424 Eupelmus confusus Iran
10469 Eupelmus vindex Romania
10468 Eupelmus vindex Romania
10042 Eupelmus vindex Hungary
10438 Eupelmus gemellus France
10405 Eupelmus gemellus Sardinia
10415 Eupelmus gemellus Corsica
10230 Eupelmus gemellus France
10130 Eupelmus gemellus Italy
10235 Eupelmus acinellus France
10237 Eupelmus acinellus France
10019 Eupelmus longicalvus Italy
10603 Eupelmus longicalvus Italy
10429 Eupelmus longicalvus Sweden
10580 Eupelmus atropurpureus Spain
10192 Eupelmus microzonus Corsica
10091 Eupelmus seculatus France
10090 Eupelmus falcatus Hungary
10048 Eupelmus pini France
10318 Eupelmus matranus France
10062 Eupelmus linearis France
10066 Eupelmus linearis Hungary
10075 Eupelmus testaceiventris Cameroon
10057 Eupelmus juniperinus thuriferae France
10336 Reikosiella aff. rostrata France
10350 Reikosiella aff. rostrata France
10107 Anastatus aff. temporalis France
10105 Anastatus sidereus France

Figure S15

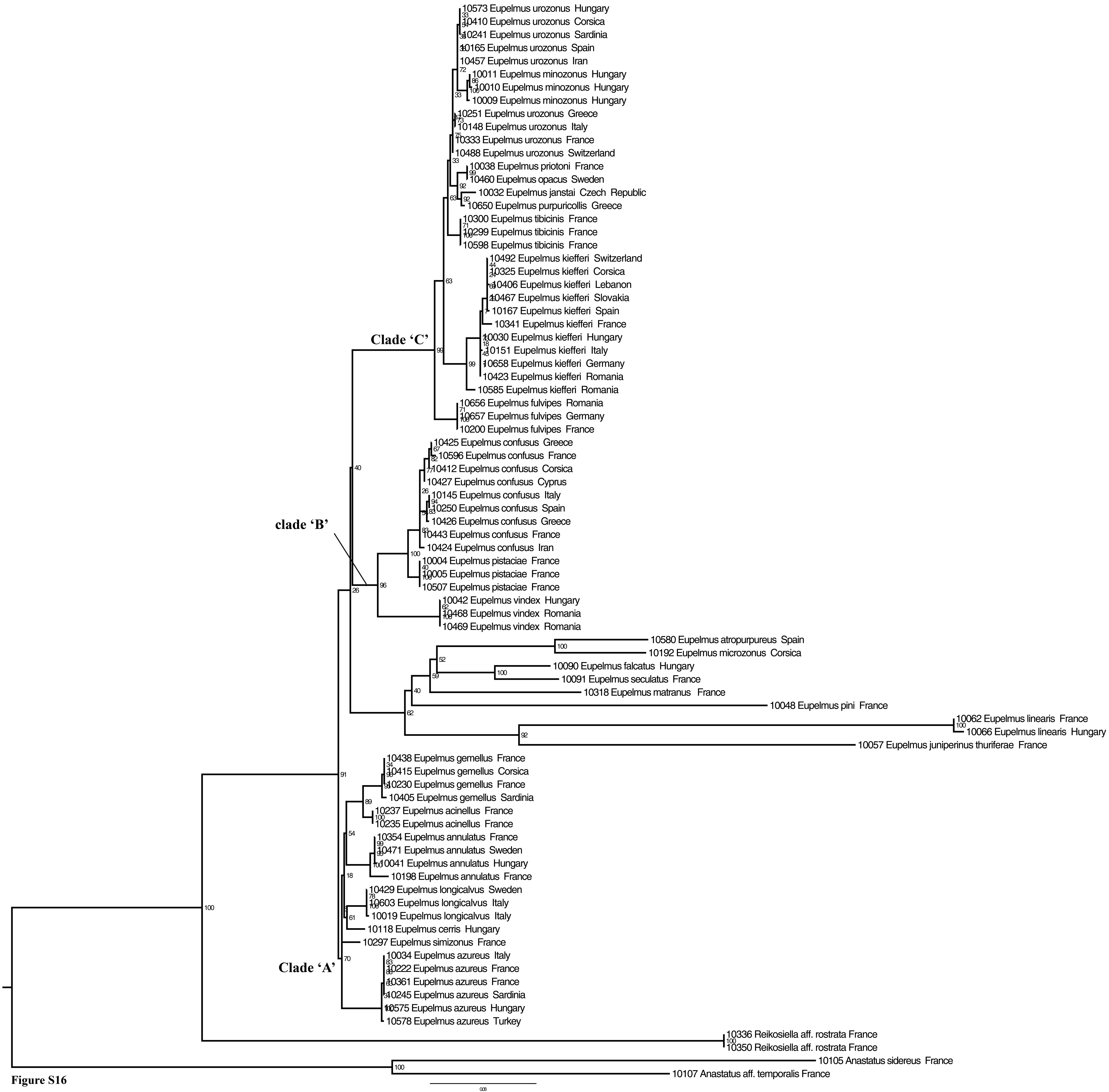
10573 Eupelmus urozonus Hungary
10410 Eupelmus urozonus Corsica
10241 Eupelmus urozonus Sardinia
10165 Eupelmus urozonus Spain
10457 Eupelmus urozonus Iran
10011 Eupelmus minozonus Hungary
10010 Eupelmus minozonus Hungary
10009 Eupelmus minozonus Hungary
10251 Eupelmus urozonus Greece
10148 Eupelmus urozonus Italy
10333 Eupelmus urozonus France
10488 Eupelmus urozonus Switzerland
10038 Eupelmus priotoni France
10460 Eupelmus opacus Sweden
10032 Eupelmus janstai Czech Republic
10650 Eupelmus purpuricollis Greece
10300 Eupelmus tibicinis France
10299 Eupelmus tibicinis France
10598 Eupelmus tibicinis France
10492 Eupelmus kiefferi Switzerland
10325 Eupelmus kiefferi Corsica
10406 Eupelmus kiefferi Lebanon
10467 Eupelmus kiefferi Slovakia
10167 Eupelmus kiefferi Spain
10341 Eupelmus kiefferi France
Clade ‘C’
10030 Eupelmus kiefferi Hungary
10151 Eupelmus kiefferi Italy
10658 Eupelmus kiefferi Germany
10423 Eupelmus kiefferi Romania
10585 Eupelmus kiefferi Romania
10656 Eupelmus fulvipes Romania
10657 Eupelmus fulvipes Germany
10200 Eupelmus fulvipes France
10425 Eupelmus confusus Greece
10596 Eupelmus confusus France
10412 Eupelmus confusus Corsica
10427 Eupelmus confusus Cyprus
10145 Eupelmus confusus Italy
10250 Eupelmus confusus Spain
10426 Eupelmus confusus Greece
clade ‘B’
10443 Eupelmus confusus France
10424 Eupelmus confusus Iran
10004 Eupelmus pistaciae France
10005 Eupelmus pistaciae France
10507 Eupelmus pistaciae France
10042 Eupelmus vindex Hungary
10468 Eupelmus vindex Romania
10469 Eupelmus vindex Romania
10580 Eupelmus atropurpureus Spain
10192 Eupelmus microzonus Corsica
10090 Eupelmus falcatus Hungary
10091 Eupelmus seculatus France
10318 Eupelmus matranus France
10048 Eupelmus pini France
10062 Eupelmus linearis France
10066 Eupelmus linearis Hungary
10057 Eupelmus juniperinus thuriferae France
10438 Eupelmus gemellus France
10415 Eupelmus gemellus Corsica
10230 Eupelmus gemellus France
10405 Eupelmus gemellus Sardinia
10237 Eupelmus acinellus France
10235 Eupelmus acinellus France
10354 Eupelmus annulatus France
10471 Eupelmus annulatus Sweden
10041 Eupelmus annulatus Hungary
10198 Eupelmus annulatus France
10429 Eupelmus longicalvus Sweden
10603 Eupelmus longicalvus Italy
10019 Eupelmus longicalvus Italy
10118 Eupelmus cerris Hungary
10297 Eupelmus simizonus France
10034 Eupelmus azureus Italy
Clade ‘A’
10222 Eupelmus azureus France
10361 Eupelmus azureus France
10245 Eupelmus azureus Sardinia
10575 Eupelmus azureus Hungary
10578 Eupelmus azureus Turkey
10336 Reikosiella aff. rostrata France
10350 Reikosiella aff. rostrata France
10105 Anastatus sidereus France
10107 Anastatus aff. temporalis France

Figure S16

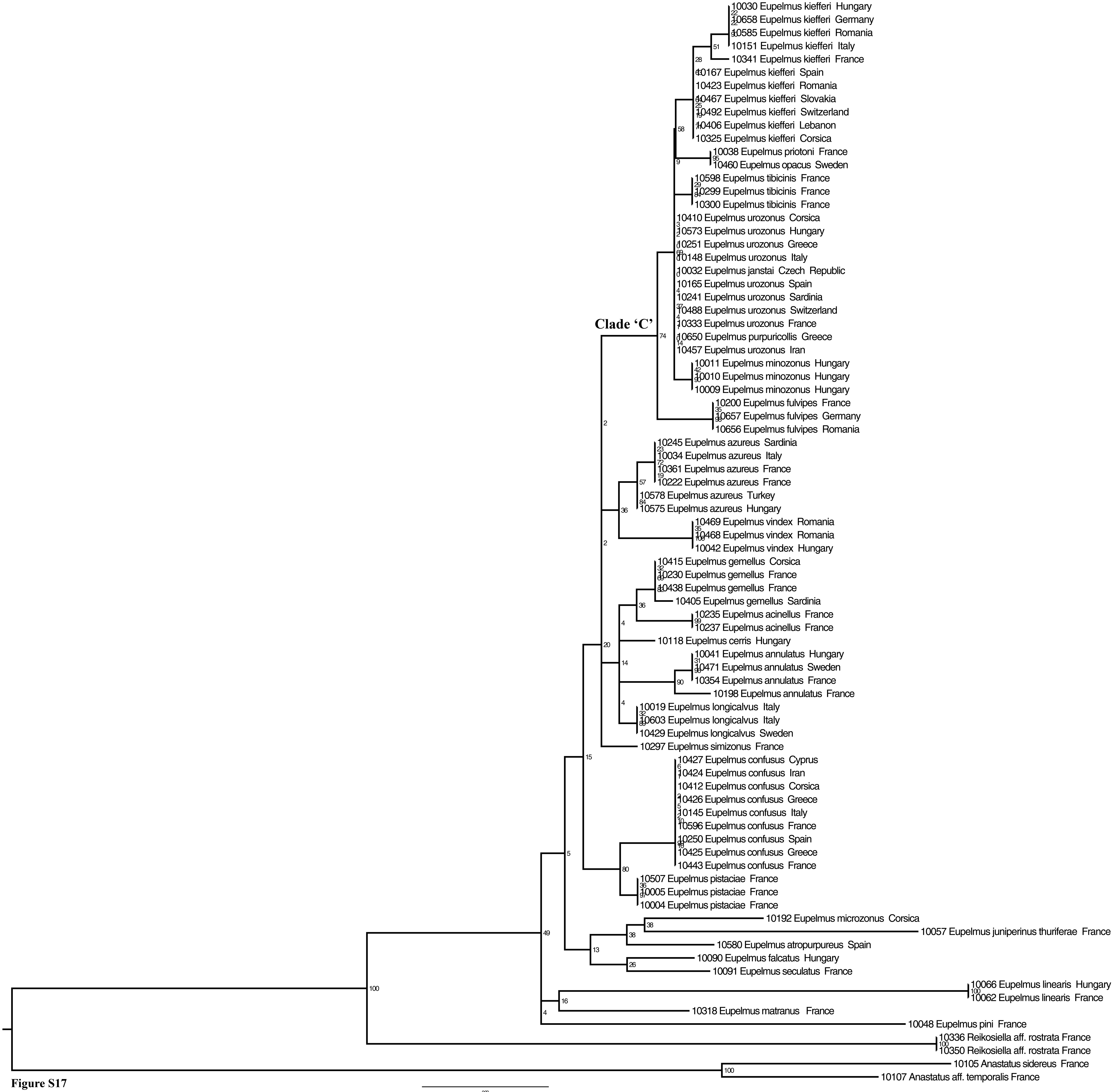
10030 Eupelmus kiefferi Hungary
10658 Eupelmus kiefferi Germany
10585 Eupelmus kiefferi Romania
10151 Eupelmus kiefferi Italy
10341 Eupelmus kiefferi France
10167 Eupelmus kiefferi Spain
10423 Eupelmus kiefferi Romania
10467 Eupelmus kiefferi Slovakia
10492 Eupelmus kiefferi Switzerland
10406 Eupelmus kiefferi Lebanon
10325 Eupelmus kiefferi Corsica
10038 Eupelmus priotoni France
10460 Eupelmus opacus Sweden
10598 Eupelmus tibicinis France
10299 Eupelmus tibicinis France
10300 Eupelmus tibicinis France
10410 Eupelmus urozonus Corsica
10573 Eupelmus urozonus Hungary
10251 Eupelmus urozonus Greece
10148 Eupelmus urozonus Italy
10032 Eupelmus janstai Czech Republic
10165 Eupelmus urozonus Spain
10241 Eupelmus urozonus Sardinia
10488 Eupelmus urozonus Switzerland
10333 Eupelmus urozonus France
10650 Eupelmus purpuricollis Greece
10457 Eupelmus urozonus Iran
10011 Eupelmus minozonus Hungary
10010 Eupelmus minozonus Hungary
10009 Eupelmus minozonus Hungary
10200 Eupelmus fulvipes France
10657 Eupelmus fulvipes Germany
10656 Eupelmus fulvipes Romania
Clade 'C'
10245 Eupelmus azureus Sardinia
10034 Eupelmus azureus Italy
10361 Eupelmus azureus France
10222 Eupelmus azureus France
10578 Eupelmus azureus Turkey
10575 Eupelmus azureus Hungary
10469 Eupelmus vindex Romania
10468 Eupelmus vindex Romania
10042 Eupelmus vindex Hungary
10415 Eupelmus gemellus Corsica
10230 Eupelmus gemellus France
10438 Eupelmus gemellus France
10405 Eupelmus gemellus Sardinia
10235 Eupelmus acinellus France
10237 Eupelmus acinellus France
10118 Eupelmus cerris Hungary
10041 Eupelmus annulatus Hungary
10471 Eupelmus annulatus Sweden
10354 Eupelmus annulatus France
10198 Eupelmus annulatus France
10019 Eupelmus longicalvus Italy
10603 Eupelmus longicalvus Italy
10429 Eupelmus longicalvus Sweden
10297 Eupelmus simizonus France
10427 Eupelmus confusus Cyprus
10424 Eupelmus confusus Iran
10412 Eupelmus confusus Corsica
10426 Eupelmus confusus Greece
10145 Eupelmus confusus Italy
10596 Eupelmus confusus France
10250 Eupelmus confusus Spain
10425 Eupelmus confusus Greece
10443 Eupelmus confusus France
10507 Eupelmus pistaciae France
10005 Eupelmus pistaciae France
10004 Eupelmus pistaciae France
10192 Eupelmus microzonus Corsica
10057 Eupelmus juniperinus thuriferae France
10580 Eupelmus atropurpureus Spain
10090 Eupelmus falcatus Hungary
10091 Eupelmus seculatus France
10066 Eupelmus linearis Hungary
10062 Eupelmus linearis France
10318 Eupelmus matranus France
10048 Eupelmus pini France
10336 Reikosiella aff. rostrata France
10350 Reikosiella aff. rostrata France
10105 Anastatus sidereus France
10107 Anastatus aff. temporalis France

Figure S17

Figure S18

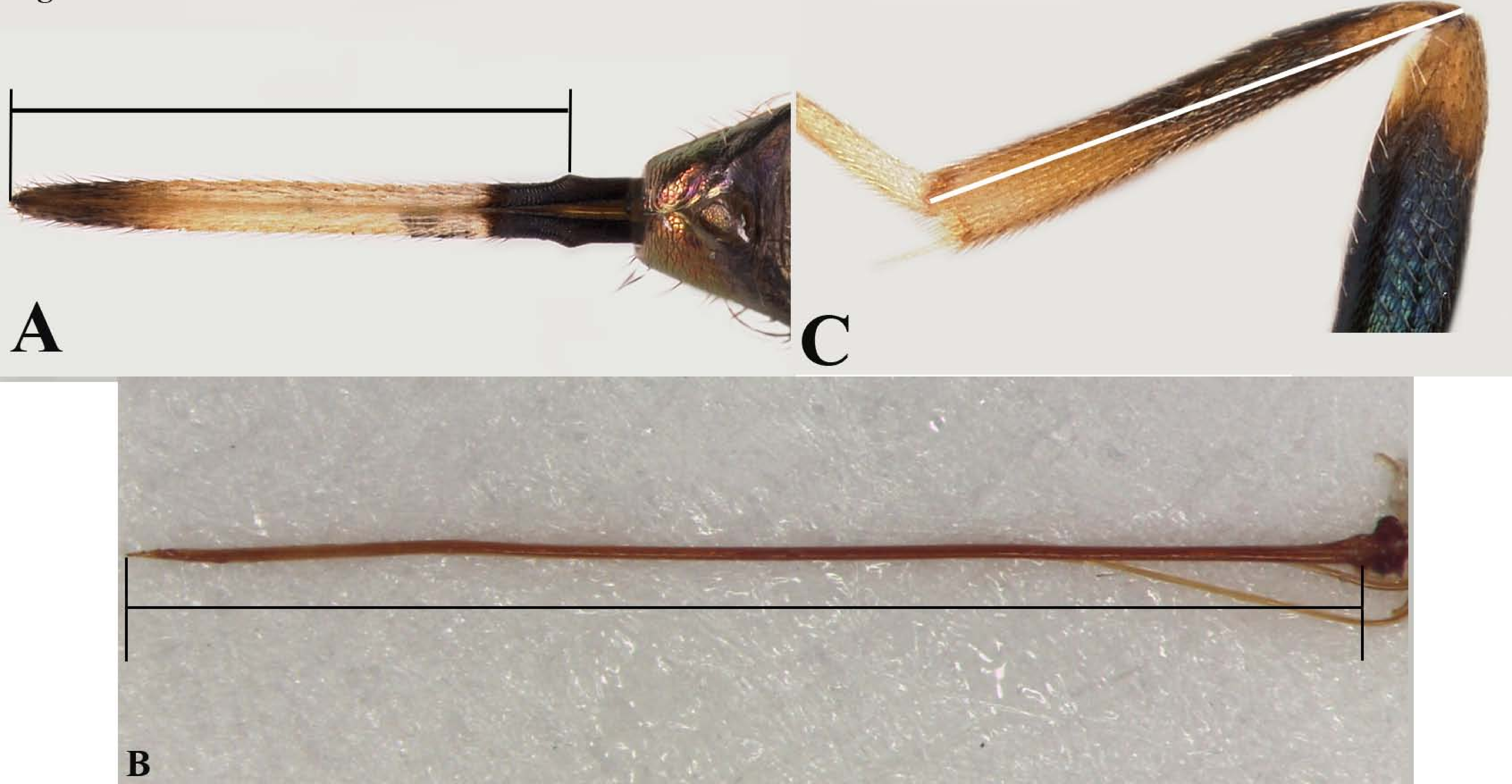

Supplement: Additional file 2: Figure S1. — Trees from a) the ML and b) Bayesian analyses of the combined dataset (without Gblocks cleaning, 9 partitions). Likelihood bootstrap values and posterior probabilities are indicated at nodes. Figure S2. Trees from a) the ML and b) Bayesian analyses of the combined dataset (without Gblocks cleaning, 7 partitions). Likelihood bootstrap values and posterior probabilities are indicated at nodes. Figure S3. Trees from a) the ML and b) Bayesian analyses of the combined dataset (without Gblocks cleaning, 6 partitions). Likelihood bootstrap values and posterior probabilities are indicated at nodes. Figure S4. Trees from a) the ML and b) Bayesian analyses of the combined dataset (without Gblocks cleaning, 2 partitions). Likelihood bootstrap values and posterior probabilities are indicated at nodes. Figure S5. Trees from a) the ML and b) Bayesian analyses of the combined dataset (with Gblocks-default parameters, 9 partitions). Likelihood bootstrap values and posterior probabilities are indicated at nodes. Figure S6. Trees from a) the ML and b) Bayesian analyses of the combined dataset (with Gblocks-default parameters, 7 partitions). Likelihood bootstrap values and posterior probabilities are indicated at nodes. Figure S7. Trees from a) the ML and b) Bayesian analyses of the combined dataset (with Gblocks-default parameters, 6 partitions). Likelihood bootstrap values and posterior probabilities are indicated at nodes. Figure S8. Trees from a) the ML and b) Bayesian analyses of the combined dataset (with Gblocks-default parameters, 2 partitions). Likelihood bootstrap values and posterior probabilities are indicated at nodes. Figure S9. Tree from the ML analysis of the mitochondrial partition. Likelihood bootstrap values (1000 replicates) and posterior probabilities are indicated at nodes. Figure S10. Tree from the ML analysis of the Wg locus. Likelihood bootstrap values (1000 replicates) and posterior probabilities are indicated at nodes. Figure S11. Tree from the ML analysis o [file 12862_2015_571_MOESM2_ESM.pdf]
