# Supplementary material for: Multilocus phylogeny and ecological differentiation of the “Eupelmus urozonus species group” (Hymenoptera, Eupelmidae) in the West-Palaearctic
Source: BMC Evol Biol. 2016 Jan 19;16:13. doi: 10.1186/s12862-015-0571-2 (PMC4717567; doi:10.1186/s12862-015-0571-2)
Supplement: Additional file 3: Table S4. — Summary of information related to the detection of a phylogenetic signal (both host insects and plants). (DOCX 20 kb) [file 12862_2015_571_MOESM3_ESM.docx]

**Additional file 3:Table S4**

| **Developmental ability** | **Distribution of states (no / yes)** | **D-statistics** | **p-value under a random distribution** | **p-value under a Brownian distribution** |
| --- | --- | --- | --- | --- |
| HOST INSECT |  |  |  |  |
| *Order’s level* |  |  |  |  |
| **Coleoptera** | 10 / 3 | 1.17 | 0.51 | 0.29 |
| **Diptera** | 8 / 5 | 1.26 | 0.55 | 0.17 |
| **Hymenoptera** | 3 / 10 | -0.15 | 0.19 | 0.52 |
| **Lepidoptera** | 7 / 6 | 0.26 | 0.22 | 0.48 |
| *Family’s level* |  |  |  |  |
| **Cecidomyiidae (Dip.)** | 9 / 4 | 0.14 | 0.20 | 0.47 |
| **Cynipidae (Hym.)** | 4 / 9 | 0.66 | 0.33 | 0.42 |
| HOST PLANT (Family’s level) |  |  |  |  |
| **Asteraceae** | 9 / 4 | 0.91 | 0.42 | 0.32 |
| **Fagaceae** | 4 / 9 | 1.67 | 0.68 | 0.12 |
| **Rosaceae** | 8 / 5 | 1.06 | 0.48 | 0.23 |
| **Salicaceae** | 9 / 4 | 1.61 | 0.68 | 0.13 |
