# Supplementary material for: Multilocus phylogeny and ecological differentiation of the “Eupelmus urozonus species group” (Hymenoptera, Eupelmidae) in the West-Palaearctic
Source: BMC Evol Biol. 2016 Jan 19;16:13. doi: 10.1186/s12862-015-0571-2 (PMC4717567; doi:10.1186/s12862-015-0571-2)
Supplement: Additional file 4: Table S3. — Summary of Mantel tests used for the comparative analysis dealing with host insects. (DOCX 21 kb) [file 12862_2015_571_MOESM4_ESM.docx]

**Additional file 4: Table S3**

|  | **Phylogeny** | **Morphology** | **Ecology** | | | **Mantel r** | **p-value** |
| --- | --- | --- | --- | --- | --- | --- | --- |
|  | phylogenetic distances | AOS/ROS | Host species | Host family | Host order |  |  |
| **Simple Mantel‘s test** | | | | | | | |
| Extended dataset (19 *Eupelmus* species) | | | | | | | |
|  | X | AOS |  |  |  | 0.09 | 0.39 |
|  | X | ROS |  |  |  | 0.08 | 0.44 |
| Restricted dataset (13 *Eupelmus* species) | | | | | | | |
|  | X |  | X |  |  | 0.02 | 0.85 |
|  | X |  |  | X |  | 0.01 | 0.93 |
|  | X |  |  |  | X | -0.01 | 0.91 |
|  |  | AOS | X |  |  | 0.14 | 0.33 |
|  |  | AOS |  | X |  | -0.06 | 0.68 |
|  |  | AOS |  |  | X | -0.02 | 0.91 |
|  |  | ROS | X |  |  | 0.14 | 0.34 |
|  |  | ROS |  | X |  | -0.06 | 0.68 |
|  |  | ROS |  |  | X | -0.02 | 0.92 |
| **Partial Mantel’s test** (13 *Eupelmus* species) | | | | | | | |
|  | X | AOS | X |  |  | -0.02 | 0.89 |
|  | X | AOS |  | X |  | 0.03 | 0.81 |
|  | X | AOS |  |  | X | -0.01 | 0.96 |
|  | X | ROS | X |  |  | 0.14 | 0.34 |
|  | X | ROS |  | X |  | 0.03 | 0.81 |
|  | X | ROS |  |  | X | -0.01 | 0.96 |
