# Supplementary material for: Multilocus phylogeny and ecological differentiation of the “Eupelmus urozonus species group” (Hymenoptera, Eupelmidae) in the West-Palaearctic
Source: BMC Evol Biol. 2016 Jan 19;16:13. doi: 10.1186/s12862-015-0571-2 (PMC4717567; doi:10.1186/s12862-015-0571-2)
Supplement: Additional file 5: Table S5. — Summary of Mantel tests used for the comparative analysis dealing with host plants. (DOCX 19 kb) [file 12862_2015_571_MOESM5_ESM.docx]

**Additional file 5:Table S5**

|  | **Phylogeny** | **Morphology**  AOS/ROS | **Ecology**  (host plant at family’s level) | **Mantel r** | **p-value** |
| --- | --- | --- | --- | --- | --- |
| **Simple Mantel‘s test** |  |  |  |  |  |
|  | X |  | X | 0.08 | 0.45 |
|  |  | AOS | X | 0.02 | 0.86 |
|  |  | ROS | X | 0.02 | 0.88 |
| **Partial Mantel’s test** |  |  |  |  |  |
|  | X | AOS | X | 0.08 | 0.48 |
|  | X | ROS | X | 0.08 | 0.51 |
